# Syndrome of Combined Pulmonary Fibrosis and Emphysema: An official research statement from American Thoracic Society (ATS), European Respiratory Society (ERS), Japanese Respiratory Society (JRS), and Asociación Latinoamericana de Tórax (ALAT)

**DOI:** 10.1164/rccm.202206-1041ST

**Published:** 2022-08-15

**Authors:** Vincent Cottin, Moises Selman, Yoshikazu Inoue, Alyson W. Wong, Tamera J. Corte, Kevin R. Flaherty, MeiLan K. Han, Joseph Jacob, Kerri A. Johannson, Masanori Kitaichi, Joyce S. Lee, Alvar Agusti, Katerina M. Antoniou, Pauline Bianchi, Fabian Caro, Matias Florenzano, Liam Galvin, Tae Iwasawa, Fernando J. Martinez, Rebecca L. Morgan, Jeffrey L. Myers, Andrew G. Nicholson, Mariaelena Occhipinti, Venerino Poletti, Margaret L. Salisbury, Don D. Sin, Nicola Sverzellati, Thomy Tonia, Claudia Valenzuela, Christopher J. Ryerson, Athol U. Wells

**Affiliations:** 1 National Reference Center for Rare Pulmonary Diseases, Louis Pradel Hospital, Hospices Civils de Lyon, University of Lyon, INRAE, Lyon, France; 2 Instituto Nacional de Enfermedades Respiratorias “Ismael Cosío Villegas”, Mexico City, Mexico; 3 Osaka, Japan; 4 University of British Columbia, Vancouver, Canada; 5 Royal Prince Alfred Hospital and University of Sydney, Sydney, Australia; 6 University of Michigan, Ann Arbor, USA; 7 University of Michigan, Ann Arbor, USA; 8 University College London, London, United Kingdom; 9 Department of Medicine and Community Health Sciences, University of Calgary, Calgary, AB, Canada; 10 NHO Minami Wakayama Medical Center, Tanabe, Japan; 11 University of Colorado Denver Anschutz Medical Campus, School of Medicine, Aurora, CO, USA; 12 Respiratory Institute, Hospital Clinic, University of Barcelona, IDIBAPS, CIBERES, Barcelona, Spain; 13 Laboratory of Molecular and Cellular Pneumonology, Department of Respiratory Medicine, University of Crete, Heraklion, Greece; 14 Pulmonary Fibrosis Foundation; 15 Hospital de Rehabilitación Respiratoria "María Ferrer", Buenos Aires, Argentina; 16 Instituto Nacional Del Tórax, Santiago, Chile; 17 European idiopathic pulmonary fibrosis and related disorders federation; 18 Kanagawa Cardiovascular and Respiratory Center, Yokohama, Japan; 19 New York, USA; 20 McMaster University, Hamilton, Canada; 21 University of Michigan, Ann Arbor, USA; 22 Royal Brompton and Harefield Hospitals, Guy’s and St Thomas’ NHS Foundation Trust and National Heart and Lung Institute, Imperial College, London, United Kingdom; 23 Radiomics, Liege, Belgium; 24 University of Bologna, Ospedale Morgagni, Forlì, Italy; 25 Vanderbilt University Medical Center, Nashville, USA; 26 University of British Columbia, Vancouver, Canada,; 27 Scienze Radiologiche, Department of Medicine and Surgery, University of Parma, Italy; 28 Institute of Social and Preventive Medicine, University of Bern, Switzerland; 29 Pulmonology Department, Hospital Universitario de la Princesa, Departamento Medicina, Universidad Autónoma de Madrid, 28049 Madrid, Spain; 30 University of British Columbia, Vancouver, Canada; 31 Imperial college, London, UK

**Keywords:** 9.23 Interstitial Lung Disease, Fibrosis, interstitial lung diseases, emphysema, diagnosis, management

## Abstract

**Background:**

The presence of emphysema is relatively common in patients with fibrotic interstitial lung disease. This has been designated combined pulmonary fibrosis and emphysema (CPFE). The lack of consensus over definitions and diagnostic criteria has limited CPFE research.

**Goals:**

The objectives of this taskforce were to review the terminology, definition, characteristics, pathophysiology, and research priorities of CPFE, and to explore whether CPFE is a syndrome.

**Methods:**

This research statement was developed by a committee including 19 pulmonologists, 5 radiologists, 3 pathologists, 2 methodologists, and 2 patient representatives. The final document was supported by a focused systematic review that identified and summarized all recent publications related to CPFE.

**Results:**

This taskforce identified that patients with CPFE are predominantly male, with history of smoking, severe dyspnea, relatively preserved airflow rates and lung volumes on spirometry, severely impaired diffusion capacity for carbon monoxide, exertional hypoxemia, frequent pulmonary hypertension, and a dismal prognosis. The committee proposes to identify CPFE as a syndrome given the clustering of pulmonary fibrosis and emphysema, shared pathogenetic pathways, unique considerations related to disease progression, increased risk of complications (pulmonary hypertension, lung cancer, mortality), and implications for clinical trial design. There are varying features of interstitial lung disease and emphysema in CPFE. The committee offers a research definition and classification criteria, and proposes that studies on CPFE include a comprehensive description of radiologic and, when available, pathological patterns including some recently described patterns such as smoking-related interstitial fibrosis.

**Conclusions:**

This statement delineates the syndrome of CPFE and highlights research priorities.

## Introduction

Emphysema is relatively common in patients with fibrotic interstitial lung disease (fILD), including idiopathic pulmonary fibrosis (IPF), and is designated “combined pulmonary fibrosis and emphysema” (CPFE)^1,2^. Despite its clinical significance and a number of published series ^3^, CPFE remains poorly understood. Imaging features of CPFE vary in both fILD and emphysema, and not all cases correspond to IPF with emphysema. Similarly, the spectrum of pathologic features includes recently described patterns such as airspace enlargement with fibrosis (AEF)^4^ and smoking-related interstitial fibrosis (SRIF)^5^. Lack of consensus on criteria for CPFE has limited our ability to compare cohorts and draw consistent conclusions about the features, outcomes, and optimal management of these patients ^3^. No consensus exists on whether CPFE is a syndrome (i.e. a cluster of clinical and radiologic manifestations with clinically relevant implications and/or major pathogenetic significance)^6^ or a distinct entity. In essence, CPFE remains relatively understudied, with no specific treatment.

The objectives of this taskforce were: 1) to describe the terminology, definition, etiologies, features, comorbidities, and outcomes of CPFE; and 2) to provide a consensus definition and terminology of CPFE, determine whether it represents a syndrome, describe its management, and identify research priorities.

## Methods

This research statement was developed by a committee of experts appointed by the American Thoracic Society (ATS), the European Respiratory Society (ERS), the Japanese Respiratory Society (JRS), and the Asociación Latinoamericana del Tórax (ALAT). The committee included 19 pulmonologists, 5 radiologists, 3 pathologists, 2 methodologists, and 2 patient representatives. Potential conflicts of interest were disclosed and managed in accordance with the ATS policies and procedures. The taskforce communicated during two face-to-face meetings, and via e-mail and teleconferences. Sections of the document were elaborated by subgroups, each with a leader responsible for writing. The final manuscript was approved by all panelists.

The search strategy was published previously and the search was updated on December 1, 2021 ^3^ (online supplement). We searched MEDLINE and EMBASE databases for all original research articles published in English between January 1, 2000 and December 1, 2021, which included patients with both pulmonary fibrosis and emphysema in any distribution (Tables E1 and E2). All forms of original research were included (e.g., randomized control trials and observational studies), apart from case series containing <10 patients. Screening was performed by two reviewers using pre-determined criteria and disagreements were resolved by consensus with a third reviewer (Figure E1).

## Historical perspective

The milestones of the description of CPFE are listed in table 1 and described in the online supplement. Since these initial publications, several series cited later in this document have contributed to a more complete description of CPFE, and etiological factors other than smoking have been identified.

## Epidemiology

Emphysema is common in current or former smokers with fILD. Prevalence estimates of CPFE vary depending on the population studied and the definition used, ranging from 8-67% of patients with IPF ^24–34^. There may be geographical variation in prevalence, with the highest estimates from Asia and Greece, and lower estimates in the United States. These differences may be attributable to differing genetic susceptibility, smoking rates or definitions of CPFE. CPFE is reported in 26-54% of patients with idiopathic interstitial pneumonia ^35,36^, with higher prevalence in those requiring hospital admission (45-71%)^37,38^. The prevalence is also higher in patients with lung cancer and idiopathic interstitial pneumonia, including IPF ^39,40^.

The prevalence of CPFE in the general population is unknown, as most data come from patients with an indication for chest computed tomography (CT). CPFE as previously defined ^1^ was identified radiographically in 7.3% of males who underwent high-resolution CT (HRCT) of the chest (indication unknown)^41^ and in 2.8% of all HRCTs done at a single center in Korea ^42^. In patients with resected lung cancer, CPFE was found in 3-10% of patients ^38,43–45^(Table 2); however, another lung cancer screening cohort found a much lower prevalence at 0.04% ^36^.

## Etiologies

### Exposures and diseases

Cigarette smoking and male sex are consistently associated with CPFE. CPFE occurs nine times more often in males, and this discrepancy is not wholly attributable to a greater history of smoking in males ^46^. Almost all patients with CPFE report a history of smoking, with an average exposure of 40 pack-years, with the notable exceptions of some patients with connective tissue disease (CTD) or fibrotic hypersensitivity pneumonitis (fHP)^47,48^ who on average have less smoking exposure ^49–53^(Figure E2). A smoking history is more common in CPFE than in isolated IPF ^24,32,34,38,43,45,50,54–59^ or systemic sclerosis-associated ILD ^49^. The association between CPFE and number of pack-years suggests a dose response effect ^28,55,58,60,61^. Emphysema generally precedes fILD when the data are available, although there are some exceptions to this, particularly if considering interstitial lung abnormality as an early form of ILD ^1^.

CPFE can occur in non-smokers especially in CTD, suggesting CTD itself as a risk factor ^28,51,52^. In 470 patients with systemic sclerosis, 43 had CPFE on chest CT, including 24 (58%) who had never smoked ^62^. Approximately 5-10% of patients with systemic sclerosis-associated ILD have radiological findings of CPFE ^49,51,63,64^. In 116 never smokers with rheumatoid arthritis-associated ILD (RA-ILD), emphysema was present on HRCT in 27% ^52^. CPFE is also reported in systemic vasculitis, particularly microscopic polyangiitis ^65,66^. Of 150 consecutive patients with RA, 12 (8%) had both ILD and emphysema ^67^; however, in patients with rheumatoid lung, the reported prevalence of emphysema is as high as 48% ^28,68^. Emphysema on HRCT was less extensive in CTD-associated usual interstitial pneumonia (UIP) than in IPF (idiopathic UIP)^69^. IPF patients with CPFE are more likely to have positive antinuclear antibodies or p-antineutrophil cytoplasmic antibodies than IPF patients without emphysema ^1,25^.

Multiple occupational and inhalational exposures are associated with CPFE ^70–73^(Table 3). CPFE is reported in patients with asbestosis and silicosis, occasionally in lifelong non-smokers ^74–77^. Interestingly, emphysema occurs in 7-23% of patients with fHP ^47,78^. Occupational exposure to vapors, dusts, gases, and fumes is associated with more extensive radiologic emphysema after adjusting for smoking pack-years ^79^.

### Genetic predisposition and aging

Genetic predisposition in combination with risk factors including smoking or exposure to other aero-contaminants, may predispose individuals to develop both fibrosis and emphysema ^2^, both of which involve aging and cell senescence ^107–111^. Genetic predilections for CPFE are not well understood, with only a few cases reported of mutations carrying a Mendelian risk of CPFE or IPF. CPFE has been reported in patients carrying mutations in genes associated with surfactant (see online supplement)^94–99^ or telomeres ^89–93^. Shorter telomeres are associated with both chronic obstructive pulmonary disease (COPD) and IPF ^112^, and are, thus, likely associated with CPFE ^2,46^, although this requires further study ^113^. If confirmed, CPFE would represent a model of smoking-induced, telomere-related, lung disease. Epigenetic alterations may also be important ^114^.

## Clinical manifestations and comorbidities

Patients with CPFE have a mean age of approximately 65-70 years ^1,46^ (comparable to IPF and COPD), with 73-100% male predominance ^1,24–33,38,43,45,55–57,60,61^. Symptoms include exertional dyspnea and cough ^1,41^. Patients with CPFE and pulmonary hypertension (PH) have significant exertional breathlessness, with the majority having a New York Heart Association functional class of III or IV ^115^.

In CPFE, the two most prominent comorbidities are lung cancer and PH, discussed in the outcomes section below. Other comorbidities include coronary artery disease, peripheral vascular disease, and diabetes ^38,116^, although it is not known whether these diseases are more prevalent in CPFE than in IPF without emphysema ^42,60^. Differences in sample size, study design (retrospective), and methods for identification and documentation of comorbidities contribute to uncertainties. Prospective studies with standardized data collection methods and case definitions are required.

## Lung function

Patients with CPFE have limited exercise capacity, severely impaired DLco and transfer coefficient (Kco)^1,46,117–119^, contrasting with relatively preserved airflow rates and lung volumes. The FVC/DLco ratio is increased in most patients ^51^.

Compared to isolated IPF, patients with CPFE have higher lung volumes (FVC and TLC), generally comparable FEV1, higher residual volume (RV), lower DLco, lower Kco, and lower PaO2 ^17,24,26,31–33,37,59,60,119–125^, even with adjustment for the extent of fibrosis ^17,121^(Table 4). The mean FEV_1_/FVC ratio is usually normal or slightly reduced, may rise with progression of fibrosis, but is typically lower than in isolated IPF where it is usually increased (e.g. > 0.80)^26,120^. Comparison of physiology between CPFE and isolated IPF may be hampered by differences between studies in the severity of both emphysema and fibrosis, despite attempts to adjust for severity ^24^.

Compared to COPD, patients with CPFE have relatively preserved FEV_1_ and FEV_1_/FVC, less hyperinflation, and lower DLco ^126^. A minority of the 132 patients (36% pooled prevalence) from three previous studies had TLC < 80% predicted ^1,50,115^, while only 41% had FEV_1_/FVC < 0.70. Of these, 11% had FEV_1_ > 80% predicted, corresponding to Global initiative for Obstructive Lung Disease stage 1, 37% were classified as stage 0 (FEV_1_/FVC ≥ 0.70 and FEV_1_ ≥ 80% of predicted), and 22% were unclassified (with FEV_1_/FVC ≥ 0.70 and FEV_1_ < 80% of predicted). In another study, smokers with emphysema were less likely to meet functional criteria for COPD if ILD was present on imaging ^127^. Thus, the relative preservation of spirometric values may lead to underdiagnosis of chronic lung disease if only spirometry is obtained.

The relative preservation of flow rates and lung volumes is attributed to the counterbalancing effects of the restrictive physiology from pulmonary fibrosis (presumably increased elastic recoil and prevention of expiratory airway collapse by traction forces) and the effects of emphysema on the airways. Thus, in CPFE, FEV_1_/FVC can actually improve to normal values as fibrotic disease progresses, despite worsening dyspnea and DLco ^128^, and contrary to COPD ^126^. TLC correlates positively with emphysema extent on CT, and negatively with fibrosis extent. Conversely, FEV_1_/FVC correlates negatively with emphysema extent on CT, and positively with fibrosis extent ^129^. Compared to isolated fILD, patients with CPFE have lower whole-breath inspiratory and expiratory resistance based on analysis of respiratory impedance by multi-frequency forced oscillation technique, further supporting the hypothesis of “normalization” of lung mechanics ^130^. Conversely, both disease components reduce alveolar capillary gas exchange through either decreased capillary blood volume or alveolar membrane thickening, resulting in greater reductions in DLco.

Severe decrease in arterial oxygen saturation and hypoxemia at exercise is very common in CPFE, especially when complicated by severe PH ^1,115,119^. Hence, exercise limitation with decrease in oxygen saturation ^119^, and isolated ^131^ and/or severe ^132^ reduction in DLco or Kco, contrasting with a mild ventilatory defect, should raise the suspicion of CPFE and/or PH. Compared to isolated IPF, patients with CPFE have lower exercise capacity despite less extensive fibrosis on HRCT ^133,134^. Exertional dyspnea is the key limiting factor, related to poor ventilatory efficiency and, presumably, increased dead space in hypoperfused areas ^133^. Hypercapnia occurs only very late in the disease course. A similar functional profile is observed when CPFE occurs in CTD ^49–52^ or fHP ^47^.

Importantly, the presence of significant emphysema impacts on serial lung volume trends, attenuating serial lung volume decline due to progressive fibrosis. Patients with CPFE experience a slower decline in FVC than patients with isolated IPF ^26,29,124^, whereas decline in DLco and increase in the Composite Physiologic Index (CPI), which quantifies functional impairment due to IPF whilst excluding the functional impact of emphysema, are less affected ^29,60^. In an analysis of patients with IPF from two randomized controlled trials, emphysema extent ≥15% was associated with reduced FVC decline over 48 weeks compared to those with either no emphysema or emphysema extent <15% ^29^.

Consequently, no optimal parameter has been validated to monitor disease progression in CPFE. Changes in FVC, commonly used to monitor IPF progression ^135^, are not reliable indicators of disease progression in patients with CPFE ^26,29,124^, which has implications for clinical trial design ^2,136^. Serial change in DLco may be a helpful marker of disease progression but is additionally affected by other factors including vasculopathy, hemoglobin concentration, and measurement variation. Serial change in CPI is not validated for monitoring ILD progression. A FEV1%FVC ratio >1.2 at baseline ^137^ and a decline in FEV_1_ by 10% or more at 6 or 12 months ^138^ were associated with a poor outcome, but these observations warrant confirmation. In clinical practice, a decline in one or several of the above-mentioned functional parameters may be observed in individual patients. In summary, the committee therefore suggests that disease progression in CPFE be monitored using a combination of clinical, imaging, and multiple functional parameters, with less emphasis on FVC trends than in the monitoring of ILD without concurrent emphysema.

## Imaging features

### Overview

CPFE is characterized by the presence of emphysema and interstitial fibrosis, with a wide variety of appearances on chest HRCT.

**Figure 1:**
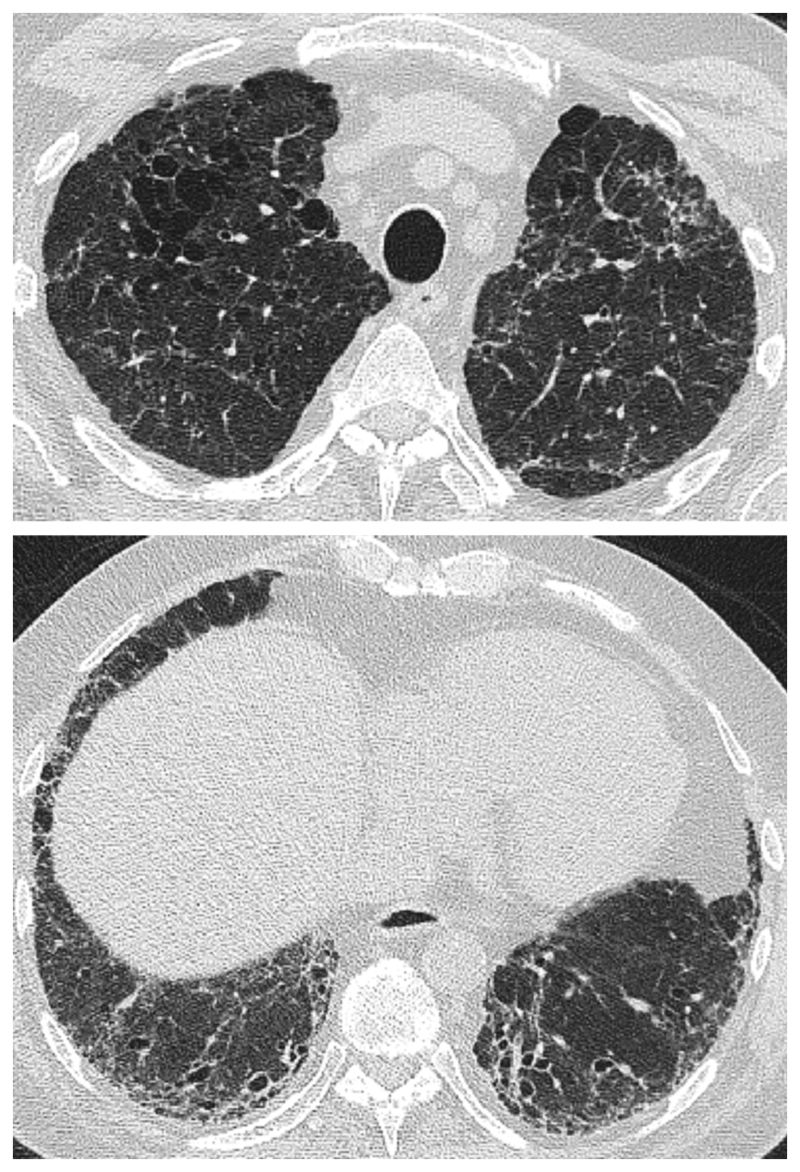
HRCT showing a typical distribution of disease seen in combined pulmonary fibrosis and emphysema (separate emphysema and fibrosis pattern). Paraseptal and centrilobular emphysema is localized to the upper lobes, whilst fibrosis characterized by traction bronchiectasis is localized to the lower lobes.

**Figure 2:**
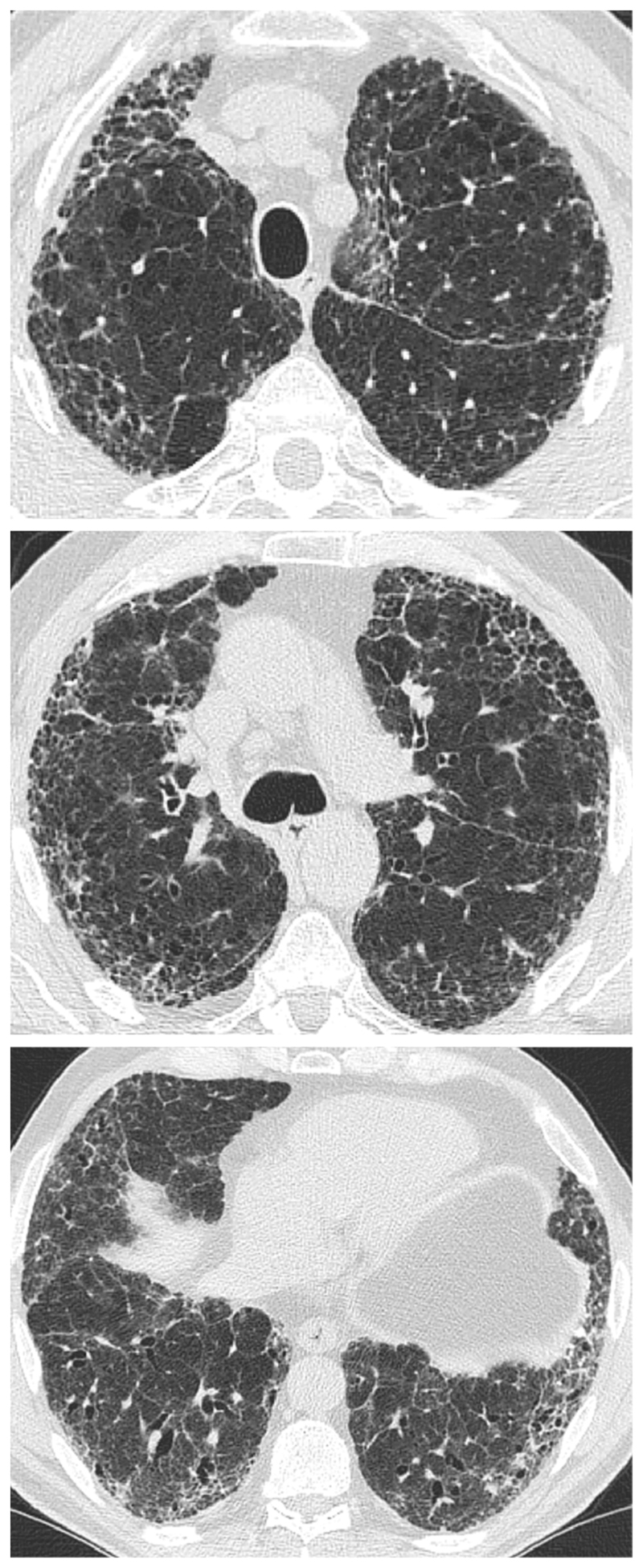
HRCT showing a typical distribution of disease seen in combined pulmonary fibrosis and emphysema (progressive transition pattern). A predominant pattern of centrilobular emphysema is seen in the upper lobes, extending to the midzones of the lungs in a 72-year-old patient with idiopathic pulmonary fibrosis. No emphysema is seen in the lower zones. The appearances are in keeping with a progressive transition pattern of combined pulmonary fibrosis and emphysema

**Figure 3:**
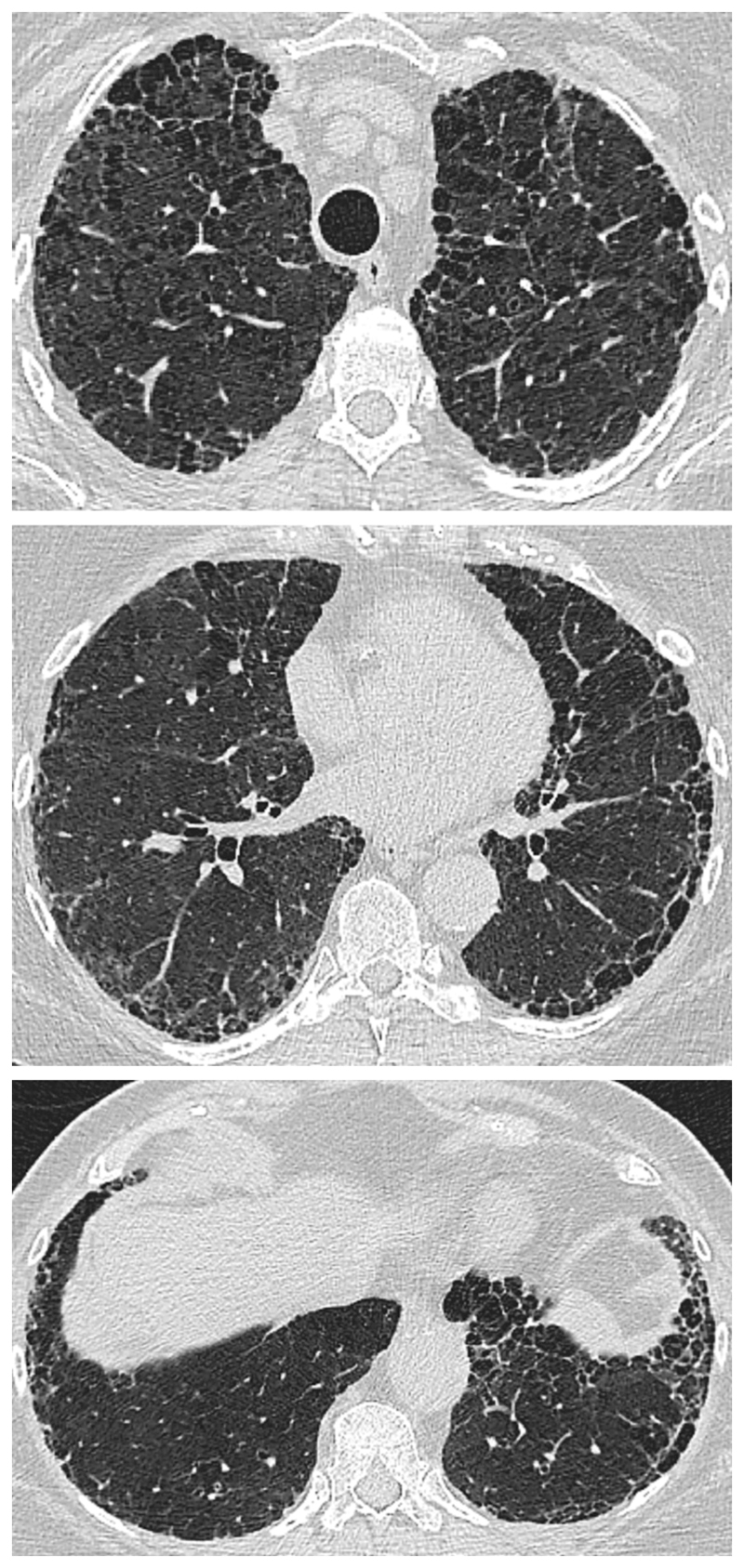
HRCT showing a typical distribution of disease seen in combined pulmonary fibrosis and emphysema (paraseptal emphysema pattern). In this 67 year-old male diagnosed with idiopathic pulmonary fibrosis, extensive isolated paraseptal and centrilobular emphysema is present in the upper zones. Whilst the centrilobular emphysema is mostly isolated in the midzones, paraseptal emphysema is increasingly admixed resembling honeycomb cysts in the left lung. Within the left lower lobe, paraseptal emphysema mimicking honeycomb cysts lies adjacent to more centrally placed irregularly shaped centrilobular emphysema (arrow).

**Figure 4:**
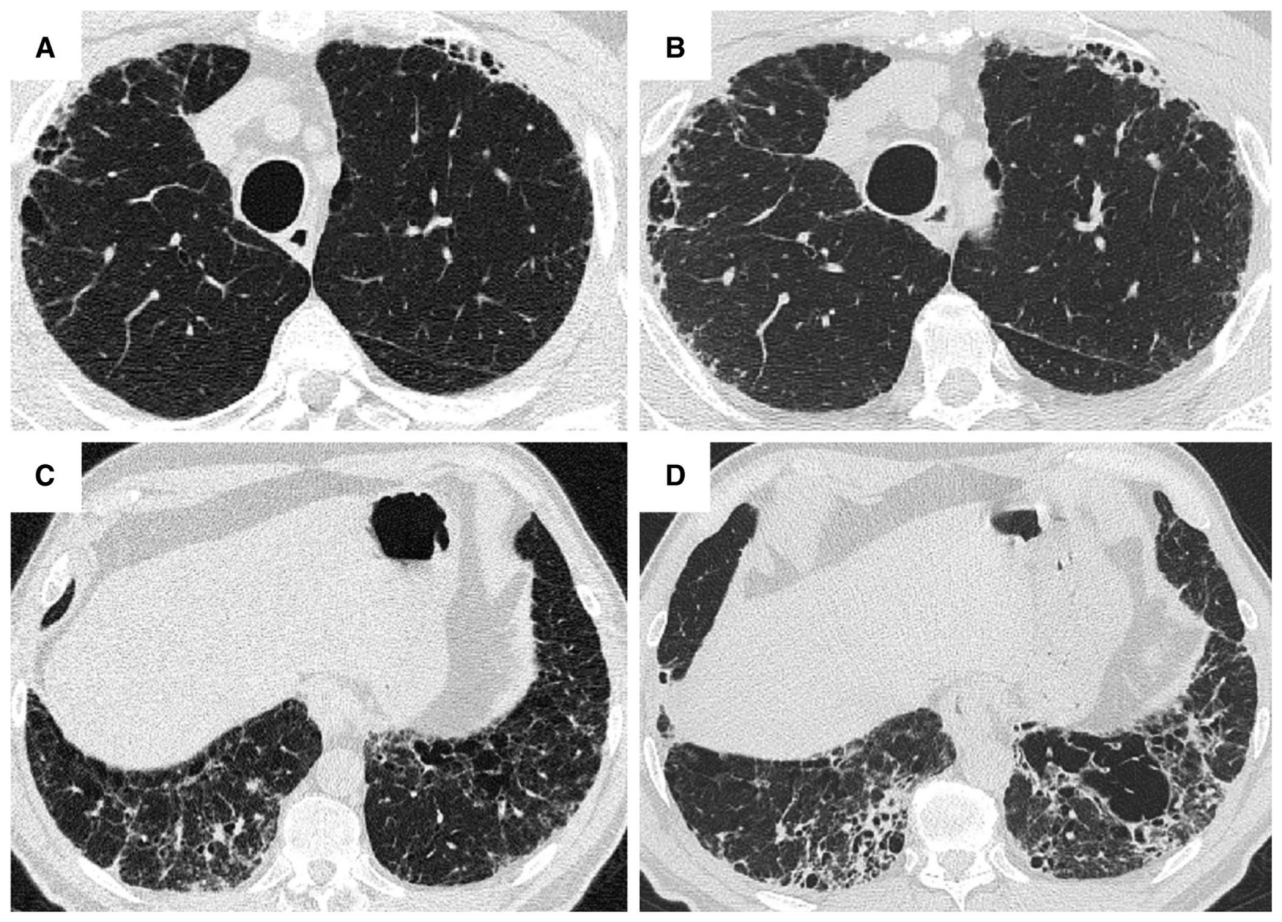
Chest HRCT showing worsening of admixed destructive emphysema (combined pulmonary fibrosis and emphysema, admixed pattern). In the pair of axial images of the upper (A, B) and lower zones (C, D), taken 2 years apart (A, C: baseline; B, D: follow-up) in a 66-year old male patient with idiopathic pulmonary fibrosis, isolated emphysema in the right upper lobe becomes admixed with fibrosis over time. In the left lower lobe, centrally placed emphysema becomes pulled apart (‘traction emphysema’) and expands as the surrounding fibrosis evolves.

**Figure 5:**
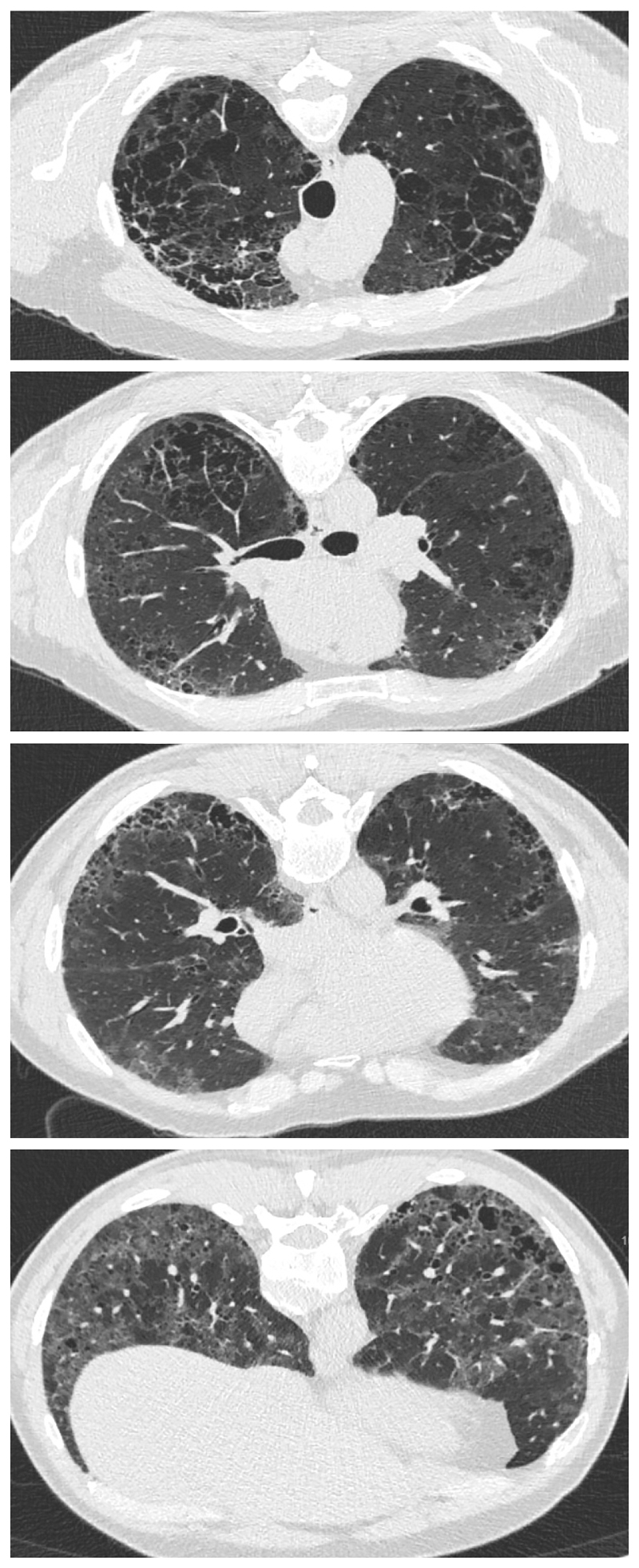
Chest HRCT showing emphysema admixed with desquamative interstitial pneumonia confirmed by lung biopsy (combined pulmonary fibrosis and emphysema, admixed pattern at HRCT). Areas of low attenuation are admixed with ground glass opacities (high attenuation) and thickening of peri-emphysematous areas.

**Figure 6:**
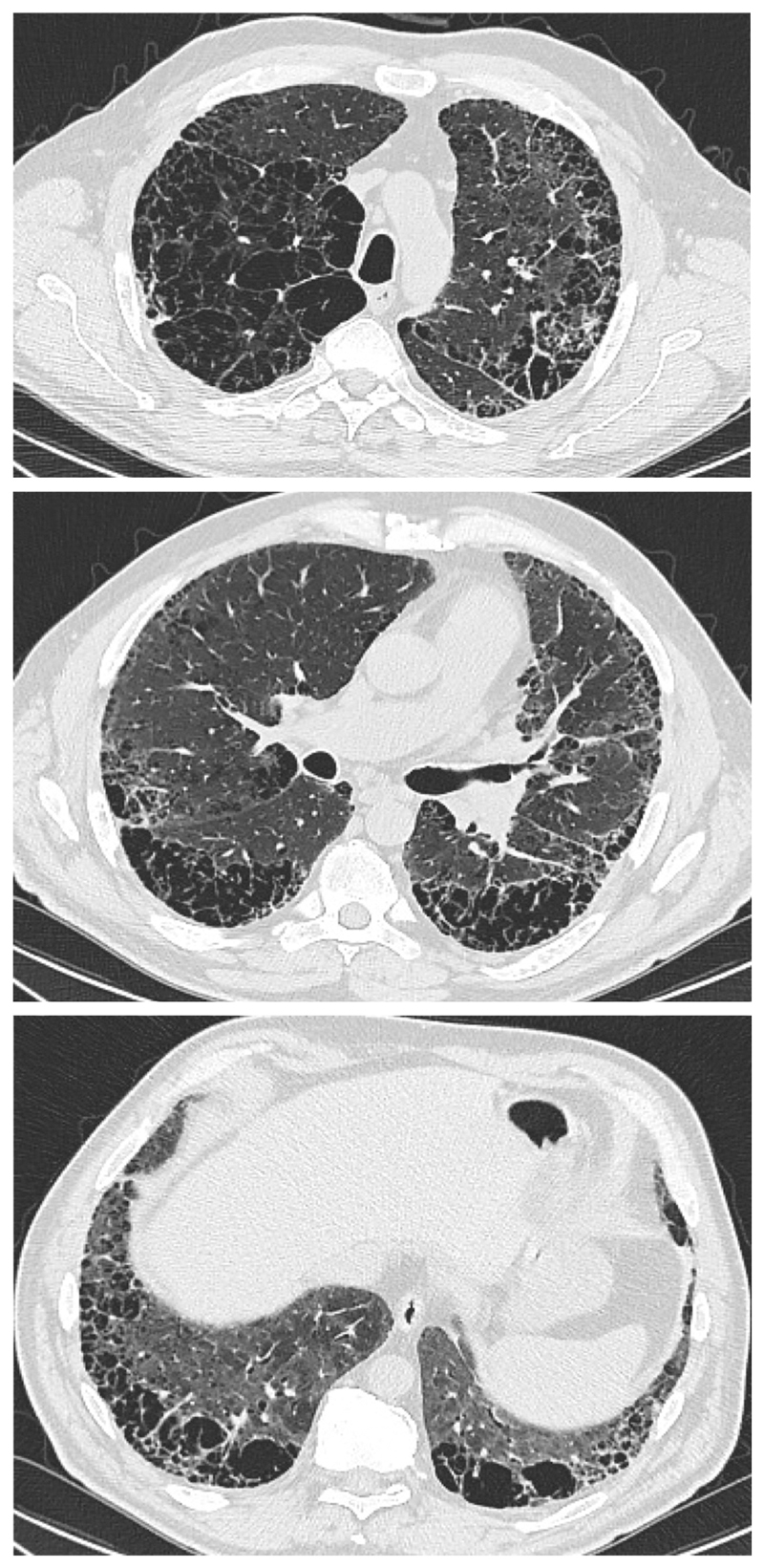
Chest HRCT showing admixed emphysema and fibrosis with thick-walled large cysts (combined pulmonary fibrosis and emphysema, thick-walled large cysts pattern). Histopathology demonstrated predominantly smoking-related interstitial fibrosis (SRIF).

**Figure 7:**
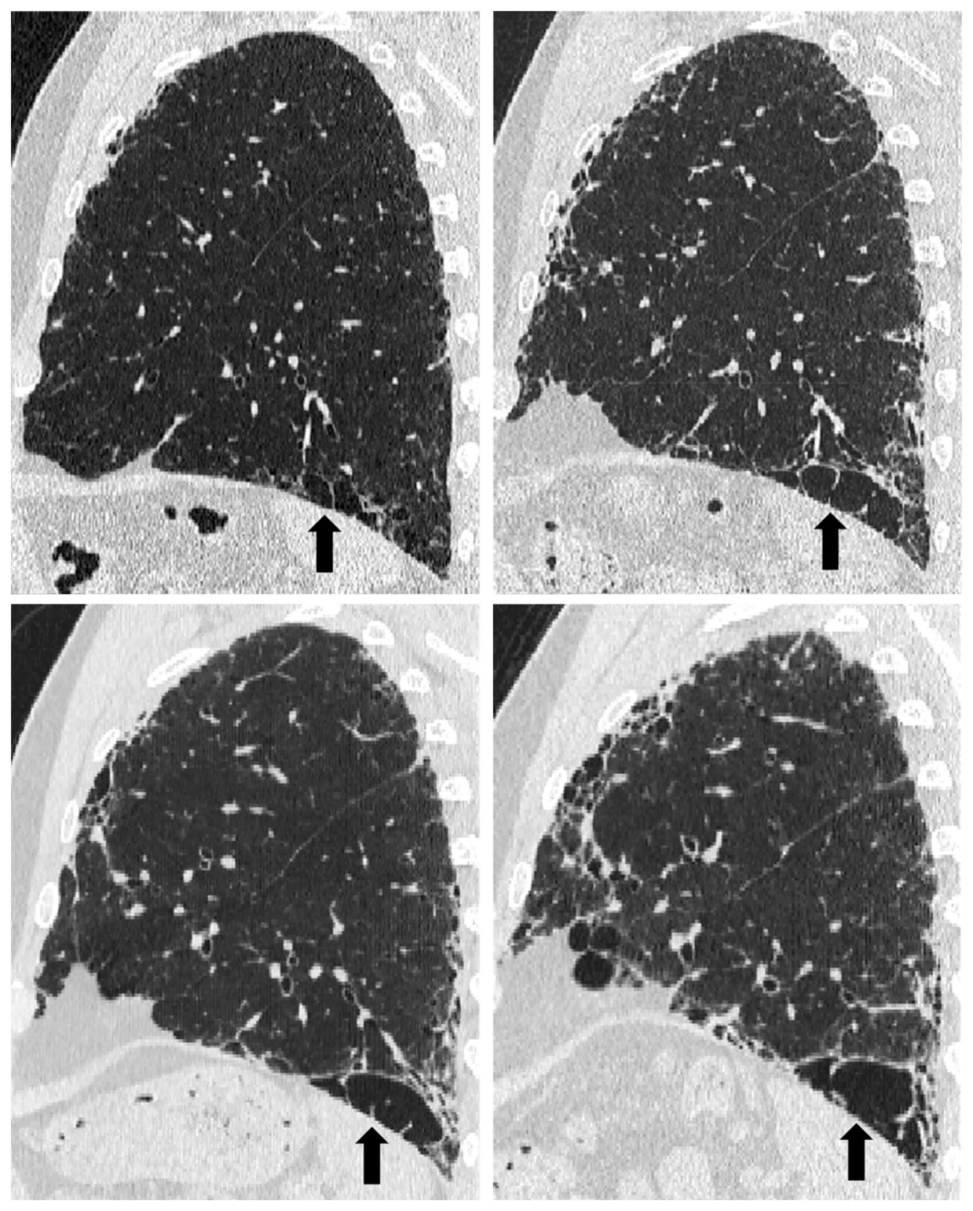
Sagittal CT images of the lungs performed over the course of 4 years in a 66-year-old male ex-smoker diagnosed with idiopathic pulmonary fibrosis and emphysema (combined pulmonary fibrosis and emphysema, thick-walled large cysts pattern). In the top left image low-attenuation lesions without clearly visible walls in keeping with emphysema (arrow) are visible adjacent to the diaphragm and lie within fibrotic regions of lung. Over the next 4 years, as the fibrosis matures, the low-attenuation lesions coalesce (arrow) and enlarge in size forming a thick-walled cystic lesion.

**Figure 8:**
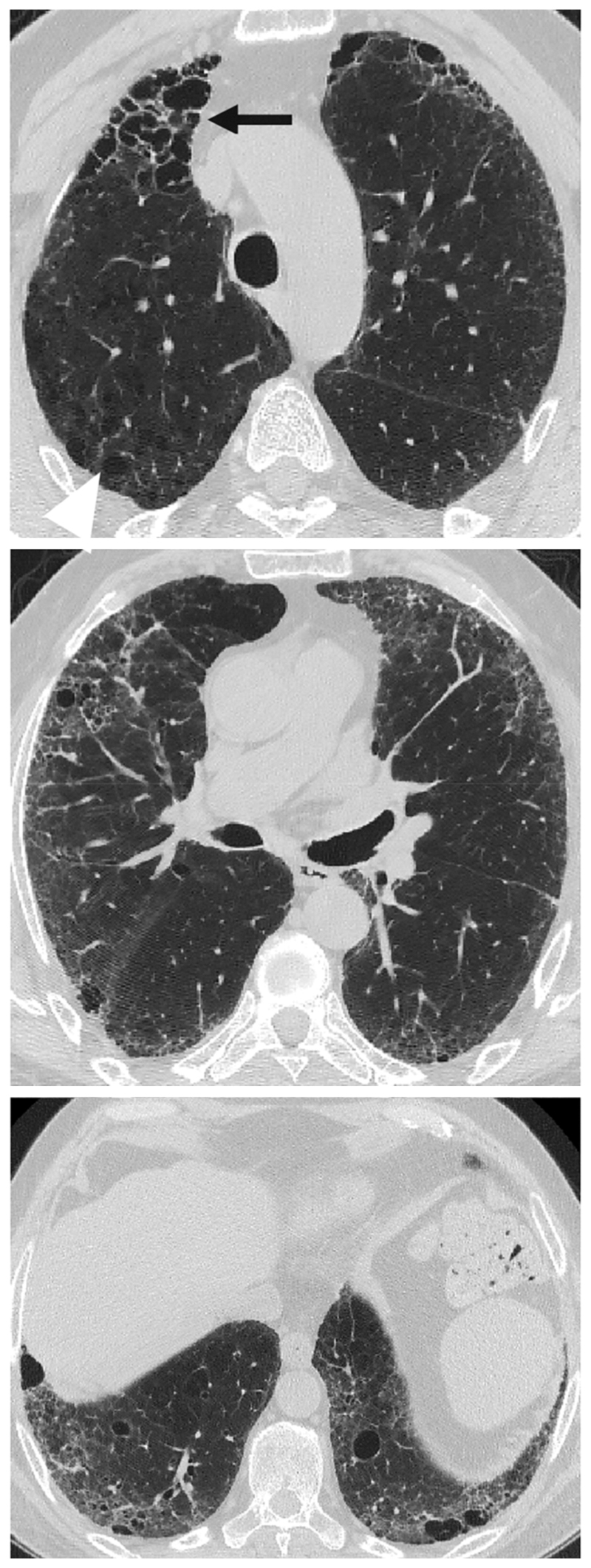
HRCT in a 69-year-old male with idiopathic pulmonary fibrosis, showing diffuse emphysema through the lung zones and lower zone predominant fibrosis (combined pulmonary fibrosis and emphysema, unclassifiable pattern). Emphysema in the right upper lobe is a combination of admixed (black arrow) and isolated emphysema (white arrowhead). Admixed emphysema is visible in the midzones, whilst in the lower zones a mixture of admixed and isolated paraseptal and centrilobular emphysema is apparent.

Emphysema is identified as a region of low attenuation (also termed density), not bounded by visible walls on CT ^139^. Emphysematous foci can be categorized as centrilobular, paraseptal, or panacinar ^140^. Interstitial fibrosis is identified as regions of increased parenchymal attenuation, appearing as reticulation and/or ground glass opacities, variably associated with honeycombing and/or traction bronchiectasis (Table 5). Patterns of emphysema on HRCT in CPFE have been tentatively classified into broad groups ^129,141–143^(Figures 1-8), however additional work is needed to better define CPFE morphologic subtypes. No studies have formally compared patterns of emphysema in CPFE versus COPD ^140^.

HRCT scanning parameters for appropriate assessment of ILD can be found elsewhere ^144^. Classical HRCT patterns may be altered when emphysema and fibrosis are spatially superimposed. For example, expansion of the interlobular septa with collagen fibrosis can make paraseptal emphysema appear as honeycomb cysts. Most studies have focused on patients with IPF and/or a UIP pattern on HRCT imaging ^1,24–26,29,30,32,33,38,55,56,58,61,120,121,137,138,141,145–153^, although others have included patients with a variety of ILD subtypes and imaging patterns. Given the high proportion of patients with CPFE with UIP pattern on HRCT (Table E3)^1,137,145–147^, distinguishing admixed emphysema from honeycomb cysts is challenging. The coexistence of emphysema and fibrosis can also create an imaging pattern of thick-walled cystic lesions ^141,142^, thought to reflect the expansion of emphysema as it is pulled apart by adjacent contracting fibrotic lung. This process, the committee suggests, could be termed traction emphysema given its putative mechanistic similarity to tractionally dilated bronchioles commonly seen within areas of fibrosis. Thick-walled cystic lesions predominating in basal posterior lung zones, consisting of large emphysematous areas surrounded by reticular opacities, have been more frequently described in CPFE than in isolated IPF ^141,142^. However, it is unknown whether thick-walled cystic lesions are specific for CPFE, and their evolution is yet to be fully described.

New imaging modalities may allow early diagnosis or distinguish IPF from CPFE ^154–156^. Imaging modalities that combine functional information and anatomic detail such as hyperpolarized Xenon MRI may advance the discrimination of superimposed emphysema and fibrosis ^157,158^. The reduced red blood cell spectroscopic peak in areas of fibrosis seen with hyperpolarized Xenon MRI could be evaluated alongside the increased apparent diffusion coefficient seen in areas of emphysema where disrupted acinar-airway integrity increases Brownian motion ^159,160^. However, more work is needed to understand whether aerated honeycomb cysts may mimic similar-sized emphysematous lesions on apparent diffusion coefficient.

All routinely used imaging modalities are constrained by the lack of histopathological definition of damage as emphysematous or fibrotic. Newer ex-vivo imaging techniques like hierarchical phase contrast tomography, able to image entire lungs and focal regions of interest at 2.5μm, may transform our understanding of emphysema-fibrosis interactions by essentially providing three-dimensional histopathological characterization of the lungs ^161^.

### Quantification of HRCT abnormalities

Disease quantification has predominantly relied on semi-quantitative visual HRCT estimation of emphysema and fibrosis extents. However, this approach is limited by several challenges: 1) interobserver variation ^30^; 2) time constraints for visual scoring; 3) varying methodologies for HRCT scan interrogation (e.g. evaluation of whole CT volumes versus interspaced images); 4) varying HRCT spatial resolution; 5) whether emphysema extent alone or both emphysema and fibrosis extents are quantified ^24,29,30,47,52,58,121,138,147,162^; and 6) variations in emphysema quantification e.g. total extent of emphysema, versus extents of emphysema lying either within or separate to areas of fibrosis^30,31,52,58,163^.

### Emphysema quantification

The emphysema component of CPFE has been evaluated by imaging rather than lung function tests, given the confounding impact of fibrosis on lung physiology. Reliable estimation of emphysema extent in patients with established pulmonary fibrosis poses significant challenges. Most studies use visual assessment of emphysema by an experienced radiologist, a method that is readily available and has moderate inter-rater agreement. Emphysema thresholds used to characterize a CPFE phenotype on imaging ^3^(see following section), include: >0% ^26,30,58^, >5% ^121^, >10% ^24,32,148^ and >15% ^162^ of total lung volume. One study limited assessment of emphysema extent to above the level of the carina ^51^.

Quantitative methods for scoring emphysema using computer-based measurement of lung density (e.g. density masking) are typically used in studies of chronic obstructive pulmonary disease and remove the problem of observer variability. However, this methodology of emphysema quantification is poorly suited to CPFE despite being attempted in some series ^36,42,58,122,123,148,163–165^, because it fails to discriminate between low density areas due to emphysema and low density due to honeycomb cysts, traction bronchiectasis or non-emphysematous mosaic attenuation due to small airways disease. Until this limitation can be overcome (possibly by artificial intelligence), visual quantification of emphysema extent remains the method of choice in CPFE.

Differences in morphological patterns of emphysema (subtype: paraseptal vs centrilobular vs mixed vs indeterminate; predominant distribution in the axial plane) have also been used to describe CPFE subtypes ^26,120,129,147^. Large multicentered studies are required to determine whether these morphological CPFE subtypes correlate with distinct functional or prognostic disease groups. The subtypes identified on HRCT imaging could also be confirmed using histopathological correlative studies ^151^.

### Interstitial lung disease quantification

A minimal threshold extent of lung fibrosis on HRCT imaging has rarely been used in CPFE despite the clinical importance of fILD severity (Figure E3). The concept of a minimal threshold of fibrosis to define CPFE is particular relevant to lung cancer screening populations. Participants in screening studies are typically older with a heavy smoking history, and both emphysema and interstitial lung abnormalities (ILAs) will be frequent ^127,166–169^. This may result in a high prevalence of combined ILAs ^170^ and emphysema in screening populations.

In the context of IPF, where ground glass opacification on HRCT largely represents fine fibrosis, fibrosis extent in CPFE has been calculated by summing ground glass opacities, reticulation, and honeycomb cysts ^118,171^. However, quantitation of fibrosis extent is confounded by volume loss, with lower lobes sometimes greatly contracted to apparently small areas of fibrosis. Yet when considering CTD-related ILD or fHP where ground glass opacities may reflect inflammation rather than fibrosis, there is no consensus on whether ground glass opacities should be considered as part of CPFE fibrosis extent. It has been suggested that to conform to the “fibrosis” element required by a definition of CPFE, ground glass opacities should be quantified only if overlaid by reticular lines or traction bronchiectasis (Figures E4 and 9)^171^. Agreement on ILD patterns to be quantified in CPFE will be important to harmonize study interpretation in the future, as well as agreement on preferred visual fibrosis quantification methodologies (volumetric lobar scores versus 5- or 6-level HRCT slice scoring; categorical versus continuous scales of fibrosis extent).

## Pathology features

CPFE was originally defined based on clinical, physiologic, and HRCT features ^2^. Histopathologic studies of patients with severe CPFE defined in this way are limited to small series of autopsy cases or explants given the risk of surgical lung biopsy in this population ^141,149,151^. Overlapping patterns of smoking-related abnormalities are common in lung biopsies from patients undergoing elective lung biopsy for fILD including some for whom a diagnosis of CPFE is uncertain or unanticipated. Here we review patterns of smoking-related abnormalities and pulmonary fibrosis with a focus on features characteristic of CPFE.

### Histopathological patterns of smoking-related abnormalities and fibrosis in CPFE

Emphysema is required for a diagnosis of CPFE and is defined as abnormal, permanent enlargement of airspaces distal to the terminal bronchiole, accompanied by the destruction of their walls, without obvious fibrosis (Figure 10)^172–175^. Morphologic studies of carefully inflated lung specimens from explant pneumonectomies and autopsy lungs provided the basis for an anatomical definition of emphysema and continue to inform our understanding of its pathogenesis. However, the coexistence of emphysema and patterns of fILD can be seen in biopsies, a situation in which pathologists need to document the emphysema as well as the fILD. Centrilobular emphysema is an upper lobe predominant form of emphysema caused by cigarette smoking that is often accompanied by paraseptal emphysema in CPFE patients. Emphysema is common in surgical lung specimens and frequently coexists with other smoking-related abnormalities including respiratory bronchiolitis (RB) and SRIF ^5,176^.

RB occurs almost exclusively in cigarette smokers and is defined by the presence of pigmented alveolar macrophages clustered within the lumens of respiratory bronchioles and peribronchiolar air spaces without significant inflammation or fibrosis (Figure 11)^177^. RB is a common incidental finding in surgical lung specimens, including biopsies in which it may accompany any pattern of pulmonary fibrosis including especially SRIF, desquamative interstitial pneumonia (DIP), UIP and Langerhans cell histiocytosis given the high prevalence of smoking in these populations. RB-ILD is a diagnosis of exclusion reserved for patients in whom RB is thought to explain diffuse ILD after elimination of diagnostic alternatives, a circumstance histologically indistinguishable from incidental RB. RB by itself is not a fibrotic lesion and therefore an insufficient explanation for fibrosis in patients suspected of having CPFE.

Patterns of fibrosis observed in patients with CPFE are histologically heterogeneous (Table 6)^178,179^. These patterns include a distinctive form of fibrosis linked to cigarette smoking for which Katzenstein proposed the term SRIF ^5,23,61,151^. SRIF overlaps with previous descriptions of AEF ^4,180,181^, RB-associated ILD with fibrosis ^22^, RB with fibrosis ^182^, and DIP^5^. Some cases with a pattern of fibrotic NSIP may also be related to smoking. SRIF is characterized by densely eosinophilic collagen deposited in expanded alveolar septa with preservation of lung architecture and little or no inflammation (Figure 12). SRIF has a distinct predilection for peripheral subpleural and peribronchiolar parenchyma without the variegated “patchwork” distribution more characteristic of UIP. When combined with paraseptal emphysema, SRIF may account for the “thick-walled cystic lesions” that are unique to CPFE and distinct from the honeycomb cysts of UIP (Figure 13)^61,141,142^. Like RB, SRIF is a common incidental finding in surgical lung specimens, including lung biopsies from patients with other patterns of pulmonary fibrosis ^23^. Isolated SRIF represents the primary pathological abnormality in a subset of patients with clinical features of ILD in whom it is often combined with RB (Figures E5 and E6) ^22,182^. SRIF without other patterns of concomitant fibrosis has not been established as a cause of CPFE; therefore, attributing pulmonary fibrosis to SRIF in patients with CPFE requires exclusion of other fibrotic patterns including most importantly UIP.

Langerhans cell histiocytosis (LCH) is a potentially fibrotic form of smoking-related ILD that may occur in combination with other smoking-related abnormalities including emphysema, RB, SRIF, and DIP ^5,184^. Advanced disease is characterized by cystic change on HRCT that may be difficult to distinguish from emphysema ^185^, and a pattern of fibrosis in surgical specimens that may mimic other forms of fILD. Histopathological examination of surgical specimens from patients with advanced LCH, whether explants or diagnostic biopsies, is often complicated by the absence of diagnostic Langerhans cells. Microscopic features helpful in separating LCH from other patterns of fibrosis include stellate bronchiolocentric nodules and a characteristic pattern of affiliated paracicatricial airspace enlargement (“scar emphysema”) without subpleural honeycomb change (Figure E7).

There are no criteria for establishing a diagnosis of CPFE on the basis of histopathological findings alone. Supportive features include a combination of emphysema and a pattern of fibrosis other than SRIF or LCH (Figure 12). UIP is the most commonly reported pattern of pulmonary fibrosis in patients with CPFE (Figures E8 and E9)^1,37,141,151,186,187^. Identifying UIP typical of IPF in the setting of emphysema requires recognition of patchy fibrosis, fibroblast foci, and honeycombing without histologic features to suggest an alternative such as LCH, f-HP, or CTD-associated UIP ^48,144,179^. Unique to UIP in CPFE is the presence of thick-walled cysts resulting from the combination of emphysema and SRIF (figure 13). Other less commonly described patterns of fibrosis include fibrotic NSIP and DIP ^1,188,189^. Classifying subtypes of pulmonary fibrosis may be challenging and therefore the histopathological features may remain indeterminate for UIP in the setting of concomitant emphysema ^1,151^.

### Comorbidities identified on basis of histopathological features

The dismal prognosis of CPFE may result from vascular changes that correlate with PH. In a comparison of autopsy findings in patients with CPFE, IPF, and emphysema alone, vascular changes were more extensive in CPFE and IPF compared to those with emphysema alone ^149^. Vasculopathy was limited to areas of emphysema in those with emphysema alone, but involved emphysematous, fibrotic, and relatively preserved parenchyma in CPFE and IPF. Vascular changes included intimal thickening and medial hypertrophy in small muscular pulmonary arteries as well as intimal thickening in comparably sized small veins. Plexiform lesions were rare and seen only in a small minority of CPFE and IPF patients.

Malignancy is also not uncommon in CPFE, with a higher prevalence of squamous cell carcinomas amongst surgically resected cases ^38^.

## Outcome and complications

There are several important outcomes that have specific relevance in patients with CPFE, with lung cancer and PH being the most clinically relevant. It is currently unknown whether the risk of complications may differ according to different patterns of CPFE (Table 5, Table 6).

### Pulmonary hypertension

PH has been reported in 15-55% of patients with CPFE ^1,32,49,50,137^, with some studies suggesting an increased prevalence in a variety of ILDs ^49,120^ and others not confirming this association ^56,60^. Discrepant estimates of PH prevalence may be due to differing methods of PH assessment (e.g. echocardiographic vs. right heart catheterization-defined PH) and differences in statistical modeling ^190^, and could also be attributable to differing severity of fibrosis and emphysema on HRCT ^30^. Pathophysiology of PH in CPFE is probably multifactorial ^191^. Some studies have suggested that the severity of PH is worse among those with CPFE compared to both IPF ^24,32^ and COPD ^192^ or emphysema ^149^ alone. Estimated systolic pulmonary artery pressures are higher in patients with CPFE than in those with isolated IPF ^24,119^. The additional burden of emphysema, over and above a given extent of fibrosis, increases the risk of PH. However, the likelihood of PH does not differ for matched extents of disease (combined fibrosis and emphysema) on HRCT (or when adjusted for DLco) between patients with CPFE and those with fibrosis alone ^30,58^.

### Lung cancer

Lung cancer has been reported in 2-52% of patients with CPFE ^35,37,41,42,57,58,60,137,148,162,163,193^, with varying methodology (cross-sectional, longitudinal follow-up). In a meta-analysis ^194^, patients with CPFE (UIP and emphysema) had a higher risk of lung cancer than those with IPF alone (OR 2.69; 95% CI: 1.78-4.05)^194^. There were similarly increased risks of lung cancer in patients with CPFE and UIP with the presence of any amount of emphysema (OR 2.93; 95% CI: 1.79-4.79) and with emphysema in ≥10% of the lung volume (OR 2.22; 95% CI: 1.06-4.68), compared to patients who had UIP without emphysema ^194^.

The most common histopathologic subtypes of lung cancer in CPFE are squamous cell carcinoma and adenocarcinoma ^38–40,44,45,116,162,193,195–198^. In contrast to the general epidemiology of non-small cell lung cancer with adenocarcinoma accounting for 50% of cases ^199^, squamous cell carcinomas appear to be more frequent in patients with CPFE ^39,40,44,45,162,193,195–198,200^. The majority of the lung cancers were located in the lower lobes ^39,195^. There is greater invasion and the diagnosis is made at a later stage compared to non-small cell lung cancer without CPFE ^198,201^.

Although individual studies differ in their conclusions ^38,44,45,57,145,195,200^, a systematic review and meta-analysis concluded that the presence of CPFE is associated with worse survival in patients with non-small cell lung cancer^198^. ^38,44,57,145,195,200^Among patients with CPFE and lung cancer, the presence of honeycombing, later cancer stage, and reduced feasibility of surgical resection are predictors of mortality ^202^. The poor outcome is at least in part related to increased morbidity and mortality of cancer treatments in CPFE, which often limits standard therapy ^40,43,45,196,197,200,203,204^.

### Acute exacerbation

Acute exacerbations of IPF have been reported in patients with CPFE with varying prevalence ^50,137,141,195,205–207^. Risk factors for acute exacerbation in CPFE may be similar to IPF, including worse gender-age-physiology score and the presence of lung cancer, particularly following surgical resection ^43,195,196,204,205^. Diffuse ground glass and/or consolidation on chest HRCT help to differentiate exacerbations of fibrosis from exacerbations of emphysema in CPFE ^208^. The prognosis of acute exacerbation in CPFE might be better than that of isolated IPF ^31,207^.

### Mortality

CPFE is associated with poor survival, with different estimates between series ^1,32,35,37,38,55,115,137,163,209^, which probably reflect differences in sample size, follow-up time, and comorbidities. Patients with CPFE have worse survival than patients with emphysema alone on HRCT ^42^. As compared to patients with IPF alone, patients with CPFE were reported to have worse ^32,38,49,55,56^, comparable ^24,26,35,37,39,47,57–59,119,210–212^, or better survival ^31,120,147^. Possible explanations for this discrepancy include diagnostic contamination (with a higher proportion of non-IPF cases in CPFE populations with better survival), attrition bias ^26,60^, differences in the relative extent of emphysema versus fibrosis in different cohorts ^24,59,213^, and a ‘healthy smoker’ effect ^214^. A positive correlation was found in some series between the extent of emphysema and the extent of fibrosis ^32^, however a negative correlation was found in others ^29,129,215^. In some series, an attempt was made to examine CPFE specifically in sub-groups mostly or wholly made up of IPF ^24,32,36,42,55,120,146,212^. However, this goal is complicated by the lack of histologic confirmation of UIP in most CPFE patients with suspected IPF, and difficulties discriminating between true honeycomb change (required for an HRCT pattern of UIP) and the admixture of emphysema and pulmonary fibrosis (“pseudohoneycomb change”) on HRCT ^216,217^.

Prognostic evaluation of CPFE, with particular reference to comparisons between CPFE and isolated IPF, requires quantification of both pulmonary fibrosis and emphysema. This was conducted in two retrospective cohorts of patients with IPF ^58,212^, using both visual analysis to the nearest 5% ^58,212^ and computer-based analysis with the CALIPER software ^58^. The global disease extent on HRCT (i.e. the combined extent of fibrosis and of emphysema) and the baseline DLco both predicted mortality, reflecting the overall severity of parenchymal lung destruction ^58,212^. After correction for baseline severity using DLco, the presence or extent of emphysema did not impact on survival ^58^. ^32,212,214^

There is no evidence that disease progression, FVC trends apart, differs between patients with IPF who have and do not have emphysema ^26,29,60,164^. It is likely that the lower rate of FVC decline in CPFE ^29^ is related to the preservation of volumes by emphysema, especially when admixed with fibrosis ^58^, rather than to slower progression of fibrosis. Further studies should compare progression of fibrosis using serial HRCT, DLco, CPI, and symptom assessment in patients with or without emphysema.

Predictors of mortality in patients with CPFE include DLco ^27,60,115^, CPI ^119,187^, age ^27^, and the presence of specific co-morbidities such as PH ^1,27,32,49,115^ and lung cancer ^41,57,141,218^(Table 7). FVC has not been shown to be a predictor of death among patients with CPFE, unless the FVC is <50% predicted ^32^ and nor is the smoking history ^163^. However, predictors of death in CPFE, including the impact of PH ^30^, are not identified consistently in all studies and further work is needed to determine risk factors for death among patients with CPFE syndrome.

### Outcomes in summary

Overall, the data suggest that outcomes are worse for a given extent of fibrosis, when there is emphysema in addition to fibrosis (e.g. outcomes are worse in a patient with 10% fibrosis extent and 20% emphysema extent than in a patient with 10% fibrosis extent and no emphysema). However, the risk of mortality and of developing PH does not differ in patients with both IPF and emphysema compared to those with fibrosis alone when adjusting for severity using baseline DLco or total disease extent on HRCT (e.g. total extent of fibrosis and emphysema)(e.g. outcomes are comparable in a patient with 20% emphysema extent and 10% fibrosis extent, and in a patient with 15% emphysema extent and 15% fibrosis extent)^30,58,190^.

## Pathogenesis and putative mechanisms

The pathogenetic mechanisms leading to the coexistence of emphysema with IPF and other fILDs remain unclear. Likewise, it is uncertain whether IPF and non-IPF/ILD are causally linked with emphysema or if they represent different lung disorders running in parallel and sharing some mechanisms.

Clustering of pulmonary fibrosis and emphysema, i.e. increased risk of emphysema in patients with various fILDs ^28,49,51,222^, supports the notion of a shared pathophysiology. There is bidirectional interaction between emphysema and fibrosis through mechanical forces ^52,223–225^. Many pathways and pathogenetic mechanisms are shared between fibrosis and emphysema, including gene expression and pathways, gene variants, telomere dysfunction and shortening, alveolar alterations, epigenomic reprogramming, and enzymatic activity, especially matrix metalloproteinases (Table 8)(detailed description in online supplement). Both emphysema and fibrosis develop in several animal models ^18,20,99^. However, distinct gene variants and pathways were also identified between emphysema and fibrosis ^226–229^.

## Terminology and definitions

### Review of existing terminology and definitions

The contemporary terminology and definition of CPFE was provided in a 2005 publication that described a total of 61 patients who were retrospectively selected from a French multicentric study ^1^. In this publication, CPFE was described as the presence of upper zone predominant emphysema on HRCT plus a peripheral and basal predominant diffuse parenchymal lung disease with significant fibrosis. Emphysema was not quantified, however “conspicuous” emphysema at visual HRCT inspection was an inclusion criterion. A large number of subsequent studies on CPFE have used similar terminology, but with varying definitions and diagnostic criteria. Despite this somewhat imprecise definition, such criteria identified patient populations with comparable physiology in several studies ^1,50,115^.

A recent systematic review identified the heterogeneous definitions and diagnostic criteria previously used in 72 previous studies on CPFE ^3,279^. This systematic review was updated in December 2021 and includes 96 studies, which are summarized in Table 9. CPFE was diagnosed based on criteria proposed by Cottin et al. ^1^ in 53% (51/96) of all eligible studies ^3^(Figure E10). A diagnosis of IPF was required in 47 studies (49%), while 49 (51%) included a variety of non-IPF fILD. The extent of fibrosis was determined visually in 89 studies (93%). A minimal extent of 10% fibrosis on chest HRCT was required in three studies ^202^. The majority of studies (75%) diagnosed CPFE if there was any emphysema present on chest HRCT, while 25% used a specific threshold: >5% ^121,124^, >10% ^24,32,148,202,206^, >15% ^162^, >20% ^145^, and >25% ^151^ of total lung volume. Quantitative HRCT was used to evaluate fibrosis extent in 7 studies (e.g., percentage of voxels with mean lung attenuation between 0 and -700 Hounsfield units). Fifty two studies required that emphysema be upper lung predominant, 10 studies included emphysema in all locations, and 34 studies did not specify location criteria. The extent of emphysema was assessed visually in 85/96 studies, with 4 studies using the Goddard method of quantifying emphysema ^119,207,211,280^, and the remaining 11 studies using quantitative HRCT (e.g., percentage of voxels with mean lung attenuation less than -950 Hounsfield units). Few studies used values from pulmonary function tests to define CPFE.

### Limitations of previous definitions and terminology of CPFE

Research in CPFE has primarily been driven by observational studies that have led to an appreciation that CPFE possesses unique clinical, radiologic, and physiologic features. However, a major limitation of previous CPFE research is the heterogeneity of study populations and criteria used to define CPFE, prohibiting direct comparison of different cohorts and validation of key findings.

Both imaging and histopathologic studies indicate that CPFE can encompass a variety of fILDs. IPF and COPD share common risk factors of older age and a history of smoking, resulting in this definition likely capturing the largest and most clinically relevant subgroup of patients with ILD who have concurrent emphysema, while also ensuring a relatively homogeneous population. Allowing CPFE to include a variety of ILD subtypes has the advantage of capturing all patients with these two diseases; however, this approach results in a heterogeneous population that complicates assessment of disease biology that might vary across ILD subtypes. An inclusive definition that encompasses all ILD subtypes can also introduce bias in comparison to control populations given the common risk factors for emphysema (e.g. older age and a history of smoking) that also predispose to some ILD subtypes (most notably IPF). Given its association with smoking, IPF is more frequently associated with emphysema than are CTD-ILD or fHP, even if there is now acceptance that smoking can also cause fibrosis distinct from IPF ^242–245^. A potential approach to reconcile these conflicting priorities is to carefully and transparently define CPFE in a manner that reflects the clinical setting and/or research objectives. For example, studies evaluating prognosis are likely to require separation of IPF and non-IPF patients, while studies evaluating lung physiology may not require such stratification.

Furthermore, few studies have quantified the extent of fibrosis and of emphysema on chest HRCT. Automated quantification is challenging when both components are present (see section on imaging), hampering the development of imaging criteria and consistency between studies. Hence, the term CPFE does not specify extent thresholds for either pulmonary fibrosis or emphysema, with some previous studies including patients with any amount of each abnormality, and other studies setting higher thresholds based on supposed clinical relevance. When used, specific extent thresholds are more commonly applied to emphysema than to pulmonary fibrosis. It is also debated as to whether disease extent should be quantified by visual or quantitative methods. The designation of CPFE only if certain thresholds for emphysema and/or fibrosis are exceeded has the advantage of excluding subclinical disease that may be of no or minimal clinical consequence, and selecting subjects who are at risk of outcomes typical of CPFE. Using such thresholds increases specificity for CPFE, but at the expense of excluding patients with lesser extent of either component. Decisions regarding the use of specific thresholds have, thus, been partially driven by the purposes of individual studies, with biological studies on disease mechanisms potentially not needing high severity thresholds, but such thresholds viewed as more appropriate for clinical or physiological studies in which trivial disease is unlikely to have a meaningful impact. Studying patients with early disease (e.g., with ILAs)^127^ offers the best opportunity to learn more about the natural history of CPFE and important biological processes that underlie both of these diseases. Future definitions and diagnostic criteria should allow for identification and study of these patients with early disease, particularly when studying biological mechanisms of disease.

### Proposed terminology and definitions

The lack of diagnostic criteria and inability to directly compare study populations has hampered the study of the biology, management, and prognosis of CPFE. There is a need to establish specific criteria for CPFE, including standardized and reproducible methods of quantifying both emphysema and fibrosis. The committee proposes a common terminology (Table 10), a provisional, broad *research definition* for CPFE that will enable future research, and provisional classification criteria of CPFE *clinical syndrome* intended to serve clinicians managing patients with CPFE (Table 11). Therefore, the CPFE *clinical syndrome* was identified based on clinical utility (see below), whilst the *research definition* of CPFE delineates a larger group of patients that should continue to be studied with the ultimate goal of reviewing the syndrome threshold as further clinical and pathogenetic data emerge.

The committee acknowledged the absence of clear justification to deviate from the entrenched historical term of “CPFE”, recognizing that this is the simplest and broadest label for this group of patients. Similarly, the committee proposes retention of the literal definition of CPFE as the coexistence of both pulmonary fibrosis and emphysema. All subtypes of fILD and emphysema are thus included in the overall CPFE population, but with an important requirement that the fILD subtype be clearly described given the potential biases that can be introduced by including multiple ILDs in this definition. However, it was proposed that studies on CPFE include a comprehensive description of the radiologic and, when available, of the pathological patterns (e.g. “CPFE – IPF”, or “CPFE – radiologic UIP”, or “CPFE - histologic nonspecific interstitial pneumonia”, or “CPFE - radiologic SRIF”) or the underlying disease when known (e.g. CPFE – fHP or CPFE – RA). This would facilitate comparison between studies through a common terminology, and emphasize the heterogeneity of what can be grouped under the umbrella of CPFE.

The most common definition of CPFE is the presence of lung fibrosis and upper-lobe predominant emphysema. The requirement for emphysema in many studies to be upper-lobe predominant minimizes potential confounding by the presence of honeycombing, which is typically lower-lobe predominant and can be difficult to distinguish from paraseptal emphysema. However, the committee proposed that in CPFE, emphysema may be present in other areas of the lung, may be admixed with fibrosis, or may be replaced by thick-walled large cysts greater than 2.5-cm in diameter (“CPFE, thick-walled large cysts variant”).

**Figure 9:**
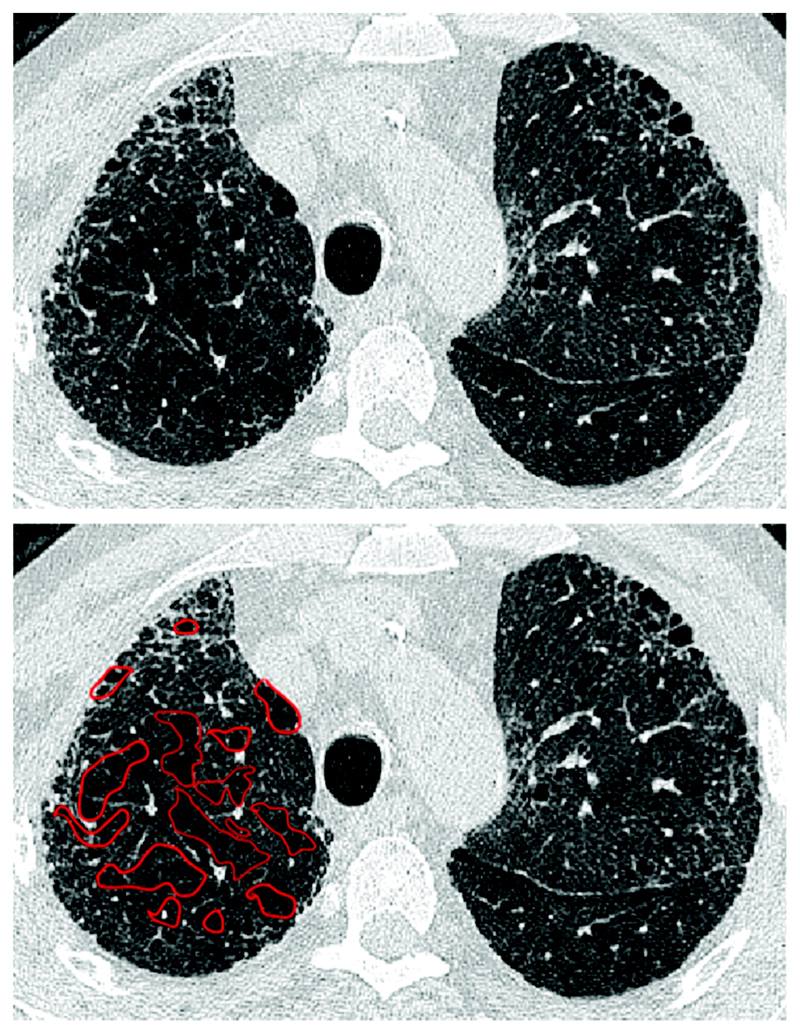
Visual scoring of emphysema. Axial section through the upper lobes in a patient with idiopathic pulmonary fibrosis and emphysema (top). Emphysema is distributed irregularly through the lobe making visual quantification difficult. Visually combining the emphysematous foci together (bottom) and estimating the fraction of the lobe that it comprises (i.e. 50%, 33%, 25%, 20%, 15%, 10%, 5%) can simplify quantitation in challenging cases (online supplement).

**Figure 10:**
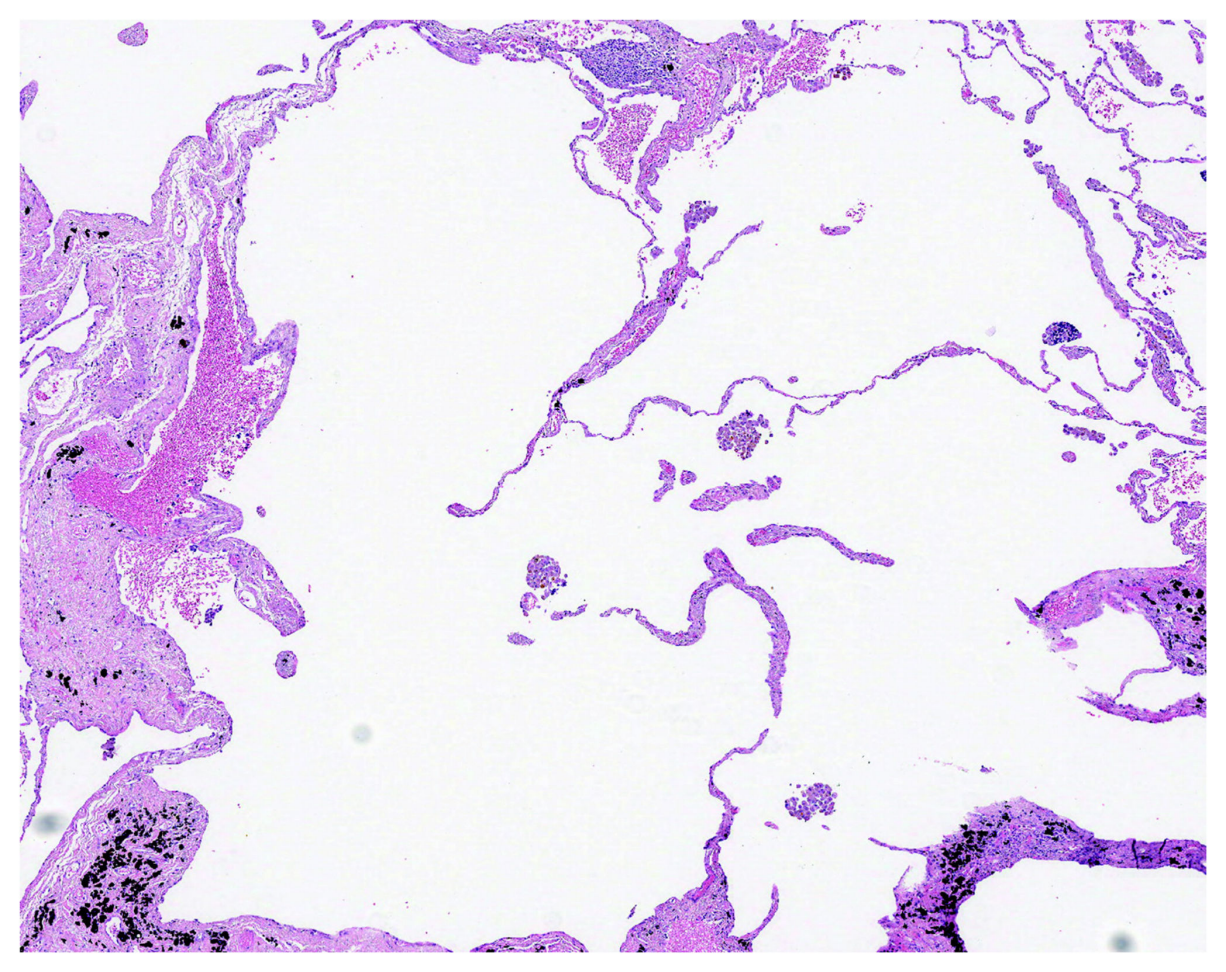
Centrilobular emphysema in wedge excision in a heavy smoker with a peripheral small cell carcinoma. An intermediate magnification photomicrograph shows enlarged airspaces with destruction of bronchiolar walls evidenced by detached free-floating connective tissue fragments. Hematoxylin and eosin staining.

**Figure 11:**
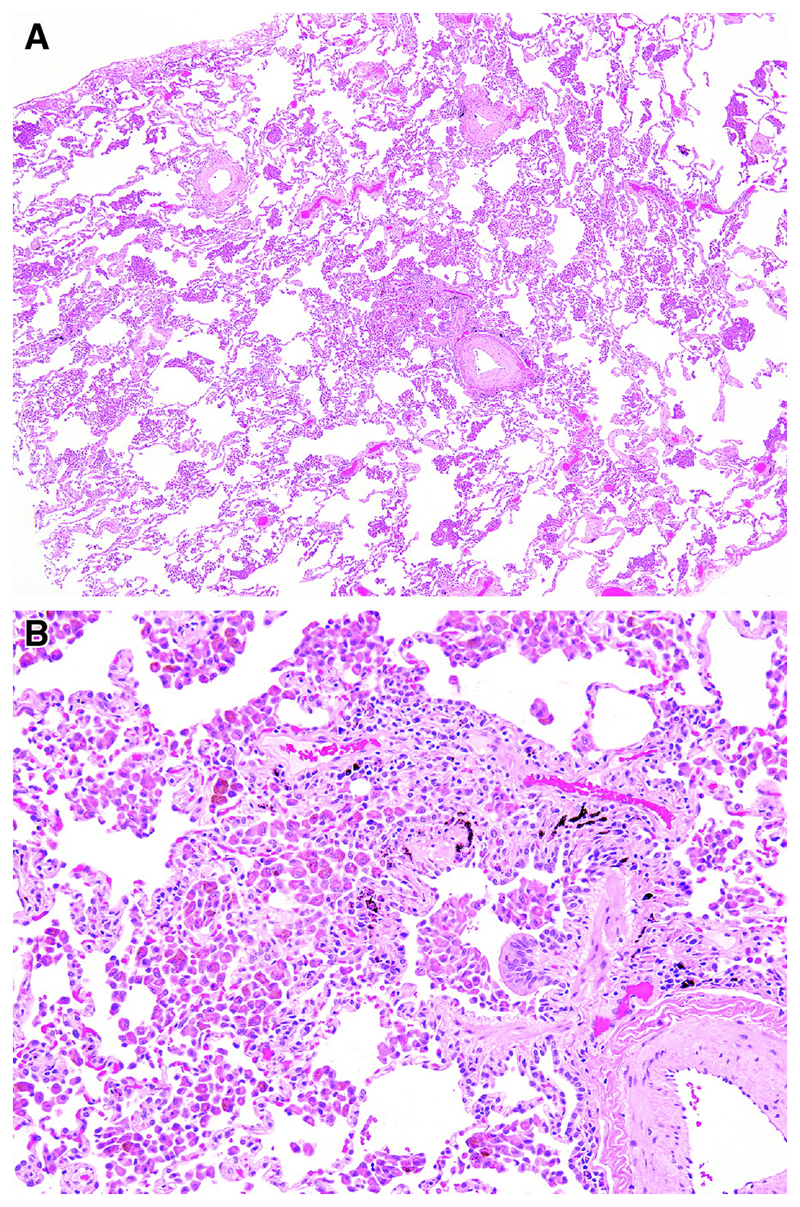
Respiratory bronchiolitis (RB) in a patient with RB-ILD. RB is a common finding in patients with CPFE who have concomitant pulmonary fibrosis. A. Low magnification photomicrograph showing RB characterized by clusters of lightly pigmented macrophages in the lumens of distal bronchioles and peribronchiolar air spaces. B. Higher magnification view showing pigmented intraluminal macrophages in respiratory bronchiole and surrounding air spaces with no significant inflammation or fibrosis. Hematoxylin and eosin staining.

**Figure 12:**
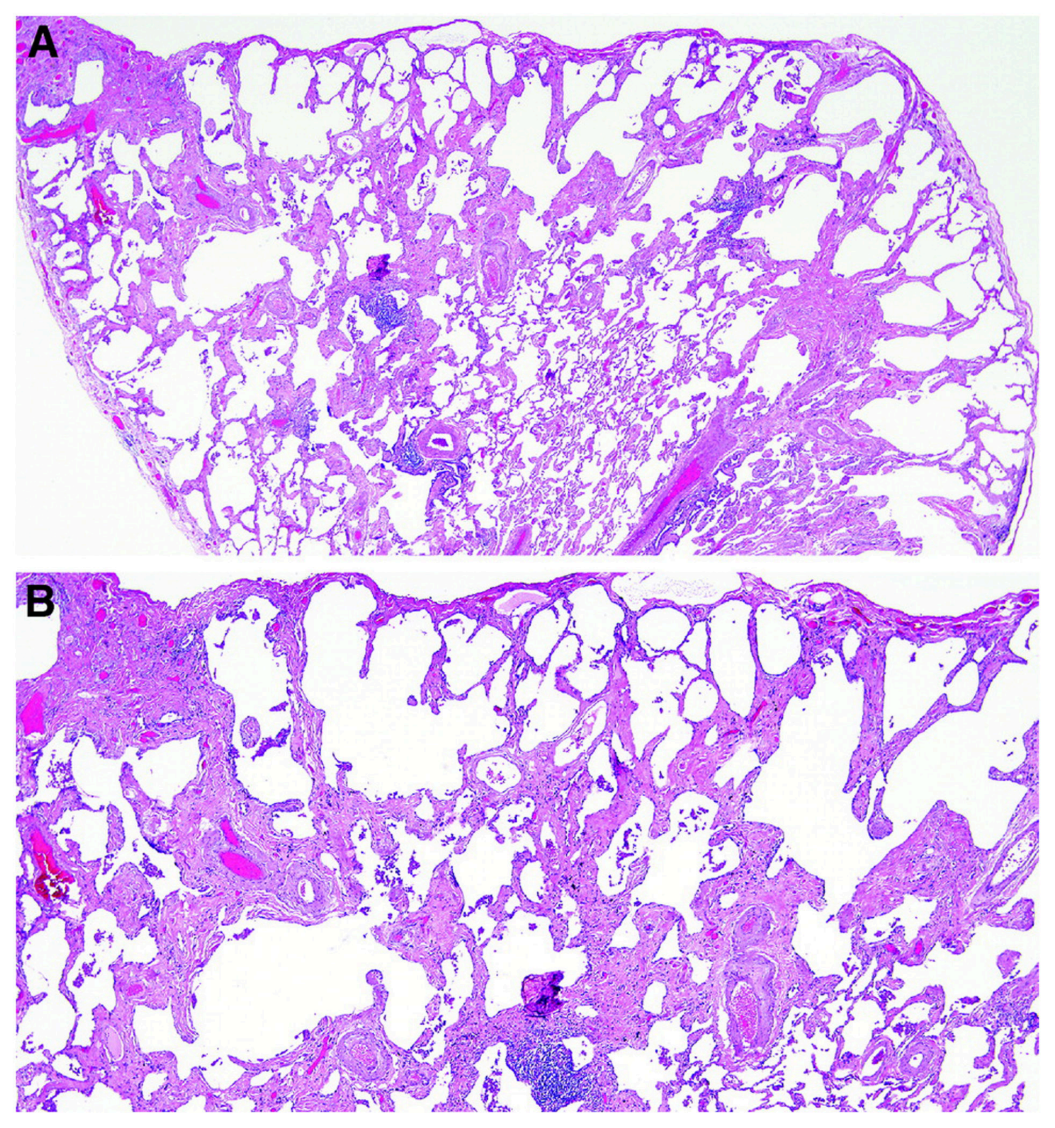
Smoking-related interstitial fibrosis (SRIF) in upper lobe biopsy from 120-pack-year smoker with CPFE characterized by a combination of emphysema and usual interstitial pneumonia in middle and lower lobe biopsies. A. Low magnification photomicrograph showing mild expansion of subpleural parenchyma by paucicellular, densely eosinophilic (“amyloid-like”) collagen with preservation of lung architecture. B. Higher magnification photomicrograph showing subpleural fibrosis without honeycomb change or fibroblast foci. There is mild associated emphysema.

**Figure 13:**
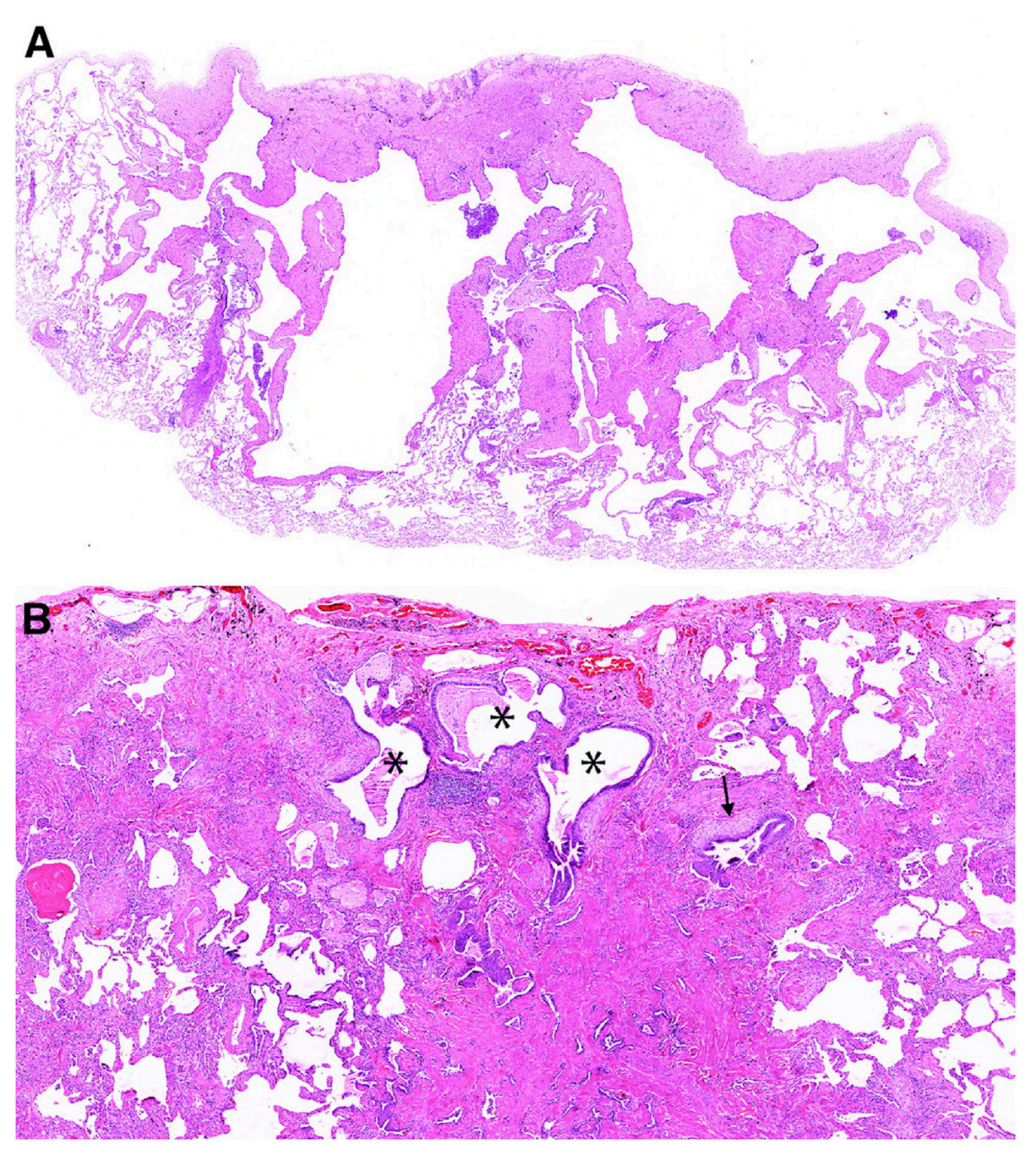
Subpleural cysts of smoking-related interstitial fibrosis (SRIF) contrasted with honeycomb change in usual interstitial pneumonia (UIP). A. Low magnification photomicrograph of SRIF with associated distal acinar (“paraseptal”) emphysema forming thick-walled cysts. Overall lung architecture is preserved and the paucicellular fibrosis lacks the qualitative variability more characteristic of UIP. The cystic spaces are mainly lined by attenuated pneumocytes. B. Low magnification photomicrograph of honeycomb change in UIP. The fibrosis has a patchy distribution with collapse and distortion of normal lung architecture. The fibrosis includes fibroblast foci (arrow), and a mild, patchy infiltrate of lymphocytes resulting in a variegated appearance that contrasts with the uniform, paucicelluar, densely eosinophilic fibrosis in SRIF. Cystic honeycomb spaces (*) are mainly lined by columnar bronchiolar type epithelium rather than pneumocytes. Hematoxylin and eosin stain.

Some studies have required a specific extent of emphysema, with 15% predicting a distinct outcome for patients with more than this threshold ^29^, and 10% being a more commonly used threshold ^24,32,124,148^. For research purposes, the committee proposed to define CPFE based on emphysema extent ≥ 5% of total lung volume (Table 11, Figure 9, and online supplement). For clinicians managing patients with CPFE, the committee proposed classification of CPFE *clinical syndrome* based on emphysema extent ≥ 15% of total lung volume, and/or in cases of disproportionately decreased DLco or precapillary PH not related to the sole presence of emphysema, fibrosis, or etiological context. The committee acknowledged that further research is needed to refine criteria of CPFE *clinical syndrome*. For example, studies aiming to evaluate the clinical or functional outcome of patients with CPFE *clinical syndrome* should assess what specific extents of both pulmonary fibrosis and emphysema ensure clinical relevance of each component. Despite physiological differences compared to isolated ILD and COPD, lung function and especially FEV1/FVC is not sufficiently sensitive or specific to be useful in defining CPFE ^29^; more studies are needed to assess the potential value of Kco or FVC/DLco.

The committee did not recommend a minimal extent of fibrosis on HRCT, however acknowledged that fILD (not ILAs) is required to defined CPFE. The committee, however, recommended that fibrosis extent and emphysema extent should both be assessed in future studies, using visual assessment, and that the association of the study end points with the presence of emphysema above and below thresholds of emphysema extent should be analyzed, as well as their association with patterns of fILD. The committee also emphasized that there should be generally no restriction on the cause of emphysema (e.g., smoking cigarettes, cannabis, biomass fuel exposure) or of fILD (smoking cigarettes, CTD, idiopathic, etc) unless a study is focused on emphysema of a particular etiology. Future research is required to determine the reproducibility and relevance for research of the CPFE *research definition*; and the clinical utility of the classification criteria of CPFE *clinical syndrome*, which in the future may be refined based on physiologic or imaging predictors of outcome that are yet to be identified.

## Is CPFE a syndrome?

### Background and hypothesis

Current management and future study of CPFE will be facilitated by a clearer understanding of whether this entity has clinical relevance (clinical utility) or if it is biologically unique (pathogenic utility). In early descriptions ^12,17,19^, CPFE had been viewed as the coincidental coexistence of IPF and emphysema, with a common linkage to smoking. In 2005, the description of the characteristic functional profile of CPFE in a series of 61 patients ^1^, taken together with the observation of a high prevalence of PH, provided support for “the individualization of CPFE as a discrete clinical entity apart from both IPF and pulmonary emphysema”. The authors considered that “CPFE was not just a distinct phenotype of IPF, but deserved the terminology of syndrome as a result of the association of symptoms and clinical manifestations, each with a probability of being present increased by the presence of the other” ^2^. However, no consensus exists on whether CPFE is a syndrome or distinct entity.

The committee considered the following options for CPFE: 1) coexistence of two diseases with no clinically relevant implications or major pathogenetic significance (*two coincident diseases*); 2) coexistence of two diseases with clinically relevant implications and/or major pathogenetic significance (a *syndrome*); 3) a single biologically unique entity distinct from both IPF and emphysema (*one distinct disease*).

### Definition of a syndrome

In a seminal article ^6^, Dr. Scadding described a clinical syndrome as one of the four main classes of characteristics by which diseases could be defined : "Patients with a recognizably similar pattern of symptoms and signs were said to be suffering from the same disease. A recognizable pattern of this sort is called a syndrome" ^6^. A syndrome, therefore, consists of a disease or disorder that involves a particular group of signs and/or symptoms. However, the contemporary definition of a syndrome requires greater provenance than the mere recognition of an association, be it between clinical variables or underlying disease processes. A proposed syndrome generally provides either clinical utility (e.g. serves as an aid to diagnosis, prognostic evaluation, or management) and/or pathogenetic utility (e.g. underlying pathogenetic mechanisms unique to the syndrome are present, providing an avenue for the development of new therapies). In 2005, Cottin et al proposed CPFE as a discrete entity, arguing that “it deserves the terminology of syndrome as a result of the association of symptoms and clinical manifestations, each with a probability of being present increased by the presence of the other” ^2^.

The main arguments in favor and against CPFE being a syndrome are summarized in Table 12. The Committee favored the term of syndrome based on the following arguments:

### Pathogenetic utility

There are multiple pathways common to both pulmonary fibrosis and emphysema; however, no primary pathogenetic pathways unique to CPFE have been identified. One argument in favor of CPFE being a syndrome is the clustering of pulmonary fibrosis and emphysema, e.g. that the presence of emphysema on HRCT is more prevalent than expected in several fILDs (see section on pathogenesis). Taken together, these observations suggest that CPFE may result from involvement of shared pathways in at least some patients.

However, if CPFE represents a biologically distinct syndrome, it is questionable whether it will be applicable to all patients with CPFE. Despite the phenomenon of clustering of emphysema with pulmonary fibrosis, the two diseases will inevitably co-exist in some patients as coincidental smoking-related processes. The definition of a patient group with a unique pathogenetic pathway, if it exists, is likely to require careful morphologic evaluation of histopathologic and HRCT features. Thick-walled cystic lesions (with emphysematous destruction and surrounding dense wall fibrosis) may represent a unique imaging pattern of CPFE, as they were present histologically in an autopsy study in over 70% of patients with CPFE, but never in patients with either isolated pulmonary fibrosis or isolated emphysema ^141^. The pattern of SRIF or AEF may also represent a unique histopathologic pattern of CPFE ^4,180^. Much work therefore remains to define CPFE morphologic subtypes and potential identification of signature pathogenetic pathways.

### Clinical utility

For the present, the acceptance of CPFE as a syndrome is mostly dependent on its perceived clinical utility. The strongest argument is that monitoring of disease progression cannot be reliably based on FVC in patients with CPFE: serial FVC trends, generally viewed as the cardinal monitoring measure in IPF, are less reliable in CPFE-IPF, with a lower prognostic significance than in the remaining IPF patients without emphysema. The high prevalence of lung cancer and PH further supports the designation of CPFE as a syndrome, especially with the perspective of therapeutic consequences ^281^.

Another approach to address whether the syndrome of CPFE is a distinct condition would be to demonstrate that its outcome differs from that of IPF alone. However, challenges in the diagnosis and quantification of CPFE hamper prognostic evaluation. As discussed above, difficulties comparing outcomes between patients with CPFE and those with fibrosis alone stem from the heterogeneity of CPFE, both for the emphysema and the fILD components, and from the need to quantify both components to adjust for severity of disease when studying outcome. However, in general, additional emphysema alerts the clinician of a greater likelihood of PH and greater mortality than might be expected for a given extent of fILD ^30,58,190^. In addition, patients with CPFE have a higher risk of lung cancer than those with IPF alone ^194,198^.

### CPFE as a discrete syndrome

Taken in their entirety, the considerations summarized above indicate that CPFE should be considered a *syndrome* based on distinct clinical features and pathogenetic considerations and to facilitate further potentially crucial pathogenetic research. Whether it might correspond to a single biologically unique entity in a proportion of cases warrants further study.

## Management

### General measures

There is a paucity of controlled data and no clinical practice guidelines to inform treatment decisions in patients with CPFE ^282^. Although some have advocated management based on a “treatable traits” approach (e.g. identifying disease phenotypes and possibly endotypes important for management in the individual patient) ^283,284^, there are no high-quality data indicating that treatment of emphysema or PH in the context of CPFE improves health outcomes of these patients. Management of CPFE as summarized in Table 13 is therefore typically extrapolated from approaches used in isolated COPD and from data in IPF trials in which patient sub-groups with CPFE have been explored.

Smoking cessation is appropriate in all patients who continue to smoke, as well as avoidance of any other potential inhalational exposures. Supplemental oxygen therapy is recommended in the context of resting hypoxemia ^285^, and may also have benefits when prescribed only for hypoxemia that occurs during exercise and nocturnally, even in those patients who are normoxemic at rest ^285,286^. Regular exercise and pulmonary rehabilitation are recommended for most patients with CPFE ^192^. Although no studies have evaluated pulmonary rehabilitation in CPFE, pulmonary rehabilitation and regular exercise are a cornerstone of management of patients with emphysema and are increasingly used in patients with fILD. As most exacerbations of both COPD and fILD are thought to be triggered by a respiratory tract infection (either from a virus or bacteria), influenza, pneumococcal, and COVID-19 vaccination are also recommended as per standard intervals, unless contraindicated ^287,288^. Referral for consideration of lung transplantation should be made early in the disease course for appropriate patients due to the progressive natural history of CPFE ^289^, particularly when complicated by PH.

### Treatment of pulmonary fibrosis

Decisions about pharmacologic treatment are guided by the underlying diagnosis of fILD ^288^. Management of pulmonary fibrosis in the setting of CPFE is informed by the landmark clinical trials of nintedanib and pirfenidone ^291–296^. Both antifibrotic medications slow progression of mild-to-moderate IPF and other subtypes of progressive pulmonary fibrosis by approximately 50% at 12 months. While patients with significant emphysema (greater than the volume of fibrosis on HRCT) and those with significant airflow obstruction have generally been excluded from these studies, the presence of emphysema in a proportion of patients might have contributed to slow decline in FVC in the placebo arm in CAPACITY 1 ^291^. A subgroup analysis of the IPF INPULSIS trials with nintedanib found no difference in the magnitude of the treatment effect with regards to the presence of mild-to-moderate emphysema ^297^. Importantly, in the INBUILD trial of nintedanib in fibrotic lung disease other than IPF, progressing despite management ^294^, the treatment effects were uniform across individual ILDs ^298^. Therefore, antifibrotic medications may have benefit in IPF patients with CPFE, and in other forms of pulmonary fibrosis with CPFE, progressing despite management. In patients with fILD other than IPF, combined with emphysema, including fHP and CTD-ILD, glucocorticoids and/or immunosuppressive therapy may be beneficial ^288^. However, there is a need to specifically study CPFE in future trials given its unique physiology. Specifically, the preserved FVC ^24^ and slower rates of FVC progression ^29^ indicate that FVC, the traditional endpoint for IPF trials, may be seriously flawed as a primary endpoint in CPFE, as discussed earlier.

### Treatment of pulmonary emphysema

Recognition of the individual phenotype of each patient is recommended given the lack of controlled data specific to the treatment of CPFE ^35^. Inhaled bronchodilators may have benefit in select patients with CPFE who have significant airflow limitation (ie. COPD)^299^, and one uncontrolled cohort study has suggested a possible improvement in FEV_1_ following the use of a combination of inhaled corticosteroid and long-acting bronchodilator ^286,299^. Further studies of inhaled bronchodilators with/without corticosteroids are needed in patients with CPFE due to the relatively well-preserved spirometric values ^24^.

Surgical or bronchoscopic lung volume reduction therapy removes emphysematous tissue, enabling relatively normal tissue to expand; however, most patients with CPFE would be precluded from such procedure given the frequently severe reduction in DLco ^300^. Bronchoscopic approach with endobronchial valves is generally safer, although no direct comparison with surgery was performed ^301^. It is uncertain, however, whether removal of emphysematous tissue will lead to improvements or worsening of lung mechanics in those with CPFE.

### Treatment of pulmonary hypertension

Management of PH in the presence of CPFE is based upon managing the underlying respiratory disorder, treating hypoxemia with supplemental oxygen, and ensuring optimal timing for lung transplant referral ^115^. Controlled data do not support the use of oral PH specific therapies ^191,302^, including endothelin receptor antagonists (bosentan, ambrisentan), phosphodiesterase-5 inhibitors (sildenafil, tadalafil), or stimulator of soluble guanylate cyclase (riociguat)^303^, although uncontrolled observational studies show possible benefit from PH therapies ^304,305^, and there are encouraging secondary endpoint trends in trials using sildenafil in IPF ^295,306,307^. Particular caution should be exercised, as treatment with ambrisentan and riociguat may be detrimental in patients with fILD ^308,309^ and especially those with CPFE ^310^. Recently, nebulized treprostinil improved 6-minute walk distance, decreased NT-pro-brain natriuretic peptide levels, improved FVC, and reduced the risk of clinical worsening compared to placebo in patients with ILD and group 3 precapillary PH confirmed by right heart catheterization ^281,311^; however, clinical implementation remains limited due to multiple challenges. To date, retrospective data have not demonstrated any survival benefit of PH therapy in patients with CPFE, and further research is required to specifically evaluate these therapies, particularly in those patients with preserved spirometry and “out of proportion PH”.

### Treatment of lung cancer

The overall approach to management of lung cancer in CPFE is similar to other populations, with prioritization of surgical resection where possible (e.g., stage I and II non-small cell lung cancer), multiple additional options considered in other situations (e.g., chemotherapy, targeted medications, radiotherapy), and palliation appropriate for many patients ^312^. Unfortunately, relatively more patients with CPFE are not candidates for various forms of treatment and complication rates are generally higher for those who are treated, with harm likely driven by the combined severity of emphysema and underlying fILD. For example, standard of care cancer treatment could not be instituted in 17% of patients with CPFE and lung cancer due to limitations in treatment directly attributable to CPFE ^116^.

CPFE is a risk factor for post-surgical morbidity and mortality compared to lung cancer without CPFE ^40,45,197,203^, with high rates of acute lung injury ^200^, acute disease exacerbations ^43,196,204^, and tumor recurrence ^201^. The risk of treatment-associated acute exacerbation of ILD is of particular concern in patients with CPFE, with increased rates of exacerbation following surgical resection, radiation, and many forms of chemotherapy. Lung-preserving resection options, improved anesthetic considerations, targeted medications, and stereotactic ablative radiotherapy may conceivably all reduce this risk to some extent ^312^, although there are currently limited direct data to guide risk estimation. Additional studies will continue to test the safety and efficacy of these treatment options in patients with fILD, with these results likely to be generalizable to patients with CPFE.

## Clinical trial perspectives

### Choice of endpoint

There have been a limited number of clinical trials on CPFE, in part due to its complicated pathophysiology and the lack of a standardized definition. The potential impact of emphysema (CPFE) on commonly used outcomes in COPD and ILD and the change of these variables over time is uncertain and presents difficulty when considering how to include and study these patients in clinical trials. In particular, the use of FVC as an endpoint is hampered in CPFE by its relative stability ^29^, despite disease progression and a high risk of mortality. The use of DLco is limited by the general functional severity of disease (i.e. floor effect), variability of measurement, and its multiple determinants ^313^. CPI is not validated as an endpoint. Mortality has been considered impracticable as a primary endpoint ^314^. Consideration could be given to a composite endpoint (e.g. death, respiratory hospitalization, or categorical FVC decline). However, composite endpoints are usually driven by, and are only as meaningful as, their least severe component ^315^. HRCT analysis of fibrosis either using visual methods, or future quantitative computer tools that can discriminate emphysema accurately, and/or blood biomarkers may be particularly useful if validated as endpoints.

One retrospective series suggesting that change in FEV1 (decline in FEV1 > 10% over 12 months) was the best physiologic predictor of increased risk of mortality in patients with at least moderate CPFE ^138^. Although further study is needed, these limited data may have important implications during the design and execution of future clinical trials.

### Patients with CPFE in idiopathic pulmonary fibrosis trials

The observation that serial change in FVC, now the favored primary endpoint in IPF treatment trials ^316^, is confounded by concurrent emphysema, has major implications for future IPF trial design ^29^. In future IPF trials, patients with a significant functional impact from concurrent emphysema are likely to be excluded. The approach taken in the CAPACITY and ASCEND trials of pirfenidone was to exclude patients with obstructive lung disease based on FEV1/FVC ratio < 0.7 or < 0.8, respectively ^291,293^. Thus, the effect of pirfenidone on patients with IPF and airflow obstruction is unknown. However, physiology variables are insensitive in excluding patients with emphysema in the setting of IPF ^29^, and imaging criteria such as extent of emphysema on HRCT may be more appropriate.

In the INPULSIS trials of nintedanib, patients with a FEV1/FVC ratio of < 0.7 were also excluded ^292^. A post-hoc analysis found that 39.6% of patients had emphysema (scored yes/no at baseline) and 38.8% had a FEV1/FVC ratio > 0.7 and ≤ 0.8 ^297^. The treatment effect of nintedanib versus placebo was similar between patients with and without emphysema, and when comparing different thresholds of FEV1/FVC (0.7 < FEV1/FVC < 0.8 or FEV1/FVC > 0.8 ^297^. Further study is needed to better understand the impact of presence and severity of CPFE and effect of treatment with pirfenidone or nintedanib.

## Relevance of CPFE for the non-specialist

Whilst most non-ILD pulmonary specialists and general practitioners have an appreciation for COPD, many will be less familiar with the diagnosis and treatment of CPFE.

In patients with clinical diagnoses of COPD, severely reduced DLco in the setting of minimal to moderate airflow obstruction indicates that additional investigations may be useful and especially chest HRCT. While emphysema alone may present with a disproportionate reduction in DLco, CPFE is considered, particularly given the high prevalence of ILAs on HRCT imaging (in ~8% of smokers aged over 60) and their association with restrictive lung deficits that can obscure features of airflow obstruction by spirometry ^127^. Although HRCT imaging is not currently considered standard of care in patients with COPD, it has been recently proposed in the diagnosis of COPD ^317^ and an increasing number of patients undergo imaging, either for lung cancer screening or as additional diagnostic workup for advanced treatments such as endobronchial valve placement. In such instances, HRCT findings may be the first clinical clue that fibrosis is also present. In COPD cohort studies, patients with ILAs have worse clinical outcomes than those without ILAs, including reduced exercise capacity ^318^ and increased all-cause mortality ^319^.

After identifying CPFE, additional history and diagnostic testing may be warranted as outlined in this document, similar to what is appropriate in patients with isolated ILD ^144,282^. Consultation with, or referral to, an ILD specialist may be helpful to determine if the patient is a candidate for ILD specific therapy, although further research is needed to better understand the optimal treatment of this patient population. In general, the presence of emphysema is associated with a worse outcome and a greater likelihood of PH than might be expected for a given extent of ILD. Future research is also needed for the evaluation of lung cancer risk in this population. While both IPF and COPD increase the risk for lung cancer compared to the general population, lung cancer risk for patients with CPFE (or ILAs and emphysema) may be elevated beyond emphysema or IPF alone ^148,194,198^. Such patients also have generally poor prognosis ^198^.

## Research priorities

The CPFE taskforce committee identified several gaps in our knowledge that need to be addressed, including: 1) to understand the pathogenetic mechanisms in CPFE; 2) to understand the pathobiology, disease behavior, and natural history of CPFE; 3) to improve methods that allow an early diagnosis; and 4) to evaluate potential therapeutic opportunities. Questions and statements identifying some of the topics that were considered important for research are listed in Table 14.

## Conclusions

CPFE is characterized by a wide variety of appearances and patterns on chest HRCT and when available on histopathology. Clustering of pulmonary fibrosis and emphysema (regardless of the type of fILD), the frequency of associated comorbidities and complications especially PH and lung cancer, the relevance for disease progression monitoring, and the involvement of pathogenetic pathways shared by both components, suggest that CPFE should be considered a syndrome. Despite numerous case series and studies, many important questions remain unanswered. This ATS/ERS/JRS/ALAT Research Statement offers research definition and classification criteria and identifies major research priorities that will better delineate this entity, understand its pathogenesis, and guide its management.

## Supplementary Material

Supplemenary data

## Figures and Tables

**Figure 14:**
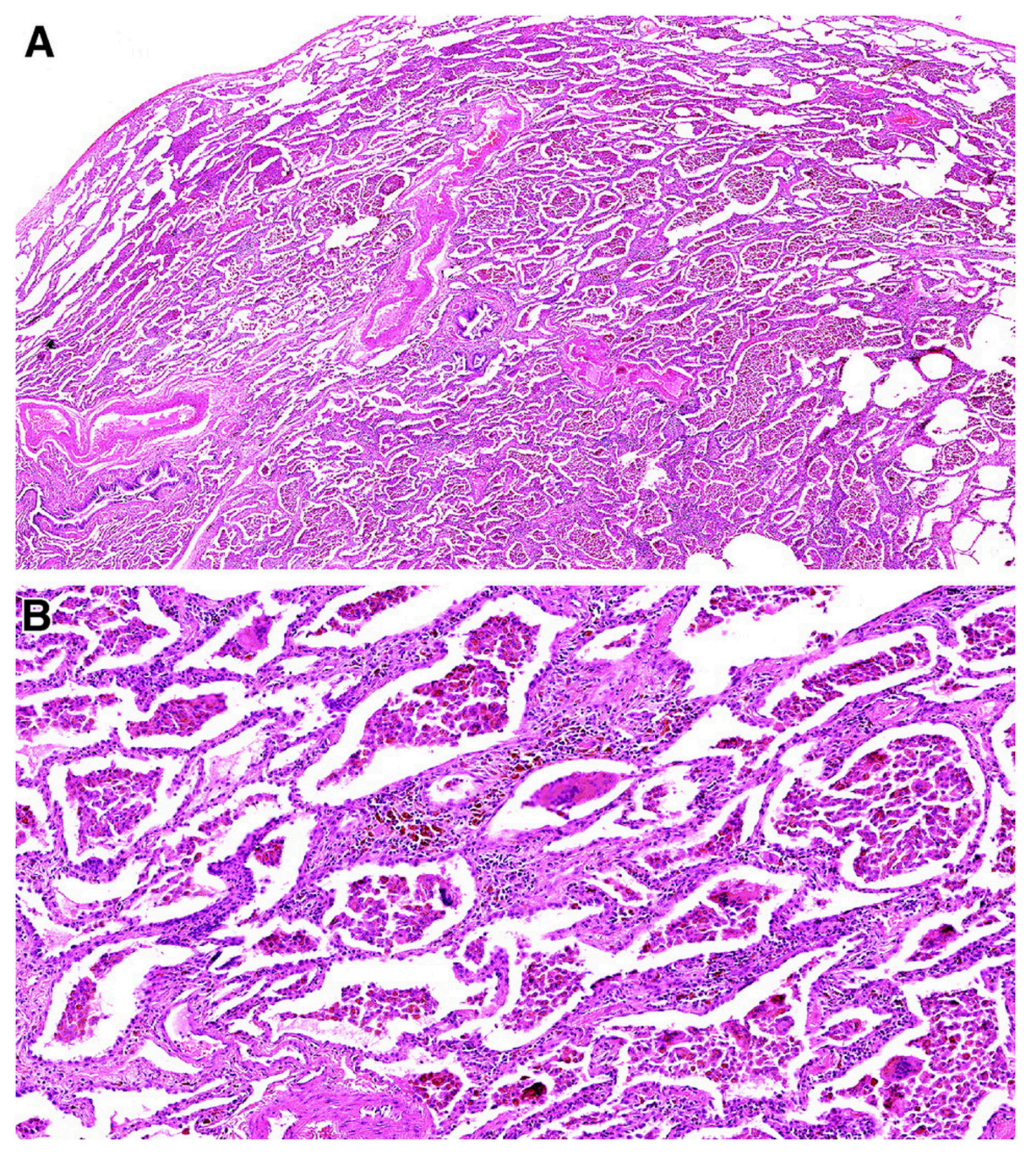
Desquamative interstitial pneumonia (DIP). A. Low magnification photomicrograph showing a relatively uniform interstitial pneumonia compounded by prominent clusters of pigments alveolar macrophages. B. Higher magnification photomicrograph shows the interstitial inflammation that distinguishes DIP from SRIF (compare to Figure 14B). Hematoxylin and eosin stain.

**Table 1:** Milestones in the Description of Combined Pulmonary Fibrosis and Emphysema

Year	Author (Reference)	Description
1948	Mallory *et al*. (7)	First published case report of right heart failure due to CPFE (in a 27-yr-old woman)
1948	Robbins *et al*. (8)	Description of areas of fibrosis interspersed with areas of emphysema (thin-walled bullae or blebs) on chest radiograph
1966	Tourniaire *et al*. (9)	Report of 4 patients with CPFE who developed PH, with severe alteration of gas transfer and hypoxemia, and coexistence of paraseptal emphysema and perilobular fibrosis at autopsy
1982	Niewoehner *et al*. (10)	Hypothesized that pulmonary fibrosis and emphysema represent divergent responses to a common injury (e.g., cigarette smoking) and may share some pathways
1988	Westcott *et al*. (11)	Postmortem examination of the lungs of patients with end-stage pulmonary fibrosis showing that traction bronchiectasis was absent or mild in 3 patients with CPFE, ascribed to a reduction in elastic recoil due to emphysema
1990	Wiggins *et al*. (12)	Report of 8 patients with CPFE, with preservation of lung volumes and severe reduction in DL_co_ ascribed to the combined impact of the two disease processes
1991	Schwartz *et al*. (13)	Increased residual volume and more impaired gas exchange in patients with IPF who smoked cigarettes. IPF reduces the likelihood of developing physiologic correlates of airflow obstruction among cigarette smokers
1993	Hiwatari *et al*. (14)	Report of 9 patients with CPFE
1993	Strickland *et al*. (15)	Description of areas of honeycombing and bullous emphysematous changes on HRCT in IPF Traction on small airways due to interstitial fibrosis prevents the small airway collapse typical of smoking-related emphysema and results in preserved ventilation in areas of bullous destruction and overall increase in lung volumes
1997	Doherty *et al*. (16)	Preserved lung volumes in patients with IPF who smoked
1997	Wells *et al*. (17)	Higher lung volumes and lower DL_co_ in presence of emphysema in patients with IPF, after adjusting for the extent of fibrosis on CT
1999	Hoyle *et al*. (18)	Overexpression of platelet-derived growth factor in mice provoked lung pathology characterized by emphysematous changes, inflammation, and fibrosis
2003	Wells *et al*. (19)	Description of the CPI, which correlates with the extent of fibrosis on HRCT independently of the presence of emphysema and predicts mortality more accurately than DL_co_ and other pulmonary function variables
2005	Cottin *et al*. (1)	Retrospective analysis of 61 patients with CPFE. PH is frequent and determines a dismal prognosis. Suggestion to individualize CPFE as an entity
2005	Lundblad *et al*. (20) and comment (21)	Morphologic abnormalities consistent with both pulmonary fibrosis and emphysema, in association with generalized lung inflammation, in a mouse model of TNF-α overexpression, supporting a common pathogenetic linkage of both conditions
2006	Yousem *et al*. (22)	Pathological description of respiratory bronchiolitis-associated interstitial lung disease with fibrosis
2008	Kawabata *et al*. (4)	Pathological description of airspace enlargement with fibrosis
2009	Cottin *et al*. (2)	Suggestion that CPFE is a syndrome
2010	Katzenstein *et al*. (23)	Pathological description of smoking-related interstitial fibrosis

*Definition of abbreviations*: CPFE = combined pulmonary fibrosis and emphysema; CPI = composite physiologic index; CT = computed tomography; HRCT = high-resolution computed tomography; IPF = idiopathic pulmonary fibrosis; PH = pulmonary hypertension; TNF = tumor necrosis factor.

**Table 2:** Frequency Estimates of Combined Pulmonary Fibrosis and Emphysema across Different Patient Populations

Population	Reported Frequency (*%*)
General population	Unknown
Idiopathic pulmonary fibrosis	8–67
Idiopathic interstitial pneumonia	26–54
Lung cancer, with underlying idiopathic interstitial pneumonia or idiopathic pulmonary fibrosis	55–58
Rheumatoid arthritis–interstitial lung disease	8–58
Systemic sclerosis–interstitial lung disease	5–12
Lung cancer	3–10
Lung cancer screening cohort	0.04
Cohort undergoing chest computed tomography	3–7

**Table 3:** Exposures and Etiologies That Are Associated with Combined Pulmonary Fibrosis and Emphysema

Variables Associated with CPFE	References
Risk factors and demographics	
Cigarette smoking	24, 32, 34, 38, 43, 45, 50, 54–58
Male sex	1, 32, 38, 43, 45, 46, 55–57, 60, 61
Diseases	
Idiopathic pulmonary fibrosis	24–33
Connective tissue disease	49–52, 62, 64, 79
ANCA-associated vasculitis	65, 66, 80, 81
Hypersensitivity pneumonitis	47, 78, 82, 83
Inhalational exposure	
Coal dusts	46, 70, 71
Asbestos	74–77
Silica and mineral dust	84
Other inhalational exposures	72, 73, 85–87
Rare genetic variants	
Telomerase-related genes (*TERT*, *RTEL1*)	88–92
Surfactant-related genes (*SFTPC*, *ABCA3*)	93–98
Other genes (*Naf1*, *PEPD*)	99, 100
Genetic polymorphisms	
*MMP-9* and *TGF-β-1* genes	101–103
*AGER* gene	104
rs2736100 (*TERT*), rs2076295 GG (*DSP*)	105

*Definition of abbreviations*: ANCA = antineutrophil cytoplasmic antibodies; CPFE = combined pulmonary fibrosis and emphysema; MMP = matrix metalloprotease; TGF = transforming growth factor.

**Table 4:** sMain Characteristics of Pulmonary Function in Combined Pulmonary Fibrosis and Emphysema

Pulmonary Function Test Measurement	Typical Abnormality Seen in CPFE	Typical Abnormality Seen in fILD without Emphysema
FVC	Decreased or normal (but preserved compared with idiopathic pulmonary fibrosis alone)	Decreased
FEV_1_	Decreased or normal	Decreased
FEV_1_/FVC	Variable (normal, decreased, or increased)	Normal or increased
TLC	Variable (normal, decreased, or increased)	Decreased
FRC	Variable (normal, decreased, or increased)	Decreased
Residual volume	Variable (normal, decreased, or increased)	Decreased
DL_co_	Disproportionately decreased	Decreased
Transfer coefficient for carbon monoxide	Severely decreased	Normal or decreased
Saturation during exercise	Severe desaturation	Desaturation
Peak oxygen uptake	Decreased	Decreased

*Definition of abbreviations*: CPFE = combined pulmonary fibrosis and emphysema; fILD = fibrotic interstitial lung disease.

**Table 5:** Definitions of High- and Low-Attenuation Parenchymal Features Frequently Visualized on HRCT Imaging in Patients with Combined Pulmonary Fibrosis and Emphysema

Attenuation Level of Imaging Feature on CT	Parenchymal Feature	Description
High attenuation	Smooth ground-glass opacity	Increased-opacity parenchyma occurring on the background of normal-appearing lung
	Coarse ground-glass opacity	Increased-opacity parenchyma with overlying reticulation or traction bronchiectasis
	Reticulation	Linear opacities representing thickened inter- and intralobular septae
Low attenuation	Cysts	A round parenchymal lucency with a well-defined interface with normal lung
	Honeycomb cysts	Clustered cystic airspaces (3–10 mm in diameter) with well-defined walls that are usually subpleural
	Traction bronchiectasis, bronchiolectasis	Irregular bronchial and bronchiolar dilatation caused by surrounding retractile pulmonary fibrosis. Adjacent lung is typically of high attenuation
	Air trapping	Parenchymal areas with reduced attenuation that lack volume reduction on expiratory imaging
	Emphysema: paraseptal	Subpleural and peribronchovascular regions of low attenuation separated by intact interlobular septa. May be associated with bullae
	Emphysema: centrilobular	Centrilobular areas of low attenuation, usually without visible walls. Nonuniform distribution, predominantly located in upper lung zones
	Emphysema: panacinar	Generalized decreased attenuation of lung parenchyma with a decrease in blood vessel caliber in the affected lung. Typically lower-zone-predominant location

*Definition of abbreviations*: CT = computed tomography; HRCT = high-resolution computed tomography.

**Table 6:** Histopathological Features of Smoking-related Interstitial Fibrosis and Other Patterns of Fibrotic Interstitial Lung Disease in Combined Pulmonary Fibrosis and Emphysema

Pattern of Fibrosis	Distribution	Fibroblast Foci	Honeycomb Change	Interstitial Inflammation
SRIF (5, 23)	Patchy, subpleural, peribronchiolar	Rare	Rare	Absent
DIP (180)	Diffuse	Rare	Absent	Present
UIP, probable UIP (144, 180)	Patchy, subpleural, interlobular septa	Present	Present	Patchy, mild (may be more extensive in areas of honeycombing)
F-NSIP (180)	Diffuse	Rare	Absent	Present
Indeterminate (180, 184)	Patchy or diffuse	±	±	±

*Definition of abbreviations*: DIP = desquamative interstitial pneumonia; F-NSIP = fibrotic nonspecific interstitial pneumonia; SRIF = smoking-related interstitial fibrosis; UIP = usual interstitial pneumonia.

**Table 7:** Variables Associated with Death among Patients with Combined Pulmonary Fibrosis and Emphysema

Variable	Association with Increased Mortality	Reference
Demographic	Older age	27
Biologic	Negative antinuclear antibodies	25
	Increased red cell distribution width	220
Physiologic		
DL_co_	Lower DL_co_ %predicted	27, 41, 115, 203, 220, 221
FVC	<50% predicted	32
FEV_1_	Decline of ⩾10% over 12 mo	138
CPI	⩾45	119, 188, 220
Oxygen saturation	Oxygen saturation on room air < 90%	59
Oxygen requirement	Home oxygen use	203
Complications and comorbidities		
Pulmonary hypertension	Elevated pulmonary artery pressure, right ventricular dysfunction, increased pulmonary vascular resistance, low cardiac index, or main pulmonary artery diameter/ascending aortic diameter ratio (depending on study)	1, 27, 32, 49, 115, 119, 203, 222
Lung cancer	Presence of lung cancer	41, 57, 59, 141, 203, 206, 219
Acute exacerbations	Acute exacerbations of fibrosis	206
Radiologic	Presence of UIP pattern	27, 188
	Presence or extent of honeycombing	27, 41, 203
	Extent of fibrosis (fibrosis score)	59, 221

*Definition of abbreviations*: CPI = composite physiologic index; UIP = usual interstitial pneumonia.

**Table 8:** Main Features Shared by Pulmonary Fibrosis and Emphysema

Domain	Features Shared by Pulmonary Fibrosis and Emphysema
Clustering of pulmonary fibrosis and emphysema	Emphysema on HRCT is more prevalent than expected in IPF and RA-ILD (28) compared with smokers without pulmonary fibrosis Emphysema occurs with a lower pack-year smoking history in IPF, RA-ILD, and SSc-ILD (28, 49, 51) compared with smokers without pulmonary fibrosis Emphysema is more prevalent in patients with nonspecific interstitial pneumonia than in smokers without ILD (223)
Telomere dysfunction and the accelerated aging processes	Gene variants associated with telomere maintenance (90, 91, 99, 231–237) N.B.: Gene variants with opposing effects on risk of IPF or COPD also reported (227–229) Abnormal telomere shortening, cell senescence, mitochondrial dysfunction, and other aging-associated processes (238, 239) Experimental telomere dysfunction results in either pulmonary fibrosis or emphysema (88, 240)
Gene expression and interactome	Shared gene expression and splicing (241, 242) N.B.: Distinct gene expression reported between emphysematous and fibrotic lesions in patients with CPFE (230)
Environmental exposures, smoking, and epigenomic reprogramming	Alterations in cigarette-smoking alveoli (161, 243–246) Epigenetic modifications, including changes in DNA methylation and histone modifications (247, 248)
Mechanical forces	Severe fibrosis may provoke dilation of airway lumens of terminal bronchioles and more visible airways on HRCT (224, 249) Increased risk of progression from probable UIP to UIP in patients with emphysema (225) Clustering of paraseptal emphysema and HRCT pattern of UIP (52, 226)
Enzymatic activity	Exaggerated enzymatic activity of matrix metalloproteinases (250–268) Involvement of fibrocytes (an important source of matrix metalloproteinases) (269–272) Increased elastolytic and neutrophil elastase activity (273–277)
Lung development and lung function trajectories	Hypothesis that abnormal mechanisms early in life may predispose to development of emphysema and fibrosis (as reported for the development of emphysema alone [278, 279])
Experimental models characterized by both emphysema and fibrosis and/or inflammation	Transgenic mice overexpressing platelet-derived growth factor (18) Transgenic mice overexpressing tumor necrosis factor-α (20) Mice deficient in surfactant protein-C (98)

*Definition of abbreviations*: COPD = chronic obstructive pulmonary disease; CPFE = combined pulmonary fibrosis and emphysema; HRCT = high - resolution computed tomography; ILD = interstitial lung disease; IPF = idiopathic pulmonary fibrosis; N.B. = nota bene (note); RA = rheumatoid arthritis; SSc = systemic sclerosis; UIP = usual interstitial pneumonia.

**Table 9:** Summary of Various Definitions Used by Studies to Identify Patients with Combined Pulmonary Fibrosis and Emphysema

Definition for CPFE	Number of Studies	Total Number of Patients with CPFE
No clear definition	3	259
Definitions using radiologic features only		
Definition proposed by Cottin *et al*. (1) as outlined below: i) Diffuse parenchymal lung disease with significant pulmonary fibrosis, defined as all of the following: Reticulation with peripheral and basal predominance Honeycombing Architectural distortion Minimal ground glass and/or consolidation ii) Areas of decreased attenuation with thin (<1 mm) or no walls that are upper-zone predominant	52	3,426
Minimum disease extent for fibrosis or emphysema required	10	975
Presence of any fibrosis and emphysema that is different than criteria by Cottin *et al*.	22	1,804
Definitions using a combination of domains		
Clinical ILD diagnosis + emphysema on imaging	5	332
Clinical abnormalities (e.g., crackles on auscultation, abnormal gas exchange) + fibrosis and emphysema on imaging	2	55
Airflow obstruction confirmed by spirometry + fibrosis on imaging	2	184

*Definition of abbreviations*: CPFE = combined pulmonary fibrosis and emphysema; ILD = interstitial lung disease.

**Table 10:** Proposed Terminology to Describe Combined Pulmonary Fibrosis and Emphysema

Component	Characteristics	Examples
Fibrosis	Pattern*	UIP Fibrotic NSIP Desquamative interstitial pneumonia Unclassifiable fILD AEF, SRIF Etc.
	Assessment*	Radiology Histopathology
Emphysema	Predominant pattern on HRCT	Paraseptal Centrilobular Mixed (paraseptal and centrilobular) Admixed (with fibrosis) Thick-walled large cysts Emphysema, pattern not specified
Etiology or diagnostic category of fILD	ILD diagnosis*	Idiopathic Smoking-related CTD-ILD fHP Other
Extent	Extent of fibrosis	% of total lung volume
	Extent of emphysema	% of total lung volume
Distribution of fibrosis versus emphysema	Separate entities	Emphysema in the upper zones and fibrosis in the lung bases, with no or little overlap of emphysema and fibrosis in between
	Progressive transition	Progressive transition from emphysema lesions to fibrosis, with significant overlap or admixture in mid areas
	Paraseptal emphysema with fibrosis	Paraseptal emphysema with progressive increase in size toward lung bases where ground glass and reticulation coexist
	Thick-walled large cysts	Thick-walled large cysts suggesting AEF or SRIF
	Admixed pattern	Admixed emphysema and fibrosis increasing from upper lobes to lower lobes
Comments	Physiology or chest HRCT	Predominance of fibrosis vs. emphysema
	Echocardiography Right heart catheterization	Suspected pulmonary hypertension Pulmonary hypertension

*Definition of abbreviations*: AEF = airspace enlargement with fibrosis; CTD = connective tissue disease; fHP = fibrotic hypersensitivity pneumonitis; fILD = fibrotic interstitial lung disease; HRCT = high-resolution computed tomography; NSIP = nonspecific interstitial pneumonia; SRIF = smoking-related interstitial fibrosis; UIP = usual interstitial pneumonia.

* It is generally useful to include these items when describing CPFE in the individual patient: a case with UIP on HRCT and emphysema in a smoker may be described as smoking-related “CPFE-radiologic UIP.” A case with fibrotic NSIP on biopsy and emphysema may be described as idiopathic “CPFE-histologic NSIP.” A case of UIP pattern and emphysema on HRCT in a nonsmoker with RA may be described as “RA-associated CPFE-radiologic UIP.”

**Table 11:** Proposed *Research Definition* of Combined Pulmonary Fibrosis and Emphysema (to Serve Research Purposes) and Classification Criteria of Combined Pulmonary Fibrosis and Emphysema *Clinical Syndrome* (Intended to Have Clinical Relevance)

*Research definition* of CPFE	Coexistence of both pulmonary fibrosis and emphysema Patients must have both criteria on HRCT: Emphysema of any subtype at HRCT defined as well-demarcated areas of low attenuation delimitated by a very thin wall (⩽1 mm) or no wall^*†‡^ and involving at least 5% of total lung volume^§^ Lung fibrosis of any subtype^‖^
	
Classification criteria of CPFE *clinical syndrome*: These additional criteria serve research purposes and may be considered depending on the objective of the study	Patients must have CPFE (*see above*) and one or more of the following: Emphysema extent ⩾15% of total lung volume^§¶^ Relatively preserved lung volumes and airflow with very or disproportionately decreased DL_co_, especially in patients with limited extent of HRCT abnormalities, and in the absence of pulmonary hypertension Precapillary pulmonary hypertension considered not related to the sole presence of emphysema (FEV_1_ > 60%), fibrosis (FVC > 70%), or the etiological context (e.g., absence of connective tissue disease)

*Definition of abbreviations*: CPFE = combined pulmonary fibrosis and emphysema; HRCT = high-resolution computed tomography; ILD = interstitial lung disease.

* Emphysema generally predominates in the upper lobes but may be present in other areas of the lung or may be admixed with fibrosis.

† Emphysema may be replaced by thick-walled large cysts >2.5 cm in diameter (CPFE, thick-walled large cysts variant).

‡ Surgical lung biopsy is not required if HRCT pattern is diagnostic. However, CPFE is suggested if lung biopsies show emphysema and any pattern of pulmonary fibrosis; emphysema can then be quantified on HRCT.

§ Emphysema extent is assessed visually by an experienced radiologist (*see* online supplement). Emphysema extent <5% is unlikely to impact physiology or outcome and is more open to interobserver disagreement.

‖ Signs of fibrosis on HRCT in a patient with ILD include architectural distortion, traction bronchiectasis, honeycombing, and volume loss. Caution must be exerted for the identification of honeycombing in patients with associated emphysema. Ground-glass attenuation may be present. Interstitial lung abnormalities (170) are not sufficient to consider CPFE.

¶ Emphysema extent >15% extent is associated with relatively stable FVC over time. Several studies have used a 10% threshold; however, association with outcome in FVC has not been demonstrated.

**Table 12:** Main Arguments in Favor of and Against Combined Pulmonary Fibrosis and Emphysema Being a Syndrome

	In Favor of a Syndrome	Against a Syndrome
Pathogeny	Clustering of emphysema and fILD to a greater extent than would be expected from the pack-year smoking history (in patients with IPF, idiopathic nonspecific interstitial pneumonia, rheumatoid arthritis-ILD, and systemic sclerosis-ILD)	No primary pathogenetic pathways unique to CPFE identified
Presentation	High prevalence of thick-walled large cysts on HRCT. High prevalence of airspace enlargement with fibrosis on histopathology	No histopathological or radiologic feature specific for CPFE
Complications and comorbidities	Increased risk of PH compared with IPF for a given extent of fibrosis Increased risk of lung cancer compared with IPF or emphysema alone	The risk of PH is dependent on total extent of fibrosis and emphysema, the quantification of which is challenging
Mortality	Increased mortality compared with IPF for a given extent of fibrosis	The mortality risk is dependent on total extent of fibrosis and emphysema, the quantification of which is challenging
Monitoring	FVC alone is not appropriate to monitor disease progression and a primary endpoint in clinical trials. Consider screening and/or monitoring for PH and lung cancer	—
Diagnosis	Identification of honeycombing and of the UIP pattern is challenging in patients with concurrent emphysema	—

*Definition of abbreviations*: CPFE = combined pulmonary fibrosis and emphysema; fILD = fibrotic interstitial lung diseases; HRCT = high-resolution computed tomography; IPF = idopathic pulmonary fibrosis; PH = pulmonary hypertension; UIP = usual interstitial pneumonia.

**Table 13:** Key Points of Current Practice Management in Patients with Combined Pulmonary Fibrosis and Emphysema

General measures	Smoking cessation Pulmonary rehabilitation Vaccination against influenza, *Pneumococcus*, and COVID-19 Supplemental oxygen therapy as per recommendations (286, 290) Consider lung transplantation
Pulmonary fibrosis	Lack of evidence specific to CPFE Individual management and decisions about pharmacologic treatment (e.g., antifibrotic medication, immunosuppressants) should be discussed by a multidisciplinary team based on type of fILD, relative predominance of fibrosis versus emphysema, and disease progression Consider antifibrotic medications at first presentation of patients with IPF with CPFE, and in other forms of pulmonary fibrosis with CPFE, progressing despite management
Pulmonary emphysema	Lack of evidence specific to CPFE Consider inhaled bronchodilators and inhaled corticosteroids as per indications in COPD
Complications and comorbidities	Lack of evidence related to treatment of PH specific to CPFE Management of comorbidities, especially cardiovascular disease and lung cancer

*Definition of abbreviations*: COPD = chronic obstructive pulmonary disease; COVID-19 = coronavirus disease; CPFE = combined pulmonary fibrosis and emphysema; fILD = fibrotic interstitial lung disease; IPF = idiopathic pulmonary fibrosis; PH = pulmonary hypertension.

**Table 14:** Research Priorities in Combined Pulmonary Fibrosis and Emphysema

Epidemiology	Registries of IPF and other ILDs running in different countries and regions should include a specific column for the presence of “associated emphysema” and indicate criteria used, to have a more precise knowledge about its frequency
Biopathology	Does the development of emphysema in IPF and non-IPF ILDs share similar pathogenetic mechanisms? Why and how do fibrosis and emphysema relate temporally in patients with CPFE, particularly in never-smokers? Are there differences in the pathobiology and natural history of CPFE according to the timeline of development? Is the UIP or UIP-like pattern the main conductive thread? By molecular behavior? By mechanical behavior? By both? Emphysema in fHP and autoimmune diseases: is there a role for autoimmunity? Is there a difference in the type of emphysema in IPF, fHP, and autoimmune diseases? (extent, localization, paraseptal, or centrilobular) Paraseptal and centrilobular emphysema, different mechanisms? Smokers vs. nonsmokers? An experimental model of CPFE is necessary. Transgenic mice? Wild-type mice with double injury (e.g., cigarette smoke and bleomycin or herpesvirus)? To compare gene expression and pathways in emphysema alone, IPF alone, and fibrotic and emphysematous lesions in IPF combined with emphysema Single-cell RNA sequencing comparing emphysematous and fibrotic lesions from the same CPFE lungs The role of autoimmunity in CPFE and acute exacerbations of fibrosis in CPFE
Diagnosis	What are the histopathologic correlates of CPFE using the proposed definition? Research of biomarkers that allow early diagnosis Can biomarkers that have been developed for IPF distinguish IPF from CPFE? Can longitudinal evaluation of emphysema and interstitial lung abnormalities or fibrosis in lung cancer screening populations delineate distinct morphological phenotypes and progression patterns of CPFE? How to characterize and quantify imaging abnormalities in order to harmonize future studies? Develop automated tools capable of reliably distinguishing emphysema from honeycombing and traction bronchiectasis Determine minimum extents of involvement for both emphysema and fibrosis components on chest HRCT using reproducible methods of measurement Is CPFE a unitary diagnosis, or are there distinct phenotypes? Research of new imaging modalities, such as optical coherence tomography or targeted contrast agents in positron emission tomography or magnetic resonance imaging, that may allow early diagnosis or distinguish IPF from CPFE
Outcome	Are imaging and histopathologic patterns of CPFE associated with distinct outcomes? What is the disease trajectory in CPFE? Can it be occasionally self-limited, or is it always progressive? How to monitor disease progression in CPFE? What biomarkers can be used to predict the outcome in CPFE?
Management	Should patients with CPFE be treated, in addition to the fibrotic disorder, with COPD drugs? What are the effects of pirfenidone or nintedanib on the clinical course of these patients? How to differentiate exacerbations of COPD from exacerbations of fibrosis, and how to manage them? Do “shielding” measures protect against acute worsening events?
Clinical trials	What are the effects of new antifibrotic drugs on the clinical course of CPFE? Should patients with CPFE be evaluated separately from those with IPF and without emphysema? How to factor in the use of inhaled corticosteroids and bronchodilators or pulmonary rehabilitation used to treat COPD or emphysema? How to evaluate the comorbidities and complications of CPFE and to take them into account in study design? What is the best physiologic measure to follow the patients and the best endpoint for trials?

*Definition of abbreviations*: COPD = chronic obstructive pulmonary disease; CPFE = combined pulmonary fibrosis and emphysema; fHP = fibrotic hypersensitivity pneumonitis; HRCT = high-resolution computed tomography; ILD = interstitial lung disease; IPF = idiopathic pulmonary fibrosis; UIP = usual interstitial pneumonia.
