# Supplementary material for: Syndrome of Combined Pulmonary Fibrosis and Emphysema: An official research statement from American Thoracic Society (ATS), European Respiratory Society (ERS), Japanese Respiratory Society (JRS), and Asociación Latinoamericana de Tórax (ALAT)
Source: Am J Respir Crit Care Med. Author manuscript; Available in PMC 2023 Oct 13. (PMC7615200; doi:10.1164/rccm.202206-1041ST)
Supplement: Supplemenary data [file EMS188584-supplement-Supplemenary_data.pdf]

ONLINE DATA SUPPLEMENT

SYNDROME OF COMBINED PULMONARY FIBROSIS AND EMPHYSEMA

An Official American Thoracic Society (ATS), European Respiratory Society (ERS), Japanese Respiratory Society (JRS), and Asociación Latinoamericana de Tórax (ALAT) Research Statement

Authors: Vincent Cottin* [1], Moises Selman § [2], Yoshikazu Inoue § [3], Alyson W. Wong [4], Tamera J. Corte [5], Kevin R. Flaherty [6], MeiLan K. Han [7], Joseph Jacob [8], Kerri A. Johannson [9], Masanori Kitaichi [10], Joyce S. Lee [11], Alvar Agusti [12], Katerina M. Antoniou [13], Pauline Bianchi [14], Fabian Caro [15], Matias Florenzano [16], Liam Galvin [17], Tae Iwasawa [18], Fernando J. Martinez [19], Rebecca L. Morgan [20], Jeffrey L. Myers [21], Andrew G. Nicholson [22], Mariaelena Occhipinti [23], Venerino Poletti [24], Margaret L. Salisbury [25], Don D. Sin [26], Nicola Sverzellati [27], Thomy Tonia [28], Claudia Valenzuela [29], Christopher J. Ryerson # [30], Athol U. Wells # [31] on behalf of the American Thoracic Society, European Respiratory Society, Japanese Respiratory Society, and Asociación Latinoamericana de Tórax.

§ these authors contributed equally, and are co-second authors

# these authors contributed equally, and are co-last authors

Approved by the American Thoracic Society on May 18, 2022, European Respiratory Society on June 1, 2022, Japanese Respiratory Society on May 15, 2022, and Asociación Latinoamericana de Tórax on May 11, 2022.

Author affiliations:

1. Vincent Cottin, MD, PhD, National Reference Center for Rare Pulmonary Diseases, Louis Pradel Hospital, Hospices Civils de Lyon, University of Lyon, INRAE, Lyon, France, ORCID 0000-0002-5591-0955, vincent.cottin@chu-lyon.fr,

2. Moises Selman, MD, Instituto Nacional de Enfermedades Respiratorias "Ismael Cosío Villegas", Mexico City, Mexico, mselmanl@yahoo.com.mx. ORCID: 0000-0002-1022-4783

3. Yoshikazu Inoue, MD, Ph.D., Osaka, Japan, ORCID 0000-0003-3994-874X, giichiyi@me.com

4. Alyson W. Wong, MD, University of British Columbia, Vancouver, Canada, awong@providencehealth.bc.ca

5. Tamera J. Corte, MD, PhD, Royal Prince Alfred Hospital and University of Sydney, Sydney, Australia, ORCID: 0000-0002-5076-8929, tameracorte@mac.com

6. Kevin R. Flaherty, MD, University of Michigan, Ann Arbor, USA, flaherty@umich.edu

7. MeiLan K. Han, MD, University of Michigan, Ann Arbor, USA, mrking@umich.edu

8. Joseph Jacob, MD, University College London, London, United Kingdom, j.jacob@ucl.ac.uk

9. Kerri A Johannson, MD, Calgary, Canada, ORCID 000-0003-1205-5511, Kerri A. Johannson, Departments of Medicine and Community Health Sciences, University of Calgary, Calgary, AB, Canada, kerri.johannson@ahs.ca

10. Masanori Kitaichi, MD, Ph.D., NHO Minami Wakayama Medical Center, Tanabe, Japan, kitaichi.masanori.fq@mail.hosp.go.jp

11. Joyce S. Lee, MD, University of Colorado Denver Anschutz Medical Campus, School of Medicine, Aurora, CO, USA, ORCID 0000-0002-5758-8365, JOYCE.LEE@CUANSCHUTZ.EDU

12. Alvar Agusti, MD, PhD, Respiratory Institute, Hospital Clinic, University of Barcelona, IDIBAPS, CIBERES, Barcelona, Spain, aagusti@clinic.cat

13. Katerina M. Antoniou, MD, Laboratory of Molecular and Cellular Pneumonology, Department of Respiratory Medicine, University of Crete, Heraklion, Greece, kantoniou@uoc.gr

14. Pauline Bianchi, Senior Vice President, Pulmonary Fibrosis Foundation, paulinebianchi@yahoo.com

15. Fabian Caro, MD, Hospital de Rehabilitación Respiratoria "María Ferrer", Buenos Aires, Argentina, ORCID 0000-0003-2484-1923, fabiancarodoc@gmail.com

16. Matias Florenzano, MD, Instituto Nacional Del Tórax, Santiago, Chile, ORCID number 0000-0002-3244-0903, mflorenzano@gmail.com

17. Liam Galvin, European idiopathic pulmonary fibrosis and related disorders federation, liam.galvin@eu-ipff.org

18. Tae Iwasawa, MD, Kanagawa Cardiovascular and Respiratory Center, Yokohama, Japan, tae_i_md@wb3.so-net.ne.jp

19. Fernando Martinez, MD, New York, USA, fjm2003@med.cornell.edu

20. Rebecca L. Morgan, PhD, McMaster University, Hamilton, Canada, ORCID 0000-0002-1012-4897, morganrl@mcmaster.ca

21. Jeffrey L. Myers, MD, University of Michigan, Ann Arbor, USA, myerjeff@med.umich.edu

22. Andrew G. Nicholson, DM, Royal Brompton and Harefield Hospitals, Guy's and St Thomas' NHS Foundation Trust and National Heart and Lung Institute, Imperial College, London, United Kingdom, ORCID 0000-0003-3257-102X, a.nicholson@rbht.nhs.uk

23. Mariaelena Occhipinti, MD, PhD, Radiomics, Liege, Belgium, mariaelena.occhipinti@gmail.com

24. Venerino Poletti, MD, University of Bologna, Ospedale Morgagni, Forlì, Italy, venerino.poletti@gmail.com

25. Margaret L. Salisbury, MD, Vanderbilt University Medical Center, Nashville, USA, ORCID 0000-0001-8217-6955, margaret.salisbury@vumc.org

26. Don D. Sin, MD, University of British Columbia, Vancouver, Canada, Don.Sin@hli.ubc.ca

27. Nicola Sverzellati, MD, Scienze Radiologiche, Department of Medicine and Surgery, University of Parma, Italy, nicola.sverzellati@unipr.it

28. Thomy Tonia, MSc, Institute of Social and Preventive Medicine, University of Bern, Switzerland, thomy.tonia@ersnet.org

29. Claudia Valenzuela, MD, Pulmonology Department, Hospital Universitario de la Princesa, Departamento Medicina, Universidad Autónoma de Madrid, 28049 Madrid, Spain, claudiavale@hotmail.com,

30. Christopher J. Ryerson, MD, University of British Columbia, Vancouver, Canada, Chris.Ryerson@hli.ubc.ca

31. Athol U. Wells, MD, Imperial college, London, UK, RBHILD@rbht.nhs.uk

*Corresponding author: Vincent Cottin, MD. Department of Respiratory Medicine, National Coordinating Reference Centre for Rare Pulmonary Diseases, Louis Pradel Hospital, Hospices Civils de Lyon, 28 avenue Doyen Lepine, 69677 Lyon cedex, Lyon, France; and Claude Bernard University Lyon 1, F-69008, INRAE, member of OrphaLung, RespiFil, Radico-ILD, and ERN-LUNG, Lyon, France. vincent.cottin@chu-lyon.fr. Tel +33 427857700.

Table of contents

Systematic Review Methodology ... 6

Historical perspective ... 7

Pathogenesis and putative mechanisms ... 10

Clustering of pulmonary fibrosis and emphysema ... 10

Gene variants ... 10

Gene expression and interactome ... 11

Environmental exposures, smoking, and epigenomic reprogramming ... 11

Telomere dysfunction and the accelerated aging processes ... 11

Mechanical forces ... 12

Lung development and lung function trajectories ... 12

Enzymatic activity ... 12

Experimental models ... 13

Visual scoring of emphysema: ... 14

Supplementary tables ... 15

Table E1. Search strategies for systematic review using MEDLINE. ... 15

Table E2. Search strategies for systematic review using EMBASE. ... 17

Table E3. Description of imaging features of CPFE with its two principal components, emphysema and fibrosis, as reported in literature. ... 18

References ... 20

Supplementary figures ... 29

Figure E1. Systematic review search results. ... 29

Figure E2. Emphysematous lesions in patients with lung fibrosis who have never smoked. 30

Figure E3. Minimal extent of pulmonary fibrosis and emphysema on CT imaging. 31

Figure E4. Visual scoring of emphysema 32

Figure E5. Respiratory bronchiolitis (RB) and smoking-related interstitial fibrosis (SRIF) in a patient with ILD. 33

Figure E6. Smoking-related interstitial fibrosis (SRIF) and extensive respiratory bronchiolitis (RB) resembling desquamative interstitial pneumonia (DIP). 34

Figure E7. Advanced Langerhans cell histiocytosis (LCH) mimicking combined pulmonary fibrosis and emphysema (CPFE). 35

Figure E8. Comparison of smoking related interstitial fibrosis (SRIF) and usual interstitial pneumonia (UIP). 37

Figure E9. Usual interstitial pneumonia (UIP) and emphysema in a patient with combined pulmonary fibrosis and emphysema (CPFE). 38

Figure E10. Definition and thresholds used to define CPFE.. 40

Systematic Review Methodology

The search strategy was published previously and the search was updated on December 1, 2021 (tables E1 and E2) (1). We searched MEDLINE and EMBASE databases for all original research articles published in English between January 1, 2000 and December 1, 2021, which included patients with evidence of both pulmonary fibrosis and emphysema in any distribution. Studies were limited to this time period based on the contemporary definition of IPF published in the 2000 clinical practice guidelines (2), except for the historical perspective and for pathogenetic considerations, for which no limit was set to the publication time. All forms of original research were included (e.g., randomized control trials and observational studies), however case series required at least 10 patients to be included. Search strategies were created in consultation with an academic librarian and the protocol was registered in PROSPERO (3). Screening was performed independently by two reviewers and studies were excluded based on pre-determined criteria: if the study was not original research, not CPFE-related, had a sample size < 10, non-English, or involved non-human subjects. Disagreements were resolved by consensus after assessment by a third reviewer. The search of 15,095 citations resulted a total of 72 eligible full-text publications. Summaries of relevant manuscripts were provided to the subcommittees responsible for drafting each main section of the taskforce document. The search focused on identifying studies to answer the research statement's clinical research questions; however, studies that were excluded in the search were used to support the historical and pathogenesis content.

Historical perspective

In 1948, Mallory et al described a 27-year old woman with pathologically confirmed IPF and emphysema, with "crepitant rales" on lung auscultation, finger clubbing, enlargement of the right ventricle and the main pulmonary artery on chest radiograph, and *cor pulmonale* at autopsy (4). This was the first published report of right heart failure due to CPFE. In the discussion, Dr Sprague commented: "*I have been impressed with what we call IPF and emphysema, with absolutely normal chest films*". The same year, Robbins reported IPF and emphysema on chest radiography (5) with fibrosis interspersed with emphysema, described as thin-walled bullae or blebs.

In 1966, Tourniaire et al reported four CPFE patients with pulmonary hypertension (PH) confirmed by right heart catheterization, highlighting severe reduction in gas transfer, hypoxemia and the coexistence of paraseptal emphysema and perilobular fibrosis at autopsy (6).

In 1982, Niewoehner and Hoidal hypothesized, based on experimental results, that pulmonary fibrosis and emphysema represent divergent responses to a common injury (e.g., cigarette smoking) and may share some pathways (7).

In 1990, Wiggins et al reported eight patients with IPF characterized by concurrent smoking-related emphysema and a unique pulmonary function phenotype, consisting of preservation of lung volumes (median forced vital capacity (FVC) 99%, range 68-131%) and severe reduction in gas transfer (median DLco 32%, range 9-35%), ascribed to the combined impact of the two disease processes (8). This observation was supported by subsequent case reports (9, 10). In 1988, in a post-mortem study of patients with end-stage pulmonary fibrosis, traction bronchiectasis was absent or mild in three patients with CPFE, attributed to a reduction in elastic recoil due to emphysema (11).

In 1991, Schwartz et al reported that smokers with IPF have increased residual volume and more impaired gas exchange, with a reduced likelihood of physiologic correlates of airflow obstruction (12).

In 1993, Strickland et al evaluated false positive diagnoses of pulmonary embolism in IPF, and found that areas with normal ventilation but absent perfusion on ventilation-perfusion scintigraphy were associated with honeycombing or, less frequently, bullous emphysematous change on HRCT (13). The

latter finding was believed to reflect traction on small airways due to interstitial fibrosis, preventing the small airway collapse typical of smoking related emphysema and resulting in preserved ventilation in areas of bullous destruction (with a corresponding overall increase in measured lung volumes).

In 1997, Wells et al (14) described that the presence of emphysema in patients with IPF was associated with higher lung volumes, lower DLco, and decreased gas exchange after adjusting for the extent of fibrosis on CT. Doherty et al reported preserved lung volumes in past or current smokers with IPF (10). In 2003, Wells et al (15) developed a formula (the "composite physiologic index", CPI) that correlated strongly with the extent of fibrosis on HRCT, whilst excluding the functional consequences of emphysema, and predicted mortality more accurately than DLco and other pulmonary function variables. The CPI was also applicable to other idiopathic fibrotic disorders (such as fibrotic non-specific interstitial pneumonia).

In 2005, Cottin et al reported a retrospective analysis of 61 patients with emphysema, predominantly in the upper zones, and fibrotic ILD, predominantly in the lower zones (16). The characteristic pulmonary function phenotype described by Wiggins et al was confirmed as a defining feature of CPFE. PH was diagnosed at presentation in 54% of patients and was a malignant prognostic determinant. The authors proposed CPFE as a discrete entity, arguing that "it deserves the terminology of syndrome as a result of the association of symptoms and clinical manifestations, each with a probability of being present increased by the presence of the other" (17). The description of morphologic abnormalities consistent with pulmonary fibrosis and emphysema, in association with generalized lung inflammation, in a mouse model of TNF-alpha overexpression (18), supported the view that coexistence of IPF and emphysema may be more than just coincidental with a common pathogenetic linkage to smoking (19).

In 2008, Kawabata et al described the pathological entity of airspace enlargement with fibrosis (20), a frequent finding in CPFE. Similar findings applicable to CPFE were described by other groups, including respiratory bronchiolitis-associated interstitial lung disease with fibrosis (21), respiratory bronchiolitis with fibrosis (22), and smoking-related interstitial fibrosis (23, 24).

More recently, several series cited later in this document have contributed to a more complete description of CPFE, and etiological factors other than tobacco smoking have been identified.

Pathogenesis and putative mechanisms

The pathogenetic mechanisms leading to the coexistence of emphysema with IPF and other fILDs remain unclear. Likewise, it is uncertain whether IPF and non-IPF/ILD are causally linked with emphysema or are coincidental disorders that share some mechanisms. Specifically, it is not known whether pulmonary emphysema develops first, followed by the subsequent development of pulmonary fibrosis (here, CPFE may represent two aging-associated diseases running independently), or if emphysema and fibrosis develop concurrently (potentially with shared mechanisms from the outset). Given its aggressive natural course, it is probable as suggested by some series (16) that pulmonary fibrosis (especially of the UIP type) develops after emphysema, although it might be preceded by interstitial lung abnormalities (25).

Clustering of pulmonary fibrosis and emphysema

Emphysema on HRCT is approximately five-fold more prevalent than expected in both IPF and RA-ILD compared to smokers without pulmonary fibrosis after adjustment for age, sex and the pack-year smoking history (26). In both IPF and RA-ILD, as well as in systemic sclerosis–associated ILD, the pack-year smoking history associated with the presence of emphysema was lower than reported in large cohorts of smokers (26-28). Emphysema is strikingly more prevalent in patients with nonspecific interstitial pneumonia than in healthy smokers (29). Thus, it appears that all four fILDs cluster with the presence of emphysema, raising the possibility of a shared predilection triggered by smoking in a subset of patients with ILD.

Gene variants

Gene variants common to IPF and COPD include some that are associated with an increased risk for one entity but are protective against the other (30-32). However, gene variants affecting telomere biology have been linked to familial and sporadic IPF, familial COPD and CPFE (33-36). Importantly, abnormal shortening of telomeres is found in both IPF and COPD (37, 38), and experimental models provoking telomere dysfunction can result in either pulmonary fibrosis or emphysema (39, 40).

Gene expression and interactome

Analysis of the lung transcriptome in IPF, COPD (with different extensions of emphysema), and normal lungs identified 214 shared genes and some convergent disease-associated alterations in gene expression and splicing (41). Several biologic processes were identified, including up-regulation of the p53/hypoxia pathway in both IPF and emphysema. A more recent study designed to recognize overlapping molecular network modules between COPD and IPF revealed an overlapping module consisting of 19 genes and several concordant pathways, including extracellular matrix remodeling and mitogen-activated protein kinase (42).

One study that explored gene expression in the same CPFE lungs suggested that the genes upregulated in fibrotic lesions are functionally different from those overexpressed in the emphysematous lesions (43). The genes upregulated in fibrotic lesions were related to the immune system, cytoskeleton, and cellular adhesion, while the set of genes upregulated in the emphysematous lesions were associated with cell membrane structures, vascular growth and biology, and lung development.

Environmental exposures, smoking, and epigenomic reprogramming

IPF and emphysema are strongly associated with cigarette-smoking related alveolar insult (44-47), which can provoke epigenetic modifications, including changes in DNA methylation and histone modifications (48, 49). Such mechanisms may mediate how cigarette smoke may modulate the expression of some genes in response to various stimuli.

Telomere dysfunction and the accelerated aging processes

Patients with a variety of ILDs have abnormal telomere shortening (50-52), with several mutations affecting genes related to telomere biology in both familial and sporadic ILD (53-55). Exaggerated telomere shortening, cell senescence, mitochondrial dysfunction, and other aging-associated processes are found in progenitor and resident cells in both IPF and COPD (56-63). These concurrent processes may contribute to the coexistence of different lung injury patterns in the same lung. Overall, data available suggest that some genetic predilections are separate for the two processes of pulmonary fibrosis and emphysema, but are overlaid by predilections that are common to the two processes.

Mechanical forces

Severe fibrosis may provoke "traction emphysema" or "alveolo-ectasis" by excessive mechanical forces, similar to the genesis of traction bronchiectasis and bronchiolectasis (64). This may contribute to the development of emphysema in patients with fILD, particularly paraseptal emphysema, that has a stronger association than centrilobular emphysema with both fILD severity and a HRCT pattern of UIP (65, 66). Supporting bidirectionality, the risk of honeycombing progression in patients with probable UIP and emphysema is higher than in those without emphysema, suggesting that emphysema might also contribute to the progression of UIP lesions (67).

Lung development and lung function trajectories

Along with exposure to smoking, abnormal lung development before or after birth can contribute to emphysema in adulthood (68, 69). There is evidence in COPD, mostly in patients with emphysema, that lung function trajectories vary throughout the course of disease. It is conceivable, although not supported by evidence, that a combination of environmental and genetic risk factors can alter the lung function trajectory, with a propensity to develop both emphysema and fILD. Further research is needed to understand the abnormal mechanisms early in life that can predispose to development of emphysema and/or fibrosis, and their impact on subsequent lung function trajectories.

Enzymatic activity

Exaggerated enzymatic activity of matrix metalloproteinases (MMPs) is present in COPD, IPF, and non-IPF ILD (70-87). There is an increased number of neutrophils in the airways and alveolar walls of patients with COPD, with marked upregulation of MMP-8 and MMP-9 (71, 72). Alveolar macrophages and pneumocytes overexpress MMP-1 and MMP-2, consistently upregulated in the lung epithelium of IPF patients, with a potential role in lung remodeling and the activation of biological mediators (70, 76, 79, 80). MMP-1 and MMP-8 are also increased in RA-ILD (80, 81). MMP-7, the most upregulated MMP in IPF and also overexpressed in non-IPF ILD (78, 82, 83, 87, 88), is associated with centrilobular and paraseptal emphysema in COPD (89). MMP-28 (epilysin) promotes chronic lung inflammation and emphysema in mice exposed to cigarette smoke, and is up-regulated in

COPD and in the alveolar epithelium of patients with IPF (74-76). Fibrocytes, an important source of MMPs, migrate to fibrotic tissue and also to the airways of patients with COPD (90-93). Finally, elastolytic activity, usually increased in COPD, is also detected in IPF through the increase of desmosine/isodesmosine in plasma and BAL (94-97). Moreover, neutrophil elastase has a profibrotic effect promoting fibroblast activation (98).

Experimental models

CPFE has been revealed in several experimental models including transgenic mice overexpressing platelet derived growth factor or tumor necrosis factor-alpha (18, 99, 100). Interestingly, surfactant protein-C-deficient 129/Sv mice develop severe emphysema and fibrosis, which worsens with age and is associated with increased macrophage MMP-2 and MMP-9 activity (100).

Overall, numerous studies show that many pathways and mechanisms are shared between fibrosis and emphysema.

Visual scoring of emphysema

Scoring of CT scans for the presence of emphysema can be performed by quantifying emphysema at six CT levels (101) or by quantifying emphysema on a lobar basis (102). The six levels to be assessed include: the level of the aortic arch; 1 cm below the carina; at the pulmonary venous confluence; equidistant between the third and the fifth levels; 2 cm above the right hemidiaphragm; and 1 cm below the right hemidiaphragm (101). However, these six levels were primarily chosen for the purpose of scoring of fibrosis and have been weighted towards the lower zones of the lungs where fibrosis is most common. They may therefore underappreciate apical emphysema, which may be better captured by lobar scoring, as it gives equal weighting to the upper and lower lobes (101). When using lobar scoring, the lingula is considered a sixth lobe, which commences at the level of the lingula bronchus origin. Emphysema mixed with fibrosis, most typically seen in the lower lobes, should be considered in the emphysema volume and not separately.

When using either scoring system, the minimal score assigned when emphysema is present in a lobe is 5%. Therefore, a single small focus of emphysema in a lobe or at a single lung level should score 5%. When emphysematous lesions appear dotted throughout a lobe or lung level (Figure 9), estimating a percentage of emphysema for the lobe can be challenging. A technique that may help here is to visually aggregate all the emphysematous foci within the lobe/level together and estimate what fraction of the total lobar/level volume the cumulative emphysematous lung comprises, i.e. estimate whether the emphysema comprises a half, a third, a quarter or a fifth of a lobe/level. If less than this, determine whether it comprises 15%, 10% or just 5% of the lobe/level volume. Once emphysema has been scored in each of the six lobes/levels, sum the percentages of emphysema and divide by 6 to derive the total percentage of emphysema in the patients lungs.

Supplementary tables

Table E1. Search strategies for systematic review using MEDLINE.

**Ovid MEDLINE(R) In-Process & Other Non-Indexed Citations and Ovid MEDLINE(R) <1946 to Present>**		
**#**	**Searches**	**Results**
1	exp pulmonary fibrosis/ or idiopathic pulmonary fibrosis/	21543
2	Idiopathic Pulmonary Fibrosis.mp.	8430
3	pulmonary fibrosis.mp.	28381
4	exp Lung Diseases, Interstitial/	57669
5	interstitial pneumon*.mp.	9633
6	interstitial lung disease*.mp	9955
7	parenchymal lung disease*.mp.	669
8	or/1-7	85563
9	Pulmonary Emphysema/	15678
10	emphysema/	7013
11	lung diseases, obstructive/ or bronchitis, chronic/ or pulmonary disease, chronic obstructive/	57111
12	emphysema*.mp.	35220
13	(chronic* adj3 bronchiti*).mp.	11375
14	(obstruc* adj3 (pulmonary or lung* or airway* or airflow* or bronch* or respirat*)).mp.	114265
15	COPD.mp.	44159
16	COAD.mp.	339
17	COBD.mp.	20
18	or/9-17	153569
19	8 and 18	6091
20	limit 19 to humans	5344

21	limit 20 to yr="2000-Current"	2816

Table E2. Search strategies for systematic review using EMBASE.

Embase <1974 to Present>		
**#**	**Searches**	**Results**
1	exp lung fibrosis/	67150
2	fibrosing alveolitis/	22590
3	idiopathic pulmonary fibrosis.mp.	14025
4	pulmonary fibrosis.mp.	28126
5	fibrosing alveolitis.mp.	23037
6	lung fibrosis.mp.	34923
7	exp interstitial lung disease/	74250
8	interstitial pneumon*.mp.	20268
9	interstitial lung disease*.mp.	25418
10	parenchymal lung disease*.mp.	1186
11	or/1-10	117845
12	lung emphysema/	13252
13	emphysema/	16971
14	chronic obstructive lung disease/	125329
15	emphysema*.mp.	44536
16	chronic obstructive lung disease.mp.	126175
17	(obstruc* adj2 (pulmonary or lung* or airway* or airflow* or respirat*)).mp.	187767
18	COPD.mp.	84140
19	COAD.mp.	465
20	COBD.mp.	23
21	or/12-20	233939
22	11 and 21	12089
23	limit 22 to human	10730
24	limit 23 to yr="2000-Current"	8486

Table E3. Description of imaging features of CPFE with its two principal components, emphysema and fibrosis, as reported in literature.

Condition	Categories	Main characteristics	References
**Emphysema**	*HRCT subtype*	Centrilobular: 4-50%<br>Paraseptal: 2-55%<br>Panlobular: 15%<br>Mixed: 10-94%<br>- Paraseptal predominant in CPFE and centrilobular in emphysema alone<br>- Paraseptal more common in CPFE-UIP and centrilobular in CPFE-non-UIP	(103-110)
	Distribution	Upper predominant: 62-74%<br>Lower predominant: 14%<br>diffuse: 19-79%<br>unilateral: 5%	(105, 108, 111, 112)
	Extent	**Semi-quantitative HRCT:**<br>- emphysema extent: 7-20%<br>(trace emphysema in 29% cases / >10% in 8% cases; <15% in 68% cases vs ≥ 15% in 32% cases)<br>- Upper >> Lower: 14% vs 5%<br>- Emphysema extent greater in CPFE-UIP compared to CPFE-non-UIP	(27, 109, 110, 112-118)
		**Quantitative HRCT:**<br>- Emphysema extent: 8-13%<br>- %LAA lower or equal in CPFE *vs* emphysema alone<br>- %LAA and destructed lung area increase more over 5 years than in CPFE *vs* emphysema alone	(107, 108, 119, 120)
**Fibrosis**	*HRCT pattern*	UIP: 13-81%<br>Possible UIP: 25%<br>NSIP-like: 4-34%<br>Unclassifiable: 47%<br>- A complex pattern with predominant reticular opacities in 15% cases<br>- presence of *honeycombing* similar among groups with different ILD subtypes	(16, 104, 106, 109, 110, 113, 114, 121-123)
	Distribution	Basal predominant: 100%	(108, 111)

	Extent	**Semi-quantitative HRCT:**<br>- fibrosis extent: 1-42%<br>- fibrosis score in CPFE lower or equal to IPF alone<br>- fibrosis score higher in CPFE-UIP than in CPFE-non-UIP (12 vs 9%)<br>- fibrosis score similar across groups with different emphysema extent<br>- ILD extent is not associated with the extent or location of emphysema<br>- *Follow-up*: increase in *GGO* extent at follow-up greater than in IPF<br>- *Coarseness scores* in both IPF and RA-ILD are associated with fibrosis extent and presence of emphysema; coarseness of fibrosis is greater in IPF compared to non-IPF ILD<br>- *Reticulation, honeycombing*, and fibrosis extent similar between smoking-related interstitial fibrosis, UIP alone and CPFE-UIP groups	(26, 27, 109, 110, 112, 113, 115, 116, 118, 123-126)
		**Quantitative HRCT:**<br>- fibrosis score: 1.4%<br>- mean change in HAA% similar to fibrosis alone groups	(107, 119)
**Additional features**	*Cysts*	- CPFE >>> IPF<br>- Thick-walled large cysts in 58% cases (considered to correspond to airspace enlargement with fibrosis [AEF], no in IPF	(111, 127, 128)
	Vascular	- Cross-sectional area of small pulmonary vessels <5mm^2 greater than in COPD patients	(120)
	Lung cancer	- Located in subpleural regions, around fibrotic more than emphysematous areas	(129-131)

*quantitative HRCT analysis performed on low-dose HRCT scans.

Abbreviations: GGO: ground-glass opacity; %HAA: high attenuation area; %LAA: low attenuation area; IPF: idiopathic pulmonary fibrosis; NSIP: nonspecific interstitial pneumonia; RA-ILD: rheumatoid arthritis associated ILD; UIP: usual interstitial pneumonia.

References

1. Wong AW, Liang J, Cottin V, Ryerson CJ. Diagnostic Features in Combined Pulmonary Fibrosis and Emphysema: A Systematic Review. *Ann Am Thorac Soc* 2020; 17: 1333-1336.

2. American Thoracic Society. Idiopathic pulmonary fibrosis: diagnosis and treatment. International consensus statement. American Thoracic Society (ATS), and the European Respiratory Society (ERS). *Am J Respir Crit Care Med* 2000; 161: 646-664.

3. Wong A, Ryerson CJ, Liang C, Cottin V. Combined pulmonary fibrosis and emphysema: a systematic review and meta-analysis. PROSPERO; 2019. p. https://www.crd.york.ac.uk/prospero/display_record.php?RecordID=126108.

4. Mallory TB, Castleman B, Parris EE. Case records of the Massachusetts general hospital. Case 34191. *New Engl J Med* 1948; 238: 667-671.

5. Robbins LL. Idiopathic pulmonary fibrosis: roentgenologic findings. *Radiology* 1948; 51: 459-467.

6. Tourniaire A, Tartulier M, Blum J, Deyrieux F. [Early detection of pulmonary circulatory sound in pure fibrosis and in fibrosis complicated by emphysema]. *Presse Med* 1966; 74: 2139-2144.

7. Niewoehner DE, Kleinerman J, Rice DB. Pathologic changes in the peripheral airways of young cigarette smoker's. *N Engl J Med* 1974; 291: 755-758.

8. Wiggins J, Strickland B, Turner-Warwick M. Combined cryptogenic fibrosing alveolitis and emphysema: the value of high resolution computed tomography in assessment. *Respir Med* 1990; 84: 365-369.

9. Hiwatari N, Shimura S, Takishima T. Pulmonary emphysema followed by pulmonary fibrosis of undetermined cause. *Respiration* 1993; 60: 354-358.

10. Doherty MJ, Pearson MG, O'Grady EA, Pellegrini V, Calverley PM. Cryptogenic fibrosing alveolitis with preserved lung volumes. *Thorax* 1997; 52: 998-1002.

11. Westcott JL, Cole SR. Traction bronchiectasis in end-stage pulmonary fibrosis. *Radiology* 1986; 161: 665-669.

12. Schwartz DA, Merchant RK, Helmers RA, Gilbert SR, Dayton CS, Hunninghake GW. The influence of cigarette smoking on lung function in patients with idiopathic pulmonary fibrosis. *Am Rev Respir Dis* 1991; 144: 504-506.

13. Strickland NH, Hughes JM, Hart DA, Myers MJ, Lavender JP. Cause of regional ventilation-perfusion mismatching in patients with idiopathic pulmonary fibrosis: a combined CT and scintigraphic study. *AJR* 1993; 161: 719-725.

14. Wells AU, King AD, Rubens MB, Cramer D, du Bois RM, Hansell DM. Lone cryptogenic fibrosing alveolitis: a functional-morphologic correlation based on extent of disease on thin-section computed tomography. *Am J Respir Crit Care Med* 1997; 155: 1367-1375.

15. Wells AU, Desai SR, Rubens MB, Goh NS, Cramer D, Nicholson AG, Colby TV, du Bois RM, Hansell DM. Idiopathic pulmonary fibrosis: a composite physiologic index derived from disease extent observed by computed tomography. *Am J Respir Crit Care Med* 2003; 167: 962-969.

16. Cottin V, Nunes H, Brillet PY, Delaval P, Devouassoux G, Tillie-Leblond I, Israel-Biet D, Court-Fortune I, Valeyre D, Cordier JF. Combined pulmonary fibrosis and emphysema: a distinct underrecognised entity. *Eur Respir J* 2005; 26: 586-593.

17. Cottin V, Cordier JF. The syndrome of combined pulmonary fibrosis and emphysema. *Chest* 2009; 136: 1-2.

18. Lundblad LK, Thompson-Figueroa J, Leclair T, Sullivan MJ, Poynter ME, Irvin CG, Bates JH. Tumor necrosis factor-alpha overexpression in lung disease: a single cause behind a complex phenotype. *Am J Respir Crit Care Med* 2005; 171: 1363-1370.

19. Cottin V, Cordier JF. Combined pulmonary fibrosis and emphysema: an experimental and clinically relevant phenotype. *Am J Respir Crit Care Med* 2005; 172: 1605; author reply 1605-1606.

20. Kawabata Y, Hoshi E, Murai K, Ikeya T, Takahashi N, Saitou Y, Kurashima K, Ubukata M, Takayanagi N, Sugita H, Kanauchi S, Colby TV. Smoking-related changes in the background lung of specimens resected for lung cancer: a semiquantitative study with correlation to postoperative course. *Histopathology* 2008; 53: 707-714.

21. Yousem SA. Respiratory bronchiolitis-associated interstitial lung disease with fibrosis is a lesion distinct from fibrotic nonspecific interstitial pneumonia: a proposal. *Mod Pathol* 2006; 19: 1474-1479.

22. Reddy TL, Mayo J, Churg A. Respiratory bronchiolitis with fibrosis. High-resolution computed tomography findings and correlation with pathology. *Ann Am Thorac Soc* 2013; 10: 590-601.

23. Katzenstein AL, Mukhopadhyay S, Zanardi C, Dexter E. Clinically occult interstitial fibrosis in smokers: classification and significance of a surprisingly common finding in lobectomy specimens. *Hum Pathol* 2010; 41: 316-325.

24. Katzenstein AL. Smoking-related interstitial fibrosis (SRIF): pathologic findings and distinction from other chronic fibrosing lung diseases. *J Clin Pathol* 2013; 66: 882-887.

25. Washko GR, Hunninghake GM, Fernandez IE, Nishino M, Okajima Y, Yamashiro T, Ross JC, Estepar RS, Lynch DA, Brehm JM, Andriole KP, Diaz AA, Khorasani R, D'Aco K, Sciurba FC, Silverman EK, Hatabu H, Rosas IO. Lung volumes and emphysema in smokers with interstitial lung abnormalities. *N Engl J Med* 2011; 364: 897-906.

26. Antoniou KM, Walsh SL, Hansell DM, Rubens MR, Marten K, Tennant R, Hansel T, Desai SR, Siafakas NM, du Bois RM, Wells AU. Smoking-related emphysema is associated with idiopathic pulmonary fibrosis and rheumatoid lung. *Respirology* 2013; 18: 1191-1196.

27. Antoniou KM, Margaritopoulos GA, Goh NS, Karagiannis K, Desai SR, Nicholson AG, Siafakas NM, Coghlan JG, Denton CP, Hansell DM, Wells AU. Combined Pulmonary Fibrosis and Emphysema in Scleroderma-Related Lung Disease Has a Major Confounding Effect on Lung Physiology and Screening for Pulmonary Hypertension. *Arthritis Rheumatol* 2016; 68: 1004-1012.

28. Champtiaux N, Cottin V, Chassagnon G, Chaigne B, Valeyre D, Nunes H, Hachulla E, Launay D, Crestani B, Cazalets C, Jego P, Bussone G, Berezne A, Guillevin L, Revel MP, Cordier JF, Mouthon L. Groupe d'Etudes et de Recherche sur les Maladies Orphelines Pulmonaires. Combined pulmonary fibrosis and emphysema in systemic sclerosis: A syndrome associated with heavy morbidity and mortality. *Semin Arthritis Rheum* 2019; 49: 98-104.

29. Marten K, Milne D, Antoniou KM, Nicholson AG, Tennant RC, Hansel TT, Wells AU, Hansell DM. Non-specific interstitial pneumonia in cigarette smokers: a CT study. *Eur Radiol* 2009.

30. Hobbs BD, de Jong K, Lamontagne M, Bossé Y, Shrine N, Artigas MS, Wain LV, Hall IP, Jackson VE, Wyss AB, London SJ, North KE, Franceschini N, Strachan DP, Beaty TH, Hokanson JE, Crapo JD, Castaldi PJ, Chase RP, Bartz TM, Heckbert SR, Psaty BM, Gharib SA, Zanen P, Lammers JW, Oudkerk M, Groen HJ, Locantore N, Tal-Singer R, Rennard SI, Vestbo J, Timens W, Paré PD, Latourelle JC, Dupuis J, O'Connor GT, Wilk JB, Kim WJ, Lee MK, Oh YM, Vonk JM, de Koning HJ, Leng S, Belinsky SA, Tesfaigzi Y, Manichaikul A, Wang XQ, Rich SS, Barr RG, Sparrow D, Litonjua AA, Bakke P, Gulsvik A, Lahousse L, Brusselle GG, Stricker BH, Uitterlinden AG, Ampleford EJ, Bleecker ER, Woodruff PG, Meyers DA, Qiao D, Lomas DA, Yim JJ, Kim DK, Hawrylkiewicz I, Sliwinski P, Hardin M, Fingerlin TE, Schwartz DA, Postma DS, MacNee W, Tobin MD, Silverman EK, Boezen HM,

Cho MH. Genetic loci associated with chronic obstructive pulmonary disease overlap with loci for lung function and pulmonary fibrosis. *Nat Genet* 2017; 49: 426-432.

31. Sakornsakolpat P, Prokopenko D, Lamontagne M, Reeve NF, Guyatt AL, Jackson VE, Shrine N, Qiao D, Bartz TM, Kim DK, Lee MK, Latourelle JC, Li X, Morrow JD, Obeidat M, Wyss AB, Bakke P, Barr RG, Beaty TH, Belinsky SA, Brusselle GG, Crapo JD, de Jong K, DeMeo DL, Fingerlin TE, Gharib SA, Gulsvik A, Hall IP, Hokanson JE, Kim WJ, Lomas DA, London SJ, Meyers DA, O'Connor GT, Rennard SI, Schwartz DA, Sliwinski P, Sparrow D, Strachan DP, Tal-Singer R, Tesfaigzi Y, Vestbo J, Vonk JM, Yim JJ, Zhou X, Bossé Y, Manichaikul A, Lahousse L, Silverman EK, Boezen HM, Wain LV, Tobin MD, Hobbs BD, Cho MH. Genetic landscape of chronic obstructive pulmonary disease identifies heterogeneous cell-type and phenotype associations. *Nat Genet* 2019; 51: 494-505.

32. Fingerlin TE, Murphy E, Zhang W, Peljto AL, Brown KK, Steele MP, Loyd JE, Cosgrove GP, Lynch D, Groshong S, Collard HR, Wolters PJ, Bradford WZ, Kossen K, Seiwert SD, du Bois RM, Garcia CK, Devine MS, Gudmundsson G, Isaksson HJ, Kaminski N, Zhang Y, Gibson KF, Lancaster LH, Cogan JD, Mason WR, Maher TM, Molyneaux PL, Wells AU, Moffatt MF, Selman M, Pardo A, Kim DS, Crapo JD, Make BJ, Regan EA, Walek DS, Daniel JJ, Kamatani Y, Zelenika D, Smith K, McKean D, Pedersen BS, Talbert J, Kidd RN, Markin CR, Beckman KB, Lathrop M, Schwarz MI, Schwartz DA. Genome-wide association study identifies multiple susceptibility loci for pulmonary fibrosis. *Nat Genet* 2013; 45: 613-620.

33. Armanios M. Telomerase and idiopathic pulmonary fibrosis. *Mutation research* 2012; 730: 52-58.

34. Stanley SE, Chen JJ, Podlevsky JD, Alder JK, Hansel NN, Mathias RA, Qi X, Rafaels NM, Wise RA, Silverman EK, Barnes KC, Armanios M. Telomerase mutations in smokers with severe emphysema. *J Clin Invest* 2015; 125: 563-570.

35. Stanley SE, Gable DL, Wagner CL, Carlile TM, Hanumanthu VS, Podlevsky JD, Khalil SE, DeZern AE, Rojas-Duran MF, Applegate CD, Alder JK, Parry EM, Gilbert WV, Armanios M. Loss-of-function mutations in the RNA biogenesis factor NAF1 predispose to pulmonary fibrosis-emphysema. *Sci Transl Med* 2016; 8: 351ra107.

36. Petrovski S, Todd JL, Durheim MT, Wang Q, Chien JW, Kelly FL, Frankel C, Mebane CM, Ren Z, Bridgers J, Urban TJ, Malone CD, Finlen Copeland A, Brinkley C, Allen AS, O'Riordan T, McHutchison JG, Palmer SM, Goldstein DB. An Exome Sequencing Study to Assess the Role of Rare Genetic Variation in Pulmonary Fibrosis. *Am J Respir Crit Care Med* 2017; 196: 82-93.

37. Alder JK, Chen JJL, Lancaster L, Danoff S, Su S-c, Cogan JD, Vulto I, Xie M, Qi X, Tuder RM, Phillips JA, 3rd, Lansdorp PM, Loyd JE, Armanios MY. Short telomeres are a risk factor for idiopathic pulmonary fibrosis. *Proc Natl Acad Sci U S A* 2008; 105: 13051-13056.

38. Tsuji T, Aoshiba K, Nagai A. Alveolar cell senescence in patients with pulmonary emphysema. *Am J Respir Crit Care Med* 2006; 174: 886-893.

39. Alder JK, Guo N, Kembou F, Parry EM, Anderson CJ, Gorgy AI, Walsh MF, Sussan T, Biswal S, Mitzner W, Tuder RM, Armanios M. Telomere length is a determinant of emphysema susceptibility. *Am J Respir Crit Care Med* 2011; 184: 904-912.

40. Povedano JM, Martinez P, Flores JM, Mulero F, Blasco MA. Mice with Pulmonary Fibrosis Driven by Telomere Dysfunction. *Cell reports* 2015; 12: 286-299.

41. Kusko RL, Brothers JF, 2nd, Tedrow J, Pandit K, Huleihel L, Perdomo C, Liu G, Juan-Guardela B, Kass D, Zhang S, Lenburg M, Martinez F, Quackenbush J, Sciurba F, Limper A, Geraci M, Yang I, Schwartz DA, Beane J, Spira A, Kaminski N. Integrated Genomics Reveals Convergent Transcriptomic Networks Underlying Chronic Obstructive Pulmonary Disease and Idiopathic Pulmonary Fibrosis. *Am J Respir Crit Care Med* 2016; 194: 948-960.

42. Halu A, Liu S, Baek SH, Hobbs BD, Hunninghake GM, Cho MH, Silverman EK, Sharma A. Exploring the cross-phenotype network region of disease modules reveals concordant and

discordant pathways between chronic obstructive pulmonary disease and idiopathic pulmonary fibrosis. *Hum Mol Genet* 2019; 28: 2352-2364.

43. Hanaoka M, Ito M, Droma Y, Ushiki A, Kitaguchi Y, Yasuo M, Kubo K. Comparison of gene expression profiling between lung fibrotic and emphysematous tissues sampled from patients with combined pulmonary fibrosis and emphysema. *Fibrogenesis Tissue Repair* 2012; 5: 17.

44. Baumgartner KB, Samet JM, Stidley CA, Colby TV, Waldron JA. Cigarette smoking: a risk factor for idiopathic pulmonary fibrosis. *Am J Respir Crit Care Med* 1997; 155: 242-248.

45. Ekstrom M, Gustafson T, Boman K, Nilsson K, Tornling G, Murgia N, Toren K. Effects of smoking, gender and occupational exposure on the risk of severe pulmonary fibrosis: a population-based case-control study. *BMJ Open* 2014; 4: e004018.

46. Abramson MJ, Murambadoro T, Alif SM, Benke GP, Dharmage SC, Glaspole I, Hopkins P, Hoy RF, Klebe S, Moodley Y, Rawson S, Reynolds PN, Wolfe R, Corte TJ, Walters EH. Occupational and environmental risk factors for idiopathic pulmonary fibrosis in Australia: case-control study. *Thorax* 2020; 75: 864-869.

47. Bellou V, Belbasis L, Evangelou E. Tobacco Smoking and Risk for Pulmonary Fibrosis: A Prospective Cohort Study From the UK Biobank. *Chest* 2021; 160: 983-993.

48. Murphy SE, Park SL, Balbo S, Haiman CA, Hatsukami DK, Patel Y, Peterson LA, Stepanov I, Stram DO, Tretyakova N, Hecht SS, Le Marchand L. Tobacco biomarkers and genetic/epigenetic analysis to investigate ethnic/racial differences in lung cancer risk among smokers. *NPJ precision oncology* 2018; 2: 17.

49. Sundar IK, Rahman I. Gene expression profiling of epigenetic chromatin modification enzymes and histone marks by cigarette smoke: implications for COPD and lung cancer. *Am J Physiol Lung Cell Mol Physiol* 2016; 311: L1245-l1258.

50. Snetselaar R, van Moorsel CHM, Kazemier KM, van der Vis JJ, Zanen P, van Oosterhout MFM, Grutters JC. Telomere length in interstitial lung diseases. *Chest* 2015; 148: 1011-1018.

51. Arish N, Petukhov D, Wallach-Dayan SB. The Role of Telomerase and Telomeres in Interstitial Lung Diseases: From Molecules to Clinical Implications. *Int J Mol Sci* 2019; 20.

52. Hoffman TW, van Moorsel CHM, Borie R, Crestani B. Pulmonary phenotypes associated with genetic variation in telomere-related genes. *Curr Opin Pulm Med* 2018; 24: 269-280.

53. Ley B, Torgerson DG, Oldham JM, Adegunsoye A, Liu S, Li J, Elicker BM, Henry TS, Golden JA, Jones KD, Dressen A, Yaspan BL, Arron JR, Noth I, Hoffmann TJ, Wolters PJ. Rare Protein-Altering Telomere-related Gene Variants in Patients with Chronic Hypersensitivity Pneumonitis. *Am J Respir Crit Care Med* 2019; 200: 1154-1163.

54. Juge PA, Borie R, Kannengiesser C, Gazal S, Revy P, Wemeau-Stervinou L, Debray MP, Ottaviani S, Marchand-Adam S, Nathan N, Thabut G, Richez C, Nunes H, Callebaut I, Justet A, Leulliot N, Bonnefond A, Salgado D, Richette P, Desvignes JP, Lioté H, Froguel P, Allanore Y, Sand O, Dromer C, Flipo RM, Clément A, Béroud C, Sibilia J, Coustet B, Cottin V, Boissier MC, Wallaert B, Schaeverbeke T, Dastot le Moal F, Frazier A, Ménard C, Soubrier M, Saidenberg N, Valeyre D, Amselem S, Boileau C, Crestani B, Dieudé P. Shared genetic predisposition in rheumatoid arthritis-interstitial lung disease and familial pulmonary fibrosis. *Eur Respir J* 2017; 49.

55. Newton CA, Batra K, Torrealba J, Kozlitina J, Glazer CS, Aravena C, Meyer K, Raghu G, Collard HR, Garcia CK. Telomere-related lung fibrosis is diagnostically heterogeneous but uniformly progressive. *Eur Respir J* 2016; 48: 1710-1720.

56. Selman M, Martinez FJ, Pardo A. Why Does an Aging Smoker's Lung Develop Idiopathic Pulmonary Fibrosis and Not Chronic Obstructive Pulmonary Disease? *Am J Respir Crit Care Med* 2019; 199: 279-285.

57. Mora AL, Rojas M, Pardo A, Selman M. Emerging therapies for idiopathic pulmonary fibrosis, a progressive age-related disease. *Nature reviews Drug discovery* 2017; 16: 755-772.

58. Schafer MJ, White TA, Iijima K, Haak AJ, Ligresti G, Atkinson EJ, Oberg AL, Birch J, Salmonowicz H, Zhu Y, Mazula DL, Brooks RW, Fuhrmann-Stroissnigg H, Pirtskhalava T, Prakash YS, Tchkonia T, Robbins PD, Aubry MC, Passos JF, Kirkland JL, Tschumperlin DJ, Kita H, LeBrasseur NK. Cellular senescence mediates fibrotic pulmonary disease. *Nat Commun* 2017; 8: 14532.

59. Disayabutr S, Kim EK, Cha SI, Green G, Naikawadi RP, Jones KD, Golden JA, Schroeder A, Matthay MA, Kukreja J, Erle DJ, Collard HR, Wolters PJ. miR-34 miRNAs Regulate Cellular Senescence in Type II Alveolar Epithelial Cells of Patients with Idiopathic Pulmonary Fibrosis. *PLoS One* 2016; 11: e0158367.

60. Ahmad T, Sundar IK, Tormos AM, Lerner CA, Gerloff J, Yao H, Rahman I. Shelterin Telomere Protection Protein 1 Reduction Causes Telomere Attrition and Cellular Senescence via Sirtuin 1 Deacetylase in Chronic Obstructive Pulmonary Disease. *Am J Respir Cell Mol Biol* 2017; 56: 38-49.

61. Müller KC, Welker L, Paasch K, Feindt B, Erpenbeck VJ, Hohlfeld JM, Krug N, Nakashima M, Branscheid D, Magnussen H, Jörres RA, Holz O. Lung fibroblasts from patients with emphysema show markers of senescence in vitro. *Respir Res* 2006; 7: 32.

62. Chilosi M, Carloni A, Rossi A, Poletti V. Premature lung aging and cellular senescence in the pathogenesis of idiopathic pulmonary fibrosis and COPD/emphysema. *Translational research : the journal of laboratory and clinical medicine* 2013; 162: 156-173.

63. Hamsanathan S, Alder JK, Sellares J, Rojas M, Gurkar AU, Mora AL. Cellular Senescence: The Trojan Horse in Chronic Lung Diseases. *Am J Respir Cell Mol Biol* 2019; 61: 21-30.

64. Walsh SL, Wells AU, Sverzellati N, Devaraj A, von der Thüsen J, Yousem SA, Colby TV, Nicholson AG, Hansell DM. Relationship between fibroblastic foci profusion and high resolution CT morphology in fibrotic lung disease. *BMC medicine* 2015; 13: 241.

65. Oikonomou A, Mintzopoulou P, Tzouvelekis A, Zezos P, Zacharis G, Koutsopoulos A, Bouros D, Prassopoulos P. Pulmonary fibrosis and emphysema: Is the emphysema type associated with the pattern of fibrosis? *World J Radiol* 2015; 7: 294-305.

66. Jacob J, Song JW, Yoon HY, Cross G, Barnett J, Woo WL, Adams F, Kokosi M, Devaraj A, Renzoni E, Maher TM, Kim DS, Wells AU. Prevalence and Effects of Emphysema in Never-Smokers with Rheumatoid Arthritis Interstitial Lung Disease. *EBioMedicine* 2018; 28: 303-310.

67. Salvatore M, Singh A, Yip R, Fevrier E, Henschke CI, Yankelevitz D, Padilla M. Progression of probable UIP and UIP on HRCT. *Clin Imaging* 2019; 58: 140-144.

68. Agusti A, Faner R. Lung function trajectories in health and disease. *Lancet Respir Med* 2019; 7: 358-364.

69. Agustí A, Hogg JC. Update on the Pathogenesis of Chronic Obstructive Pulmonary Disease. *N Engl J Med* 2019; 381: 1248-1256.

70. Pardo A, Cabrera S, Maldonado M, Selman M. Role of matrix metalloproteinases in the pathogenesis of idiopathic pulmonary fibrosis. *Respir Res* 2016; 17: 23.

71. Segura-Valdez L, Pardo A, Gaxiola M, Uhal BD, Becerril C, Selman M. Upregulation of gelatinases A and B, collagenases 1 and 2, and increased parenchymal cell death in COPD. *Chest* 2000; 117: 684-694.

72. Houghton AM. Matrix metalloproteinases in destructive lung disease. *Matrix biology : journal of the International Society for Matrix Biology* 2015; 44-46: 167-174.

73. Manicone AM, Gharib SA, Gong KQ, Eddy WE, Long ME, Frevert CW, Altemeier WA, Parks WC, Houghton AM. Matrix Metalloproteinase-28 Is a Key Contributor to Emphysema Pathogenesis. *Am J Pathol* 2017; 187: 1288-1300.

74. Maldonado M, Salgado-Aguayo A, Herrera I, Cabrera S, Ortíz-Quintero B, Staab-Weijnitz CA, Eickelberg O, Ramírez R, Manicone AM, Selman M, Pardo A. Upregulation and Nuclear Location of MMP28 in Alveolar Epithelium of Idiopathic Pulmonary Fibrosis. *Am J Respir Cell Mol Biol* 2018; 59: 77-86.

75. Maldonado M, Buendía-Roldán I, Vicens-Zygmunt V, Planas L, Molina-Molina M, Selman M, Pardo A. Identification of MMP28 as a biomarker for the differential diagnosis of idiopathic pulmonary fibrosis. *PLoS One* 2018; 13: e0203779.

76. Selman M, Ruiz V, Cabrera S, Segura L, Ramírez R, Barrios R, Pardo A. TIMP-1, -2, -3, and -4 in idiopathic pulmonary fibrosis. A prevailing nondegradative lung microenvironment? *Am J Physiol Lung Cell Mol Physiol* 2000; 279: L562-574.

77. Pardo A, Barrios R, Gaxiola M, Segura-Valdez L, Carrillo G, Estrada A, Mejía M, Selman M. Increase of lung neutrophils in hypersensitivity pneumonitis is associated with lung fibrosis. *Am J Respir Crit Care Med* 2000; 161: 1698-1704.

78. Zuo F, Kaminski N, Eugui E, Allard J, Yakhini Z, Ben-Dor A, Lollini L, Morris D, Kim Y, DeLustro B, Sheppard D, Pardo A, Selman M, Heller RA. Gene expression analysis reveals matrilysin as a key regulator of pulmonary fibrosis in mice and humans. *Proc Natl Acad Sci U S A* 2002; 99: 6292-6297.

79. Herrera I, Cisneros J, Maldonado M, Ramírez R, Ortiz-Quintero B, Anso E, Chandel NS, Selman M, Pardo A. Matrix metalloproteinase (MMP)-1 induces lung alveolar epithelial cell migration and proliferation, protects from apoptosis, and represses mitochondrial oxygen consumption. *J Biol Chem* 2013; 288: 25964-25975.

80. Mattey DL, Nixon NB, Dawes PT. Association of circulating levels of MMP-8 with mortality from respiratory disease in patients with rheumatoid arthritis. *Arthritis Res Ther* 2012; 14: R204.

81. Gilligan DM, O'Connor CM, Ward K, Moloney D, Bresnihan B, FitzGerald MX. Bronchoalveolar lavage in patients with mild and severe rheumatoid lung disease. *Thorax* 1990; 45: 591-596.

82. Doyle TJ, Patel AS, Hatabu H, Nishino M, Wu G, Osorio JC, Golzarri MF, Traslosheros A, Chu SG, Frits ML, Iannaccone CK, Koontz D, Fuhrman C, Weinblatt ME, El-Chemaly SY, Washko GR, Hunninghake GM, Choi AM, Dellaripa PF, Oddis CV, Shadick NA, Ascherman DP, Rosas IO. Detection of Rheumatoid Arthritis-Interstitial Lung Disease Is Enhanced by Serum Biomarkers. *Am J Respir Crit Care Med* 2015; 191: 1403-1412.

83. Moinzadeh P, Krieg T, Hellmich M, Brinckmann J, Neumann E, Müller-Ladner U, Kreuter A, Dumitrescu D, Rosenkranz S, Hunzelmann N. Elevated MMP-7 levels in patients with systemic sclerosis: correlation with pulmonary involvement. *Exp Dermatol* 2011; 20: 770-773.

84. Andersen GN, Nilsson K, Pourazar J, Hackett TL, Kazzam E, Blomberg A, Waldenström A, Warner J, Rantapää-Dahlqvist S, Mincheva-Nilsson L, Sandström T. Bronchoalveolar matrix metalloproteinase 9 relates to restrictive lung function impairment in systemic sclerosis. *Respir Med* 2007; 101: 2199-2206.

85. Kennedy B, Branagan P, Moloney F, Haroon M, O'Connell OJ, O'Connor TM, O'Regan K, Harney S, Henry MT. Biomarkers to identify ILD and predict lung function decline in scleroderma lung disease or idiopathic pulmonary fibrosis. *Sarcoidosis Vasc Diffuse Lung Dis* 2015; 32: 228-236.

86. Manetti M, Guiducci S, Romano E, Bellando-Randone S, Conforti ML, Ibba-Manneschi L, Matucci-Cerinic M. Increased serum levels and tissue expression of matrix metalloproteinase-12 in patients with systemic sclerosis: correlation with severity of skin and pulmonary fibrosis and vascular damage. *Ann Rheum Dis* 2012; 71: 1064-1072.

87. Vuorinen K, Myllärniemi M, Lammi L, Piirilä P, Rytilä P, Salmenkivi K, Kinnula VL. Elevated matrilysin levels in bronchoalveolar lavage fluid do not distinguish idiopathic pulmonary fibrosis from other interstitial lung diseases. *Apmis* 2007; 115: 969-975.

88. Rosas IO, Richards TJ, Konishi K, Zhang Y, Gibson K, Lokshin AE, Lindell KO, Cisneros J, Macdonald SD, Pardo A, Sciurba F, Dauber J, Selman M, Gochuico BR, Kaminski N. MMP1 and MMP7 as potential peripheral blood biomarkers in idiopathic pulmonary fibrosis. *PLoS Med* 2008; 5: e93.

89. Ostridge K, Williams N, Kim V, Harden S, Bourne S, Coombs NA, Elkington PT, Estepar RS, Washko G, Staples KJ, Wilkinson TM. Distinct emphysema subtypes defined by quantitative CT analysis are associated with specific pulmonary matrix metalloproteinases. *Respir Res* 2016; 17: 92.

90. García-de-Alba C, Becerril C, Ruiz V, González Y, Reyes S, García-Alvarez J, Selman M, Pardo A. Expression of matrix metalloproteases by fibrocytes: possible role in migration and homing. *Am J Respir Crit Care Med* 2010; 182: 1144-1152.

91. García de Alba C, Buendia-Roldán I, Salgado A, Becerril C, Ramírez R, González Y, Checa M, Navarro C, Ruiz V, Pardo A, Selman M. Fibrocytes contribute to inflammation and fibrosis in chronic hypersensitivity pneumonitis through paracrine effects. *Am J Respir Crit Care Med* 2015; 191: 427-436.

92. Andersson-Sjöland A, de Alba CG, Nihlberg K, Becerril C, Ramírez R, Pardo A, Westergren-Thorsson G, Selman M. Fibrocytes are a potential source of lung fibroblasts in idiopathic pulmonary fibrosis. *Int J Biochem Cell Biol* 2008; 40: 2129-2140.

93. Dupin I, Thumerel M, Maurat E, Coste F, Eyraud E, Begueret H, Trian T, Montaudon M, Marthan R, Girodet PO, Berger P. Fibrocyte accumulation in the airway walls of COPD patients. *Eur Respir J* 2019; 54.

94. de Brouwer B, Drent M, van den Ouweland JMW, Wijnen PA, van Moorsel CHM, Bekers O, Grutters JC, White ES, Janssen R. Increased circulating desmosine and age-dependent elastinolysis in idiopathic pulmonary fibrosis. *Respir Res* 2018; 19: 45.

95. Sato T, Kajikuri T, Saito Y, Chikuma M, Nagai S. Determination of desmosine in bronchoalveolar lavage fluids by time-resolved fluoroimmunoassay. *Clin Chim Acta* 2008; 387: 113-119.

96. Kim C, Ko Y, Kim SH, Yoo HJ, Lee JS, Rhee CK, Lee JH, Lee JH, Kim TH, Lim SY, Yoo KH, Seo JB, Oh YM, Lee SD, Park YB. Urinary desmosine is associated with emphysema severity and frequent exacerbation in patients with COPD. *Respirology* 2018; 23: 176-181.

97. Turino GM. Chronic Obstructive Pulmonary Disease. A Biomarker and a Potential Therapy. *Ann Am Thorac Soc* 2018; 15: S15-s17.

98. Gregory AD, Kliment CR, Metz HE, Kim KH, Kargl J, Agostini BA, Crum LT, Oczypok EA, Oury TA, Houghton AM. Neutrophil elastase promotes myofibroblast differentiation in lung fibrosis. *J Leukoc Biol* 2015; 98: 143-152.

99. Hoyle GW, Li J, Finkelstein JB, Eisenberg T, Liu JY, Lasky JA, Athas G, Morris GF, Brody AR. Emphysematous lesions, inflammation, and fibrosis in the lungs of transgenic mice overexpressing platelet-derived growth factor. *Am J Pathol* 1999; 154: 1763-1775.

100. Glasser SW, Detmer EA, Ikegami M, Na CL, Stahlman MT, Whitsett JA. Pneumonitis and emphysema in sp-C gene targeted mice. *J Biol Chem* 2003; 278: 14291-14298.

101. Fraser E, St Noble V, Hoyles RK, Benamore R, Ho LP. Readily accessible CT scoring method to quantify fibrosis in IPF. *BMJ open respiratory research* 2020; 7.

102. Jacob J, Bartholmai BJ, Rajagopalan S, Kokosi M, Maher TM, Nair A, Karwoski R, Renzoni E, Walsh SLF, Hansell DM, Wells AU. Functional and prognostic effects when emphysema complicates idiopathic pulmonary fibrosis. *Eur Respir J* 2017; 50: 1700379.

103. Jankowich MD, Rounds S. Combined pulmonary fibrosis and emphysema alters physiology but has similar mortality to pulmonary fibrosis without emphysema. *Lung* 2010; 188: 365-373.

104. Kishaba T, Shimaoka Y, Fukuyama H, Yoshida K, Tanaka M, Yamashiro S, Tamaki H. A cohort study of mortality predictors and characteristics of patients with combined pulmonary fibrosis and emphysema. *BMJ Open* 2012; 2.

105. Bodlet A, Maury G, Jamart J, Dahlqvist C. Influence of radiological emphysema on lung function test in idiopathic pulmonary fibrosis. *Respir Med* 2013; 107: 1781-1788.

106. Alsumrain M, De Giacomi F, Nasim F, Koo CW, Bartholmai BJ, Levin DL, Moua T. Combined pulmonary fibrosis and emphysema as a clinicoradiologic entity: Characterization of presenting lung fibrosis and implications for survival. *Respir Med* 2019; 146: 106-112.

107. Matsuoka S, Yamashiro T, Matsushita S, Fujikawa A, Kotoku A, Yagihashi K, Kurihara Y, Nakajima Y. Morphological disease progression of combined pulmonary fibrosis and emphysema: comparison with emphysema alone and pulmonary fibrosis alone. *J Comput Assist Tomogr* 2015; 39: 153-159.

108. Kitaguchi Y, Fujimoto K, Hanaoka M, Kawakami S, Honda T, Kubo K. Clinical characteristics of combined pulmonary fibrosis and emphysema. *Respirology* 2010; 15: 265-271.

109. Mitchell PD, Das JP, Murphy DJ, Keane MP, Donnelly SC, Dodd JD, Butler MW. Idiopathic pulmonary fibrosis with emphysema: evidence of synergy among emphysema and idiopathic pulmonary fibrosis in smokers. *Respir Care* 2015; 60: 259-268.

110. Sugino K, Nakamura Y, Ito T, Isshiki T, Sakamoto S, Homma S. Comparison of clinical characteristics and outcomes between combined pulmonary fibrosis and emphysema associated with usual interstitial pneumonia pattern and non-usual interstitial pneumonia. *Sarcoidosis Vasc Diffuse Lung Dis* 2015; 32: 129-137.

111. Kinoshita Y, Watanabe K, Ishii H, Kushima H, Fujita M, Nabeshima K. Distribution of emphysema and fibrosis in idiopathic pulmonary fibrosis with coexisting emphysema. *Histopathology* 2019; 74: 1103-1108.

112. Mori K, Shirai T, Mikamo M, Shishido Y, Akita T, Morita S, Asada K, Fujii M, Hozumi H, Suda T, Chida K. Respiratory mechanics measured by forced oscillation technique in combined pulmonary fibrosis and emphysema. *Respiratory physiology & neurobiology* 2013; 185: 235-240.

113. Sato S, Tanino Y, Misa K, Fukuhara N, Nikaido T, Uematsu M, Fukuhara A, Wang X, Ishida T, Munakata M. Identification of Clinical Phenotypes in Idiopathic Interstitial Pneumonia with Pulmonary Emphysema. *Intern Med* 2016; 55: 1529-1535.

114. Kim YS, Jin GY, Chae KJ, Han YM, Chon SB, Lee YS, Kwon KS, Choi HM. Visually stratified CT honeycombing as a survival predictor in combined pulmonary fibrosis and emphysema. *Br J Radiol* 2015; 88: 20150545.

115. Kitaguchi Y, Fujimoto K, Hanaoka M, Honda T, Hotta J, Hirayama J. Pulmonary function impairment in patients with combined pulmonary fibrosis and emphysema with and without airflow obstruction. *Int J Chron Obstruct Pulmon Dis* 2014; 9: 805-811.

116. Ryerson CJ, Hartman T, Elicker BM, Ley B, Lee JS, Abbritti M, Jones KD, King TE, Jr., Ryu J, Collard HR. Clinical features and outcomes in combined pulmonary fibrosis and emphysema in idiopathic pulmonary fibrosis. *Chest* 2013; 144: 234-240.

117. Cottin V, Hansell DM, Sverzellati N, Weycker D, Antoniou KM, Atwood M, Oster G, Kirchgaessler KU, Collard HR, Wells AU. Effect of Emphysema Extent on Serial Lung Function in Patients with Idiopathic Pulmonary Fibrosis. *Am J Respir Crit Care Med* 2017; 196: 1162-1171.

118. Tzouvelekis A, Zacharis G, Oikonomou A, Mikroulis D, Margaritopoulos G, Koutsopoulos A, Antoniadis A, Koulelidis A, Steiropoulos P, Boglou P, Bakali M, Froudarakis M, Bouros D.

Increased incidence of autoimmune markers in patients with combined pulmonary fibrosis and emphysema. *BMC Pulm Med* 2013; 13: 31.

119. Chae KJ, Jin GY, Han YM, Kim YS, Chon SB, Lee YS, Kwon KS, Choi HM, Lynch D. Prevalence and progression of combined pulmonary fibrosis and emphysema in asymptomatic smokers: A case-control study. *Eur Radiol* 2015; 25: 2326-2334.

120. Ando K, Sekiya M, Tobino K, Takahashi K. Relationship between quantitative CT metrics and pulmonary function in combined pulmonary fibrosis and emphysema. *Lung* 2013; 191: 585-591.

121. Awano N, Inomata M, Ikushima S, Yamada D, Hotta M, Tsukuda S, Kumasaka T, Takemura T, Eishi Y. Histological analysis of vasculopathy associated with pulmonary hypertension in combined pulmonary fibrosis and emphysema: comparison with idiopathic pulmonary fibrosis or emphysema alone. *Histopathology* 2017; 70: 896-905.

122. Kurashima K, Takayanagi N, Tsuchiya N, Kanauchi T, Ueda M, Hoshi T, Miyahara Y, Sugita Y. The effect of emphysema on lung function and survival in patients with idiopathic pulmonary fibrosis. *Respirology* 2010; 15: 843-848.

123. Todd NW, Jeudy J, Lavania S, Franks TJ, Galvin JR, Deepak J, Britt EJ, Atamas SP. Centrilobular emphysema combined with pulmonary fibrosis results in improved survival. *Fibrogenesis Tissue Repair* 2011; 4: 6.

124. Lee G, Kim KU, Lee JW, Suh YJ, Jeong YJ. Serial changes and prognostic implications of CT findings in combined pulmonary fibrosis and emphysema: comparison with fibrotic idiopathic interstitial pneumonias alone. *Acta Radiol* 2017; 58: 550-557.

125. Chae KJ, Jin GY, Jung HN, Kwon KS, Choi H, Lee YC, Chung MJ, Park HS. Differentiating Smoking-Related Interstitial Fibrosis (SRIF) from Usual Interstitial Pneumonia (UIP) with Emphysema Using CT Features Based on Pathologically Proven Cases. *PLoS One* 2016; 11: e0162231.

126. Lai RS, Chen CF, Chu KA, Lin MH. The effect of emphysema on survival in patients with idiopathic pulmonary fibrosis: A retrospective study in Taiwan. *Journal of the Chinese Medical Association : JCMA* 2019; 82: 922-928.

127. Tokgoz Akyıl F, Sevim T, Akman C, Aksoy E, Ağca M, Aktas O, Akyıl M. The predictors of mortality in IPF - Does emphysema change the prognosis? *Sarcoidosis Vasc Diffuse Lung Dis* 2016; 33: 267-274.

128. Inomata M, Ikushima S, Awano N, Kondoh K, Satake K, Masuo M, Kusunoki Y, Moriya A, Kamiya H, Ando T, Yanagawa N, Kumasaka T, Ogura T, Sakai F, Azuma A, Gemma A, Takemura T. An autopsy study of combined pulmonary fibrosis and emphysema: correlations among clinical, radiological, and pathological features. *BMC Pulm Med* 2014; 14: 104.

129. Kwak N, Park CM, Lee J, Park YS, Lee SM, Yim JJ, Yoo CG, Kim YW, Han SK, Lee CH. Lung cancer risk among patients with combined pulmonary fibrosis and emphysema. *Respir Med* 2014; 108: 524-530.

130. Minegishi Y, Kokuho N, Miura Y, Matsumoto M, Miyanaga A, Noro R, Saito Y, Seike M, Kubota K, Azuma A, Kida K, Gemma A. Clinical features, anti-cancer treatments and outcomes of lung cancer patients with combined pulmonary fibrosis and emphysema. *Lung Cancer* 2014; 85: 258-263.

131. Zhang M, Yoshizawa A, Kawakami S, Asaka S, Yamamoto H, Yasuo M, Agatsuma H, Toishi M, Shiina T, Yoshida K, Honda T, Ito KI. The histological characteristics and clinical outcomes of lung cancer in patients with combined pulmonary fibrosis and emphysema. *Cancer Med* 2016; 5: 2721-2730.

Supplementary figures

Figure E1. Systematic review search results.

Articles were excluded if the study was not original research, not combined pulmonary fibrosis and emphysema-related, had a sample size < 10, was non-English, or involved non-human subjects (from reference (3)). The 14 additional articles were identified by reviewing reference lists of eligible full-text manuscripts. CPFE = combined pulmonary fibrosis and emphysema.

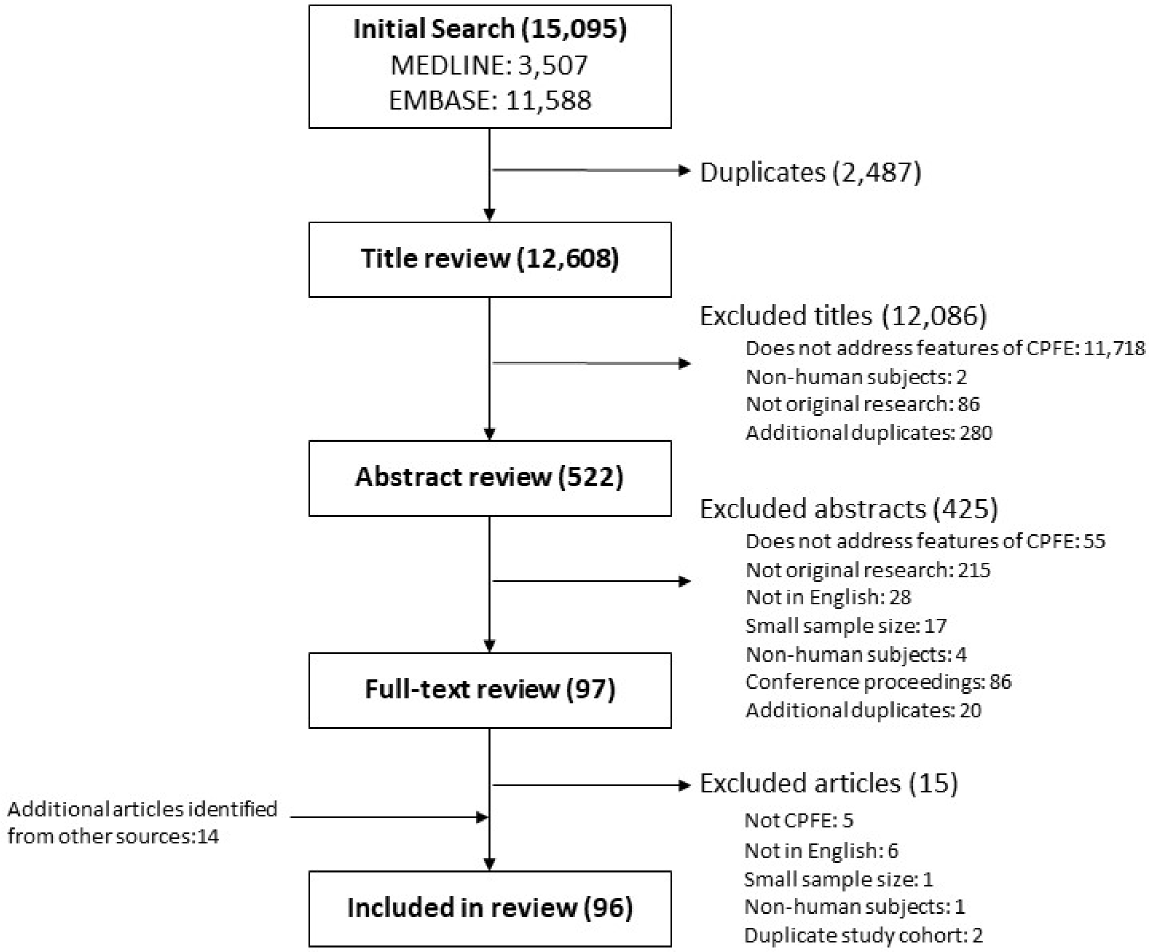

Figure E2. Emphysematous lesions in patients with lung fibrosis who have never smoked.

Upper panel: an axial CT image in a 48-year-old female patient diagnosed with rheumatoid arthritis-related interstitial lung disease demonstrates subtle diffuse low attenuation bilaterally (arrows). Middle panel: in a 34-year-old male patient diagnosed with scleroderma, focal areas of low attenuation are evident bilaterally (arrows). Lower panel: a 49-year-old male patient diagnosed with hypersensitivity pneumonitis demonstrates widespread coalescent low attenuation bilaterally in keeping with emphysema-like parenchymal destruction.

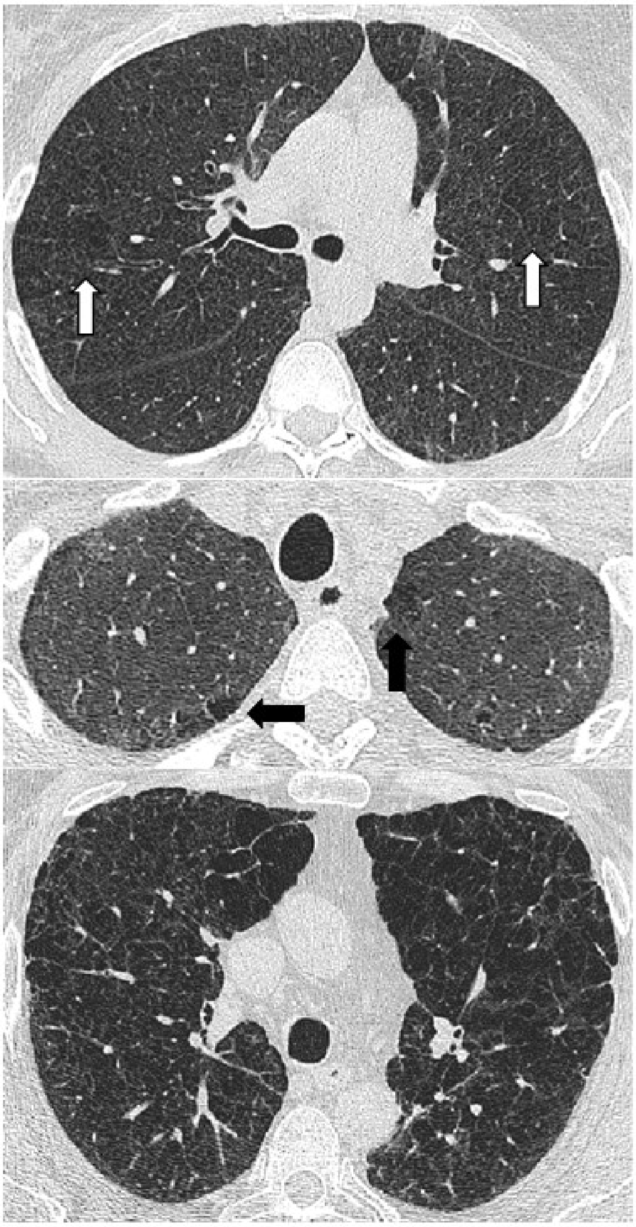

Figure E3. Minimal extent of pulmonary fibrosis and emphysema on CT imaging.

Patient A (upper panel) has extensive emphysema in the upper lobes (arrow) and minimal fibrosis evidenced by reticulation and traction bronchiectasis in the middles and lower zones. Patient B (lower panel) has minimal paraseptal emphysema in the lung apices (arrows) but extensive fibrosis evidenced throughout the middle and lower zones. Determining minimal thresholds of emphysema and fibrosis that would qualify a patient for consideration within the combined pulmonary fibrosis and emphysema (CPFE) phenotype remains a challenge that needs to be addressed.

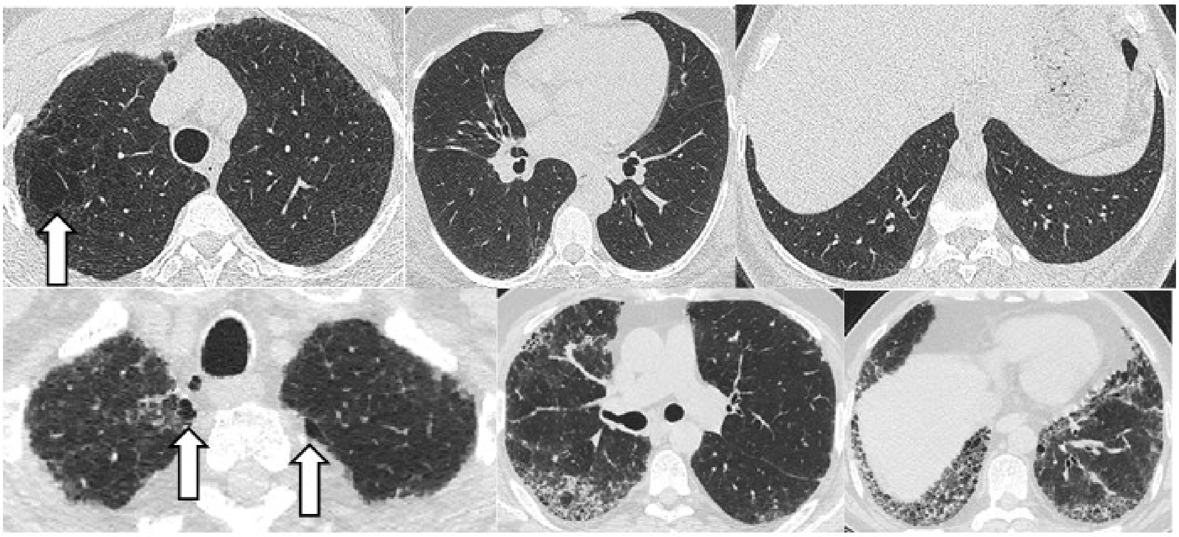

Figure E4. Challenges in quantifying the fibrosis extent on HRCT.

Challenges in quantifying the fibrosis extent on HRCT. Quantifying fibrosis extent in patients with combined pulmonary fibrosis and emphysema (CPFE) requires clear definitions on parenchymal patterns that should be considered as contributing to the total extent of fibrosis. In patients with idiopathic pulmonary fibrosis (upper panel), ground glass opacity when intermixed with reticular lines and traction bronchiectasis (arrows) usually represents fine fibrosis where the individual components of the fibrosis are beyond the resolution of CT imaging. In patients with hypersensitivity pneumonitis or connective tissue-related interstitial lung disease however, ground glass opacity might represent transient inflammation rather than fibrosis. In such cases, ground glass opacity should probably not be included within the quantified volume of fibrotic lung parenchyma. When ground glass opacity lies distant to regions of fibrosis i.e. away from areas of reticulation and traction bronchiectasis (lower panel, arrows), even in patients with idiopathic pulmonary fibrosis, an inflammatory insult complicating the disease process should be considered. Complications may include infection, aspiration, pulmonary oedema or an acute exacerbation of interstitial disease. Here again, the ground glass opacity should not be considered as part of the fibrotic lung volume.

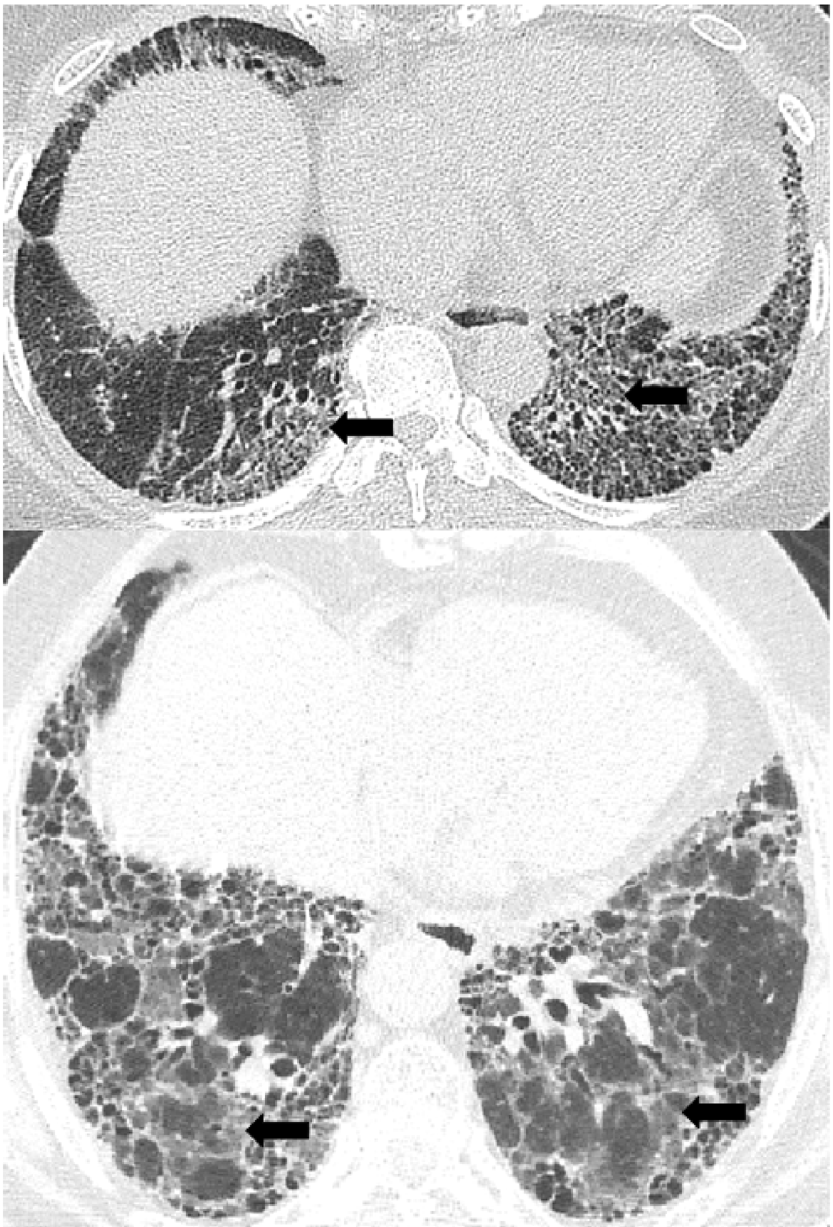

Figure E5. Respiratory bronchiolitis (RB) and smoking-related interstitial fibrosis (SRIF) in a patient with ILD.

A. Low magnification photomicrograph showing RB characterized by intraluminal lightly pigmented macrophages with associated SRIF that expands peribronchiolar alveolar septa. B. Higher magnification photomicrograph showing expansion of peribronchiolar interstitium by mild non-distorting fibrosis that contiguously extends into peribronchiolar alveolar septa. Hematoxylin and eosin stain.

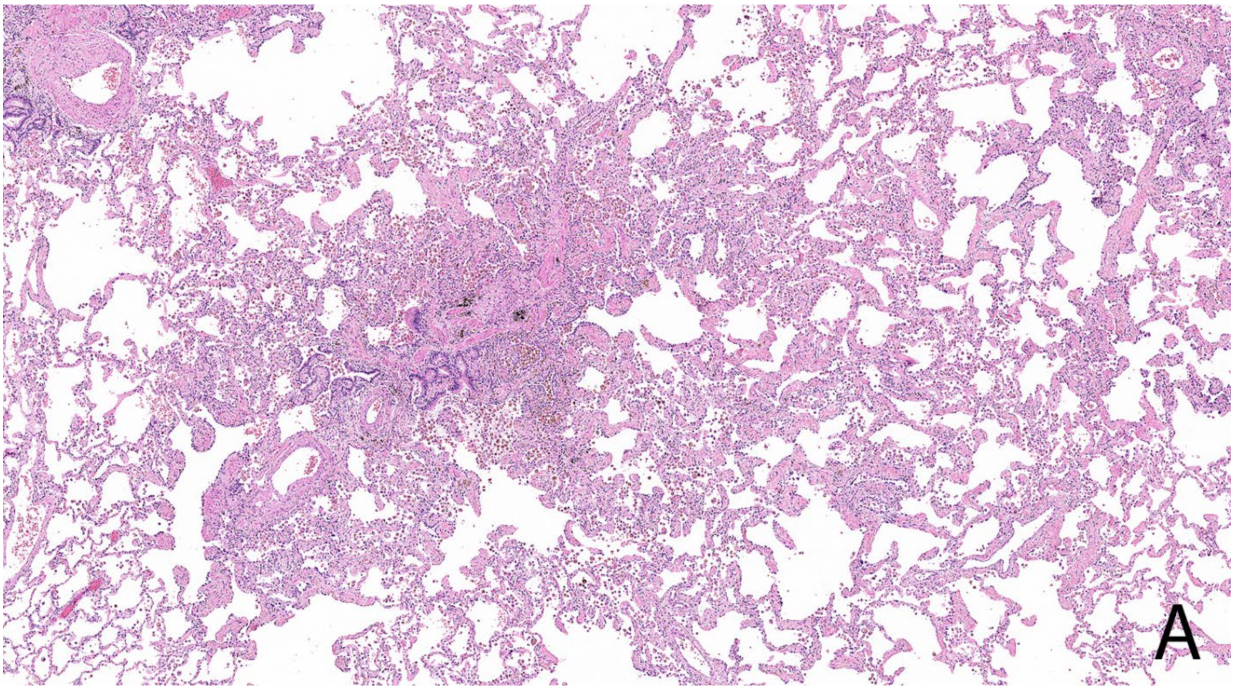

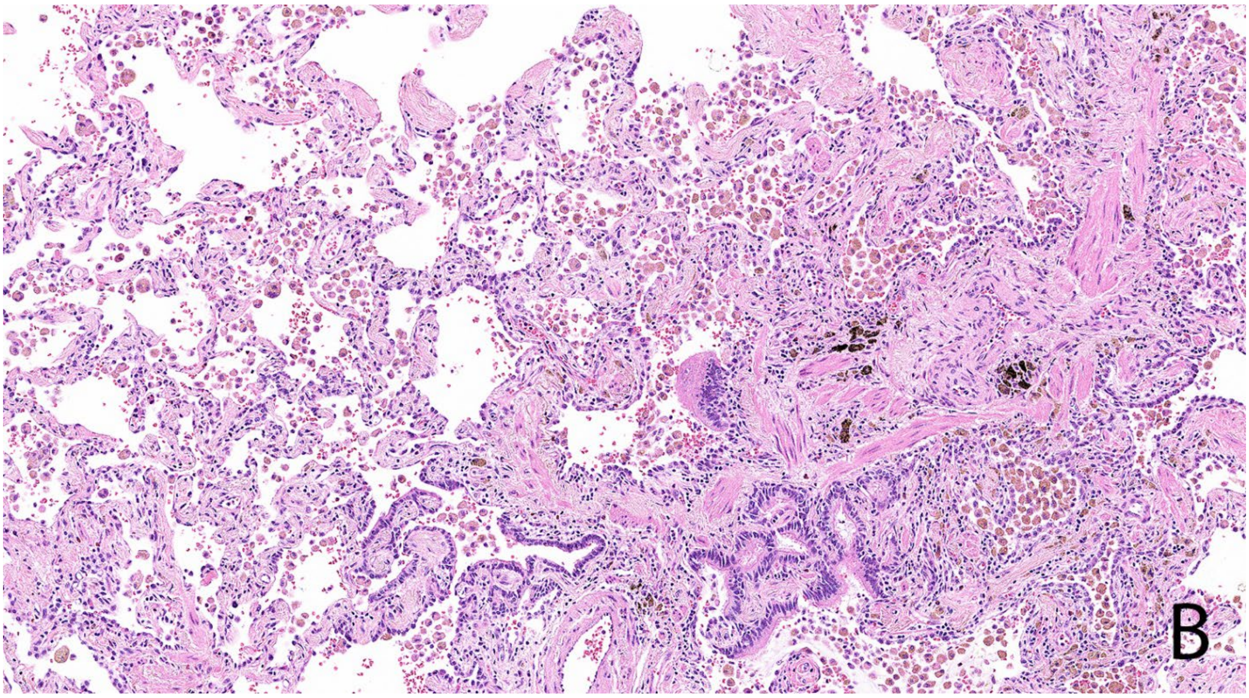

Figure E6. Smoking-related interstitial fibrosis (SRIF) and extensive respiratory bronchiolitis (RB) resembling desquamative interstitial pneumonia (DIP).

A. Low magnification photomicrograph showing an area in which SRIF is more diffuse and uniform in its distribution. Alveolar septa are expanded by paucicellular collagen deposition with preservation of lung architecture. Alveolar spaces show prominent clusters of pigmented macrophages (RB) resulting in a pattern closely resembling DIP. B. Higher magnification view demonstrates the densely eosinophilic collagen and pigmented alveolar macrophages typical of SRIF and RB, respectively. Hematoxylin and eosin stain.

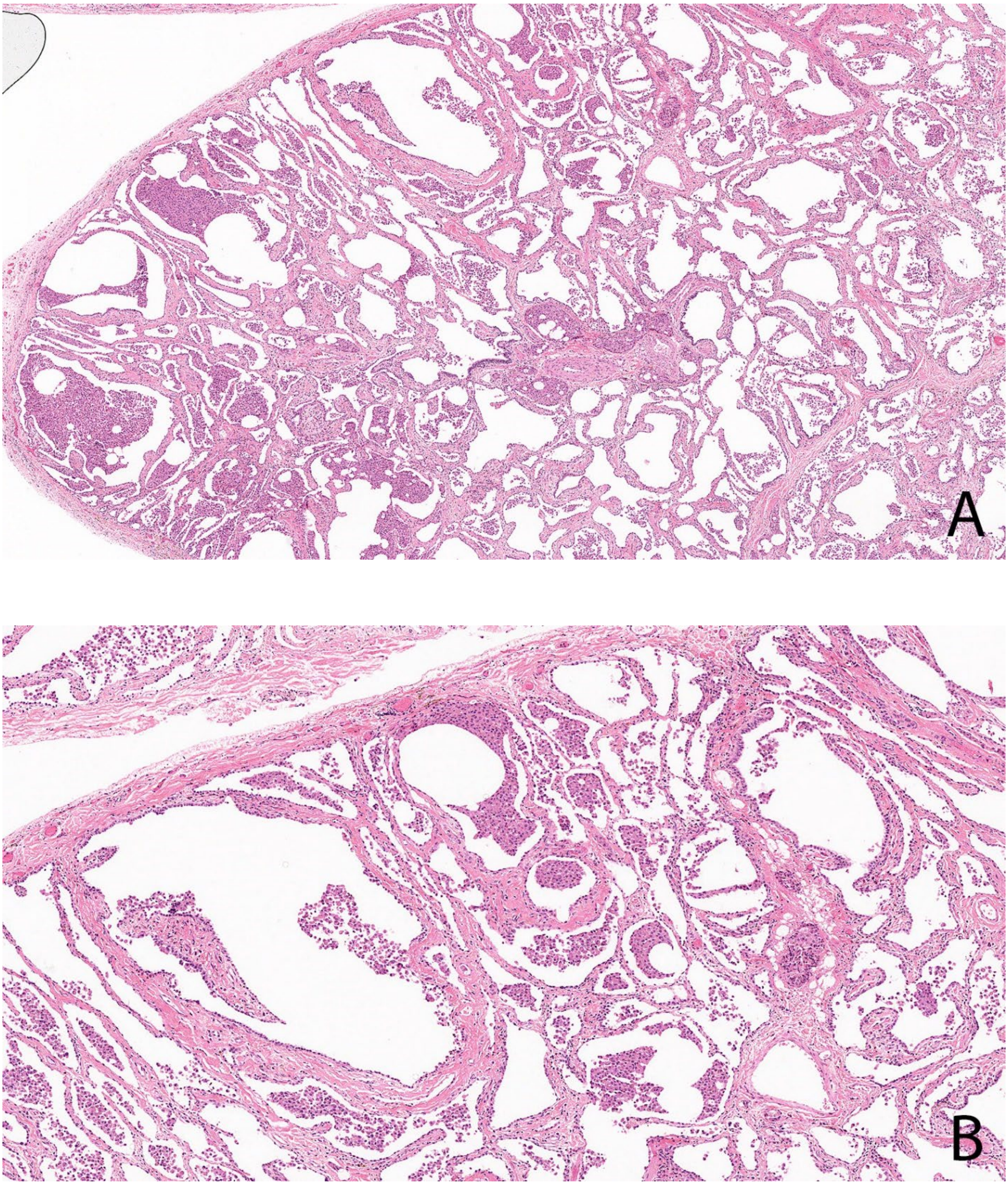

Figure E7. Advanced Langerhans cell histiocytosis (LCH) mimicking combined pulmonary fibrosis and emphysema (CPFE).

A. Photograph showing the cut surface of a formalin fixed lung explant from a patient with advanced LCH. Cystic change preferentially affects the upper lobe in a distribution resembling centriacinar emphysema. B. Low magnification photomicrograph of surgical lung biopsy showing combination of predominantly fibrotic LCH and characteristic pattern of associated paracicatricial airspace enlargement ("scar emphysema"). The paracicatricial airspace enlargement comprises cystic airspaces adjacent to bronchiolocentric stellate scars that mark the sites of previously cellular lesions. Only a single lesion in this field contains residual islands of Langerhans cells (*). C. Higher magnification photomicrograph from same biopsy showing fibrotic LCH with associated airspace enlargement mimicking centriacinar emphysema with fibrosis. Stellated nodules (*) with associated airspace enlargement are key to separating fibrotic LCH from other patterns of fibrosis. Note that nodule on left includes fibrosis extending to visceral pleura forming thick-walled cysts resembling those seen in SRIF (see Figure 12). Hematoxylin and eosin stain.

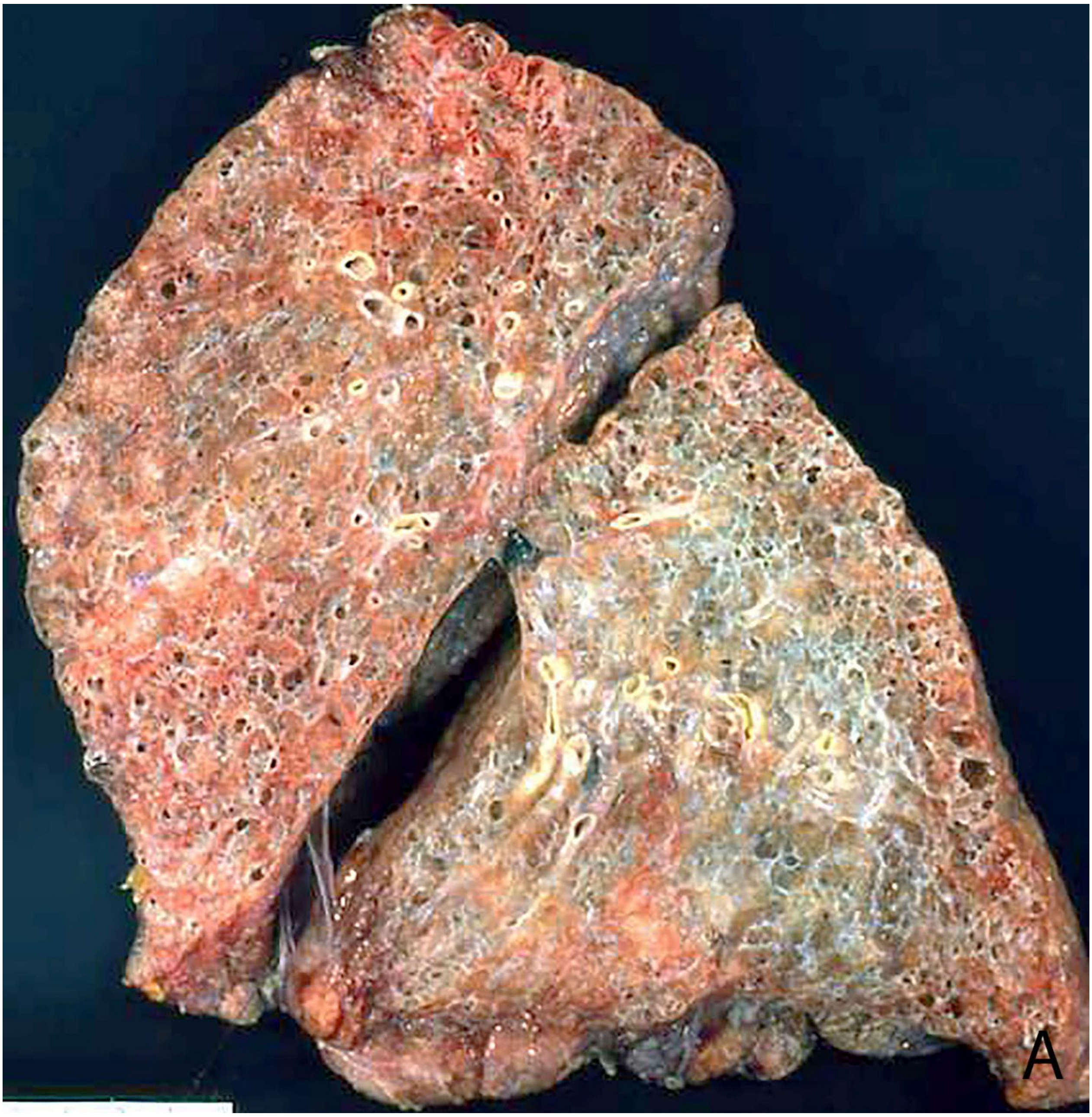

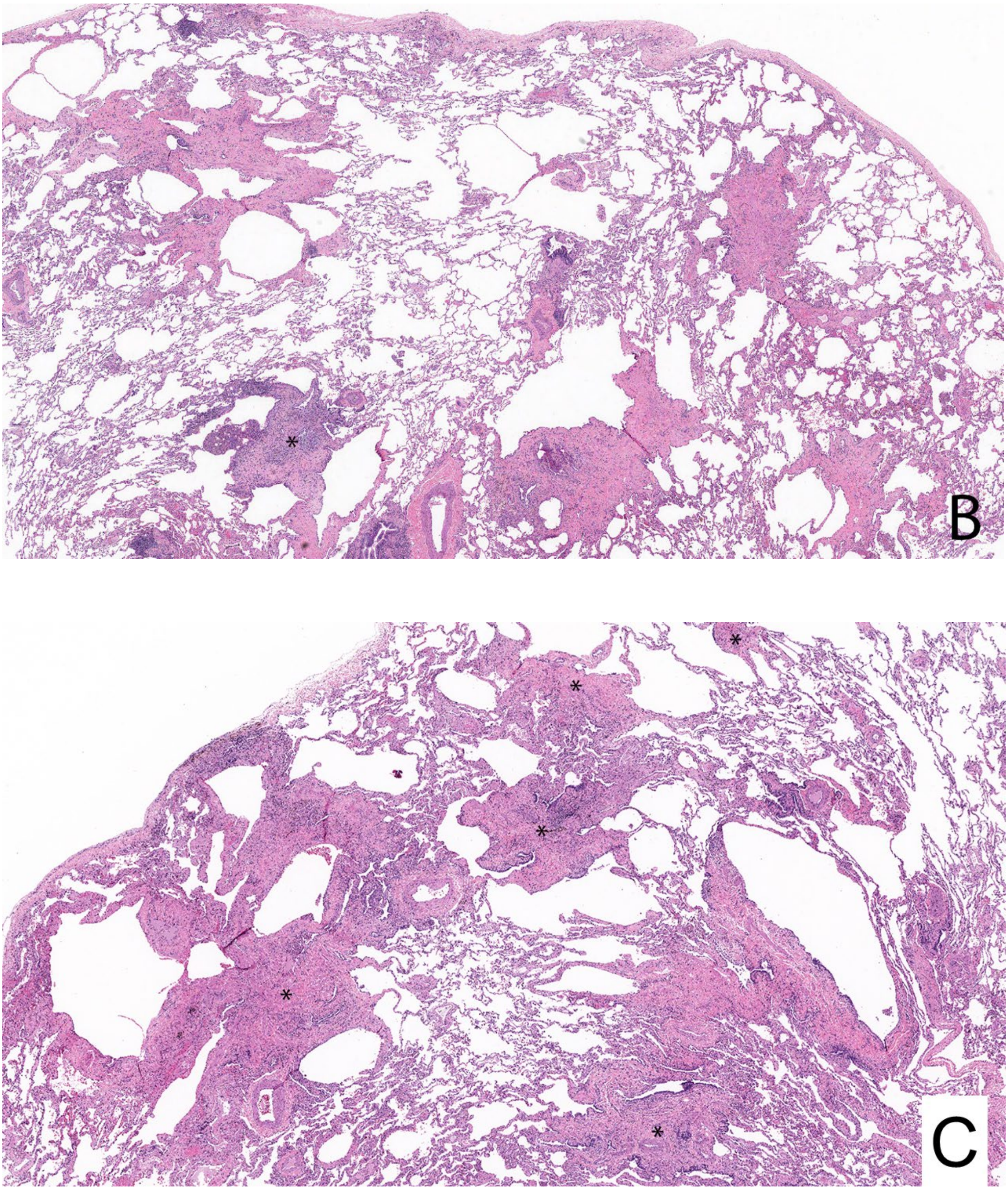
B
C

Figure E8. Comparison of smoking related interstitial fibrosis (SRIF) and usual interstitial pneumonia (UIP).

A. Low magnification photomicrograph showing SRIF in surgical lung biopsy. There is mild expansion of subpleural alveolar septa by paucicellular densely eosinophilic collagen deposition (arrows) without tissue distorting scars or honeycomb change. There is similarly paucicellular fibrosis involving peribronchiolar interstitium (*) in a distribution that overlaps with descriptions of AEF. B. Low magnification photomicrograph showing a UIP pattern in which the fibrosis is more advanced with a characteristic "patchwork" distribution, predilection for peripheral subpleural parenchyma, and associated scarring and honeycomb change (*). Hematoxylin and eosin stain.

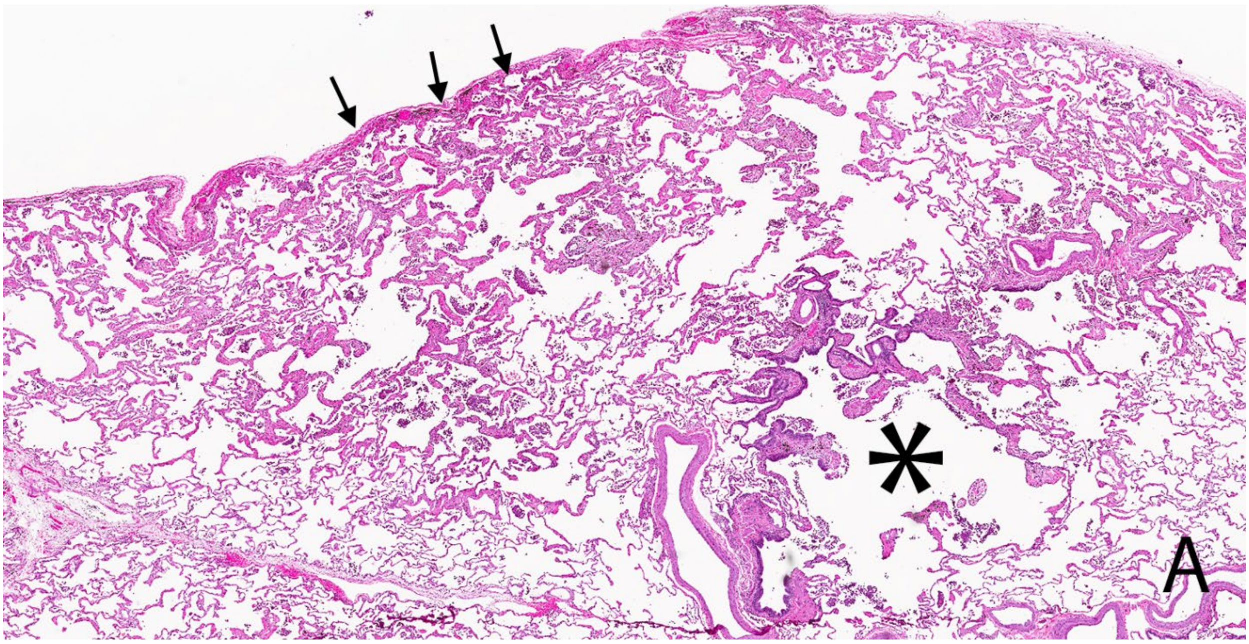

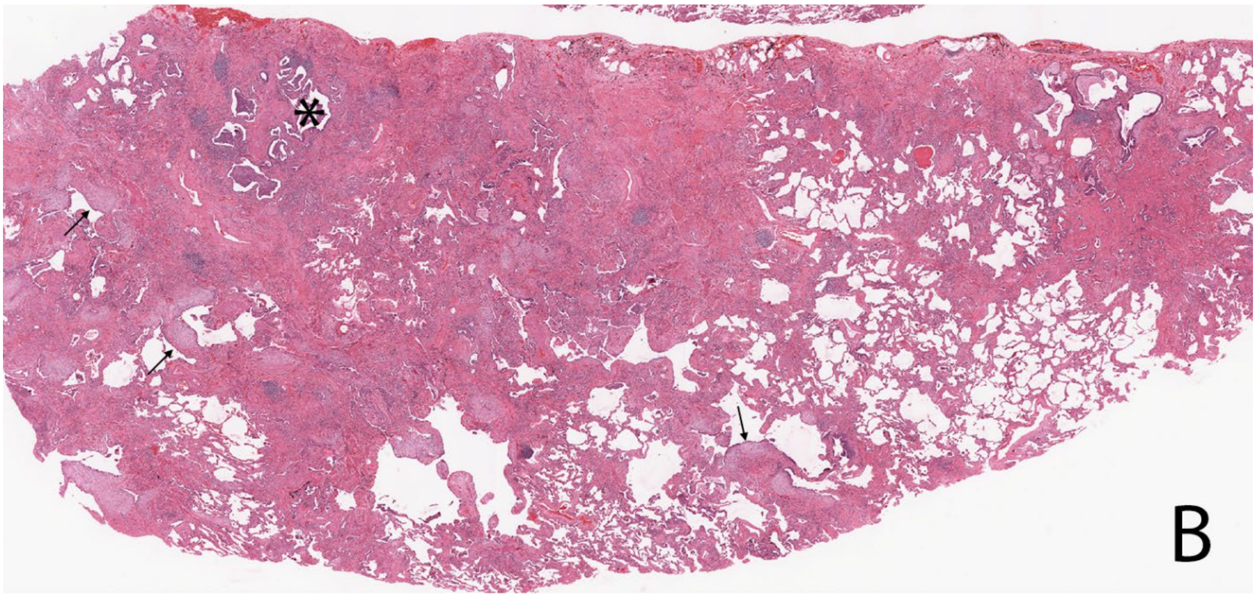

Figure E9. Usual interstitial pneumonia (UIP) and emphysema in a patient with combined pulmonary fibrosis and emphysema (CPFE).

A. Low magnification photomicrograph of surgical lung biopsy showing patchy fibrosis and emphysema in a heavy smoker with a history of COPD and progressive ILD who presented with pneumothorax. B. Low magnification photomicrograph of surgical lung biopsy from same patient showing patchy fibrosis that includes honeycomb change (*) and fibroblast foci (arrow) characteristic of UIP. C. Higher magnification view of same field showing honeycomb change (*) and fibroblast focus (arrow). D. CPFE in this patient complicated by paraseptal emphysema and pleural blebs (*) which accounted for pneumothorax risk. Hematoxylin and eosin stain.

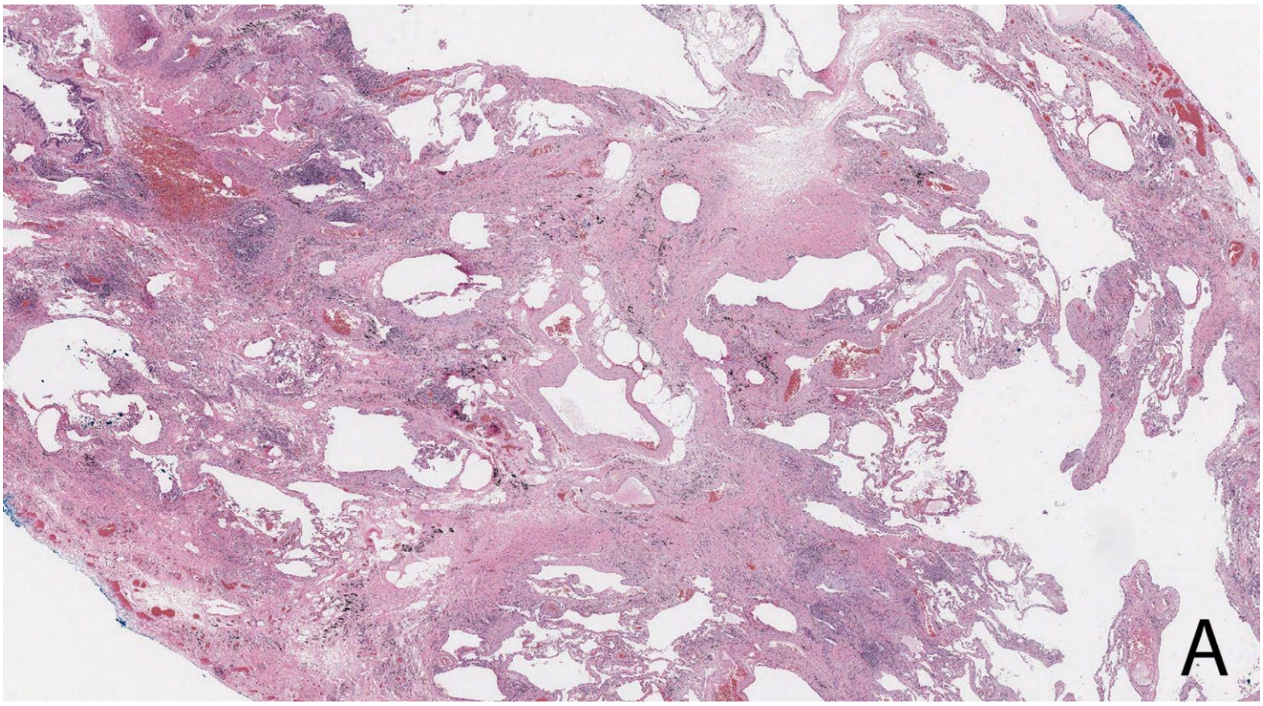

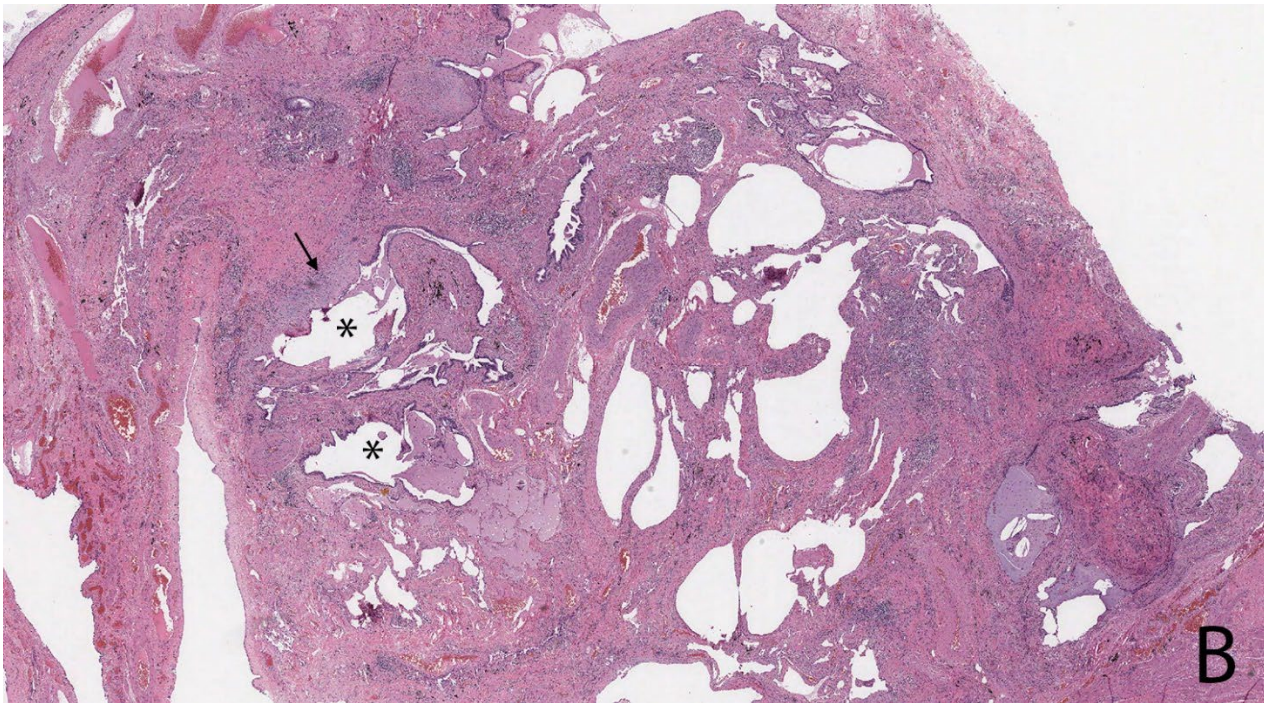

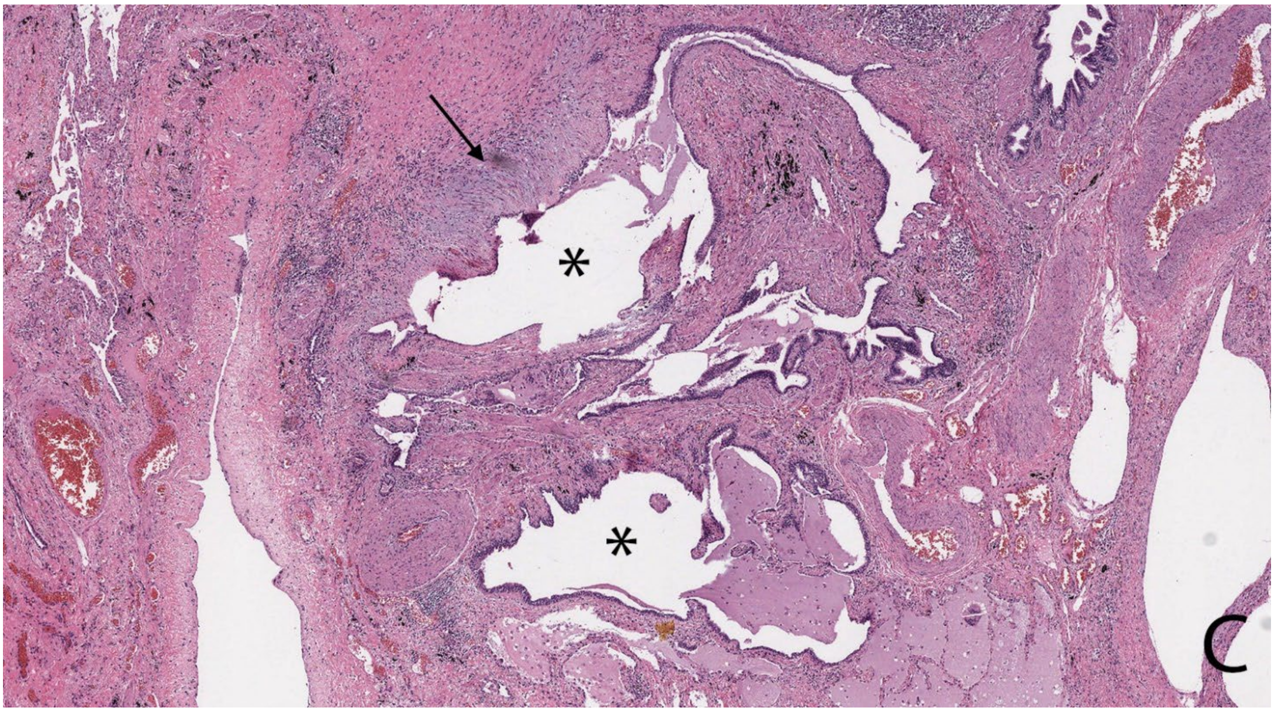
C

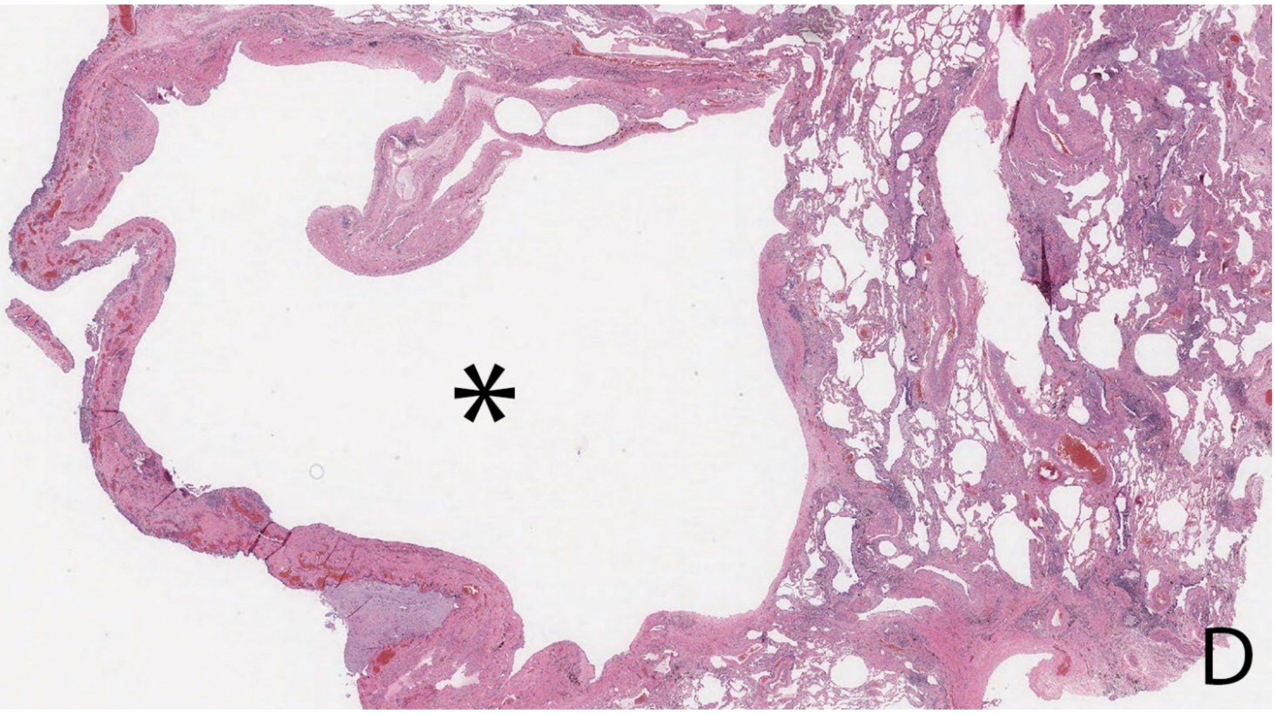
D

Figure E10. Definition and thresholds used to define CPFE.

A threshold of "0" indicates studies that defined the presence of the abnormality based on patients having any amount of that abnormality.

Features	Pulmonary fibrosis	Emphysema
**Required radiologic features**	Diffuse parenchymal lung disease with significant pulmonary fibrosis, defined as all of the following:<br>• Reticulation with peripheral and basal predominance<br>• Honeycombing<br>• Architectural distortion<br>• Minimal ground glass and/or consolidation	Areas of decreased attenuation with thin (<1 mm) or no walls<br>• Upper zone predominant
**Required minimum extent of abnormality on visual assessment**	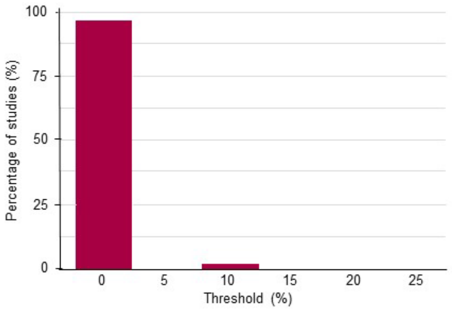	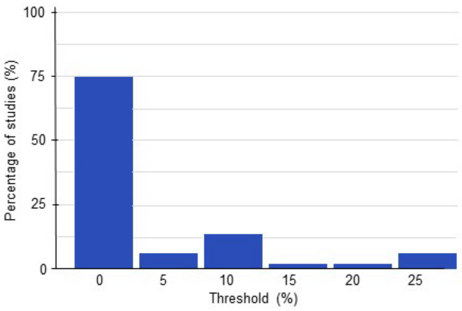
